# Genetic heterogeneity in childhood leukemia/lymphoma: a Turkish cohort with strong predisposition

**DOI:** 10.3389/fgene.2025.1624306

**Published:** 2025-09-09

**Authors:** Gizem Onder, Ozkan Ozdemir, Fulya Taylan, Cengiz Canpolat, Koray Yalcin, Fatih Erbey, Banu Oflaz Sozmen, Fikret Asarcikli, Turan Bayhan, Yunus Murat Akcabelen, Nese Yarali, Namik Yasar Ozbek, Ikbal Ok Bozkaya, Dilek Kacar, Berk Ergun, Alper Akkus, Davut Albayrak, Elif Ince, Ugur Demirsoy, Gul Nihal Ozdemir, Omer Dogru, Seda Aras, Eylul Aydin, Busra Unal, Ufuk Amanvermez, Ozlem Akgun Dogan, Sezer Akyoney, Muge Sayitoglu, Ann Nordgren, Nihat Bugra Agaoglu, Ugur Ozbek, Ozden Hatirnaz Ng

**Affiliations:** ^1^ Department of Biochemistry and Molecular Biology, Health Sciences Institute, Acıbadem Mehmet Ali Aydınlar University, Istanbul, Türkiye; ^2^ Rare Diseases and Orphan Drugs Application and Research Center (ACURARE), Rare Diseases and Orphan Drugs Application and Research Center (ACURARE), Acıbadem University, Istanbul, Türkiye; ^3^ Department of Molecular Medicine and Surgery, Karolinska Institutet, Stockholm, Sweden; ^4^ Department of Medical Biology, School of Medicine, Acıbadem Mehmet Ali Aydınlar University, Istanbul, Türkiye; ^5^ Department of Clinical Genetics and Genomics, Karolinska University Hospital, Stockholm, Sweden; ^6^ Department of Pediatric Oncology, School of Medicine, Acıbadem Mehmet Ali Aydınlar University, Istanbul, Türkiye; ^7^ Department of Pediatric Hematology, Bahçeşehir University, Goztepe Medical Park Hospital, Istanbul, Türkiye; ^8^ Department of Medical Biotechnology, Health Sciences Institute, Acıbadem Mehmet Ali Aydınlar University, Istanbul, Türkiye; ^9^ Department of Pediatric Hematology and Oncology, Hospital of Koç University, Istanbul, Türkiye; ^10^ Department of Pediatric, School of Medicine, Koç University, Istanbul, Türkiye; ^11^ Department of Pediatric Hematology and Oncology, Ankara Bilkent City Hospital, Ankara, Türkiye; ^12^ Department of Pediatric Hematology and Oncology, Ankara Yıldırım Beyazıt University, Ankara, Türkiye; ^13^ GENIVA Information Health Services Company, Istanbul, Türkiye; ^14^ Department of Translational Medicine, Institute of Health Sciences, Acıbadem Mehmet Ali Aydınlar University, Istanbul, Türkiye; ^15^ Department of Pediatric Hematology, Samsun Medical Park Hospital, Samsun, Türkiye; ^16^ Department of Pediatric Hematology and Oncology, Faculty of Medicine, Ankara University, Ankara, Türkiye; ^17^ Department of Pediatric Oncology, Faculty of Medicine, Kocaeli University, Izmit, Kocaeli, Türkiye; ^18^ Department of Pediatric Hematology, Faculty of Medicine, Istinye University, Istanbul, Türkiye; ^19^ Department of Pediatric Hematology, Biruni University, Istanbul, Türkiye; ^20^ Department of Pediatric Hematology Oncology, Hatay Training and Research Hospital, Hatay, Türkiye; ^21^ Department of Cancer Genetics, Umraniye Traning and Research Hospital, Istanbul, Türkiye; ^22^ Department of Genome Studies, Institute of Health Sciences, Acıbadem Mehmet Ali Aydınlar University, Istanbul, Türkiye; ^23^ Department of Medical Genetics, School of Medicine, Acıbadem Mehmet Ali Aydınlar University, Istanbul, Türkiye; ^24^ Department of Bioinformatics and Biostatistic, Institute of Health Sciences, Acıbadem Mehmet Ali Aydınlar University, Istanbul, Türkiye; ^25^ Department of Genetics, Istanbul University, Institute of Aziz Sancar Experimental Medicine, Istanbul, Türkiye; ^26^ Department of Clinical Genetics and Genomics, Sahlgrenska University Hospital, Gothenburg, Sweden; ^27^ Department of Laboratory Medicine, Institute of Biomedicine, Sahlgrenska Academy, University of Gothenburg, Gothenburg, Sweden; ^28^ Department of Neurology, Krankenhaus Nordwest, Frankfurt, Germany; ^29^ International Biomedicine and Genome Institute (iBG), Izmir Dokuz Eylül University, Izmir, Türkiye

**Keywords:** germline variants, short-read sequencing, cancer predisposition, childhood leukemia, childhood lymphoma

## Abstract

**Background:**

Leukemia is the most common cancer in children, and 10%–15% of patients with leukemia/lymphoma carry pathogenic germline cancer-predisposing variants. Identifying these variants is critical for understanding the genetic predisposition and optimizing clinical management.

**Methods:**

We performed germline short-read sequencing in 36 individuals from 20 families with suspected leukemia/lymphoma predisposition, including 20 index cases, 9 affected relatives, and 7 unaffected members.

**Results:**

We identified 13 clinically relevant germline variants in known cancer predisposition genes including *TP53, ETV6, MSH6, MLH1,* and *BRCA1*. Notably, we uncovered novel candidate variants in *ATR, TNFRSF9, ETAA1*, and *KSR1*, which was supported by segregation analysis, consanguinity patterns, and secondary malignancy phenotypes. Several index cases exhibited striking familial cancer syndromes involving both hematologic and solid tumors, with progression from ALL to AML or glioma. Deep clinical–genomic correlation enabled reclassification of variants and refined diagnostic and therapeutic decision-making in multiple cases. The patients were referred to genetic counseling for surveillance of carriers and risk assessment for various family members.

**Conclusion:**

These findings emphasize the clinical utility of germline testing in pediatric hematologic cancers by providing novel insights into the predisposition to leukemia/lymphoma and contributing to treatment regimens, donor selection, and diagnostic refinement, particularly in populations with high consanguinity.

## 1 Introduction

Leukemia is characterized by the arrest and clonal proliferation of hematopoietic stem cells in a specific stage of normal hematopoiesis and subsequent accumulation of neoplastic cells in the bone marrow, peripheral blood, and other tissues. Lymphoma is a malignancy caused by a similar process that starts in the lymphatic system. Of the childhood cancers, leukemia is the most common, followed by central nervous system tumors and lymphomas (Zhang et al., 2015). Although the development of leukemia and lymphoma has not yet been fully clarified, the role of germline variants in cancer predisposition genes is becoming clearer (Bloom et al., 2020). The diagnosis of a malignancy necessitates immediate intervention, and the underlying factors, such as a germline variant, occasionally go undetected by treatment centers. However, recent genomic studies have identified germline predispositions in 5%–18% of all childhood cancers. The diagnosis is influenced by differences in study cohorts, study design, and the definition of a positive germline finding (Zhang et al., 2015; Bloom et al., 2020; Tesi et al., 2024; Bakhuizen et al., 2024). Because of decreasing costs and continuous improvements in technology, next-generation sequencing (NGS) can be incorporated into the routine diagnostic work-up for these diseases (Brozou et al., 2022; Byrjalsen et al., 2020; Gröbner et al., 2018).

The incidence rate of childhood cancers in Türkiye is reported as 3.1% (http://iicc.iarc.fr). However, since the registry studies in Türkiye are limited and the NGS-based cancer predisposition evaluation is not covered by the general healthcare system, the expected incidence is much higher. In addition, the general rate of consanguineous marriages in Türkiye has been reported to be as high as 24% (Hacettepe University Institute of Population Studies, et al., 2019; Dündar and Karabulut, 2010). Such consanguinity increases the risk of constitutional mismatch repair deficiency (CMMRD) and CMMRD-like conditions, including Lynch syndrome, while also complicating the analysis of numerous rare homozygous variants. Evaluating these rare homozygous variants is challenging as the Turkish population is underrepresented in major public datasets such as gnomAD.

Multicenter studies initiated by the European Framework Programs in different countries have increased the awareness of germline predisposition factors in malignancies, including leukemias/lymphomas, and our study group was involved in one such study (https://www.cost.eu/actions/CA16223/). Additionally, we started a close collaboration with the Swedish Childhood Cancer Predisposition (ChiCaP) project (Tesi et al., 2024) for further analysis through whole-genome sequencing (WGS).

In this study, we aimed to identify both known and novel gene variants associated with childhood leukemia/lymphoma predisposition in Türkiye. Using short-read sequencing technologies, we studied children with leukemia/lymphoma along with their affected and unaffected family members. This study represents the first systematic analysis of families with high predisposition risk to childhood cancer in Türkiye and adds to the limited literature on germline predisposition to leukemia/lymphoma in the region.

## 2 Materials and methods

### 2.1 Study design and patient cohort

**FIGURE 1:**
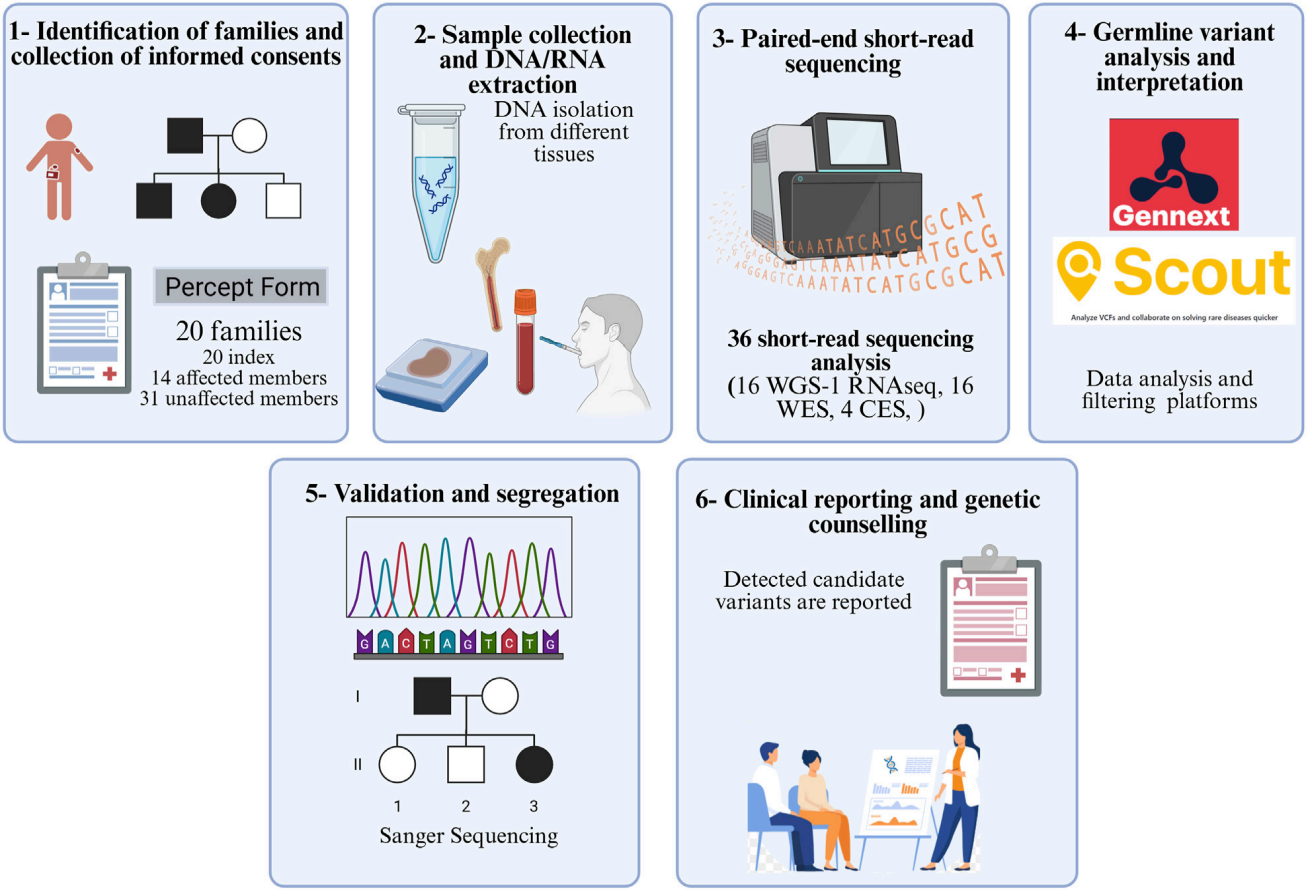
Workflow of the study (WGS: whole-genome sequencing; WES: whole-exome sequencing; CES: clinical exome sequencing).

Between 2019 and 2024, children under the age of 18 years who developed leukemia/lymphoma and exhibited increased risk for cancer predisposition according to Jongmans’ criteria (Jongmans et al., 2016) were included in the study. All participants and/or their legal guardians provided written informed consent. Patients were recruited from eight pediatric hemato-oncology departments across hospitals in Türkiye. Additional clinical information and laboratory investigation results were systematically recorded in an in-house form. The samples and data generated in this study were archived in the Acibadem University Biobank Unit. An overview of the study design is presented in Figure 1. This study was approved by the Acibadem Healthcare Institutions Medical Research Ethics Committee (ATADEK; no. 2017-16/5 and no. 2024-11/496).

### 2.2 Sample collection and DNA extraction

Peripheral blood or bone marrow samples were obtained during remission, and when available, buccal swab samples were collected from the index patients and their family members who were diagnosed with a hematological malignancy in childhood. For segregation analysis, blood samples were collected from the parents and other available family members. Germline DNA from remission blood or bone marrow samples was extracted using the QIAamp/QIAquick DNA extraction and purification kit (QIAGEN, Hilden, Germany). DNA from buccal swabs was extracted using the buccal swab DNA isolation kit (Hibrigen Biotechnology, Kocaeli, Türkiye). Genomic DNA from formalin-fixed paraffin-embedded samples was extracted using the QIAamp DNA FFPE Tissue Kit (QIAGEN, Hilden, Germany).

### 2.3 Paired-end short-read sequencing

Standard paired-end short-read sequencing was performed on germline DNA samples. Whole-exome sequencing (WES) was performed using the NovaSeq 6000 system (Illumina, Inc., San Diego, CA, United States) with the S1 reagent kit v1.5. Clinical exome sequencing (CES), which covers exons and flanking intronic regions of 4,490 clinically relevant genes, was performed using the SOPHiA Clinical Exome Solution v2 kit (SOPHiA Genetics, Lausanne, Switzerland) in Umraniye Training and Research Hospital, Istanbul, Türkiye. Additionally, short-read RNA sequencing (RNA-seq) was performed for a single case (case #13). WGS with 30x coverage was conducted at Science for Life Laboratory (SciLifeLab; Stockholm, Sweden), as previously described (Stranneheim et al., 2021).

### 2.4 Bioinformatics analysis

All sequencing data were subjected to bioinformatic processing, annotated, and filtered for candidate variants using the GenNext platform (https://app.gennext.bio/auth) (https://github.com/GenivaInformatics/gennext-workflows, Geniva, Istanbul, Türkiye). WGS data analysis was performed using the mutation identification pipeline (Stranneheim et al., 2021). Annotated and ranked variants in WGS data were further filtered and interpreted using the visualization tool Scout (https://github.com/Clinical-Genomics/scout). WGS was performed using the hg19 (GRCh37) reference genome, while WES was conducted using the hg38 (GRCh38) reference genome. The WGS data were analyzed using the ChiCaP clinical gene panel, which includes 189 well-established childhood cancer predisposition genes (Tesi et al., 2024). A detailed description of sequencing data analysis is provided in Supplementary File 1 and Supplementary Figures 1–2.

The Turkish Variome dataset was used to evaluate the variant allele frequencies of the candidate variants (Kars et al., 2021). Missense variants of unknown significance (VUS) were evaluated using variant analysis with multiple pathogenicity predictors (VAMPP), as described previously (Ozdemir et al., 2024). VAMPP scores higher than 0.35 indicated moderate evidence for pathogenicity (PP3) of variants (https://vamppscore.com/).

Moreover, we utilized AlphaFold (Jumper et al., 2021) to determine the predicted local distance difference test (pLDDT) scores and spliceAI (Jaganathan et al., 2019) for the splice site variants. All the candidate variants were validated, and segregation analyses were performed by Sanger sequencing for parental DNA samples using standard protocols. Additionally, ΔΔG values were calculated for missense variants with the DynaMut2 tool (https://biosig.lab.uq.edu.au/dynamut2/). A negative ΔΔG value indicates that the mutation is destabilizing, indicating a decrease in stability. In this context, the ΔΔG value was calculated for 15 missense variants.

The variant classifications were finalized with the combined analysis of these findings according to the American College of Medical Genetics (ACMG) (Richards et al., 2015).

## 3 Results

We identified 20 children with leukemia/lymphoma with suspected cancer predisposition and no known genetic diagnosis at the time of inclusion. The study also included 14 family members who were diagnosed with leukemia or any cancer, along with 31 unaffected family members. Five (25%) patients had congenital abnormalities, three children experienced treatment toxicity, six children developed an additional primary tumor alongside their hematological malignancies, and one child had more than two additional primary tumors (Table 1). We performed 16 WGS (four index cases, five members with previous cancer diagnosis, and seven healthy members), 16 WES (12 index cases and four members with previous cancer diagnosis), and four CES.

**TABLE 1:** Clinical features of the cases. The clinical features of the index cases and the indications of enrollment according to Jongmans’ criteria are summarized. The biological sample that was studied and the short-read sequencing methodology are also described. (WES; whole-exome sequencing, WGS; whole-genome sequencing, CES; clinical exome sequencing, RNA Seq; RNA sequencing; p: paternal; m: maternal).

Case Vignette Code	Sex	Primary tumor	Secondary tumor	Age of diagnosis	Age of study enrollment	Syndromic findings	Jongmans’ Criteria	Family history	Biological Samples	Consanguinity	Treatment	Status	Short-read sequencing	Genetic counseling
1	F	pre-B-ALL	AML	15 years (2017)	2019	Fibroadenoma	1-2-5	Affected father, uncle (p), and aunt (p)	Bone marrow and peripheral blood	No	Chemotherapy and bone marrow transplantation	Remission	WES (index)	Yes
2	M	B-ALL	AML	9 years (2021)	2021	Anguli oris hypoplasia, vascular lesions, and thrombocytopenia	1-2-4	Affected brother	Bone marrow	No	Chemotherapy and bone marrow transplantation	Remission	WES (index and affected brother)	Yes
3	M	B-ALL	-	9 years (2019)	2019	Prominent forehead, thick eyebrows, large eyes, large ears, and flat philtrum	1-4	Affected brother and father's uncle	Peripheral blood	No	Chemotherapy	Remission	WES (index and affected brother)	Yes
4	M	Pre-B-ALL	-	3 years (2020)	2020	-	1	Affected father, uncle (p), and grandfather(p)	Peripheral blood	Same village	Chemotherapy	Remission	WES (index and affected father)	Yes
5	F	T-Lymphoma/AML	Astrocytoma	7 years (2019)	2022	Cafe-au-lait spots	1-2-4	-	Bone marrow and buccal swab	Yes	Chemotherapy and bone marrow transplantation	Remission	WES (index)	Yes
6	M	T-ALL/bi-phenotypic leukemia	Low-Grade Glioma	7 years (2021)	2021	-	1-2-3	Affected brother and grandfather (p)	Peripheral blood and swab	Yes	Chemotherapy and bone marrow transplantation	Remission	WES (index)	Yes
7	M	B-ALL	-	12 years (2021)	2022	-	1- 5	Affected mother	Peripheral blood	Unknown	Chemotherapy and bone marrow transplantation	Remission	WES (index)	Yes
8	F	B-ALL	-	2 years (2011)	2021		1	Affected father, uncle (p), and aunt (p)	Peripheral blood	No	Chemotherapy	Remission	WES (index and affected father)	Yes
9	F	B-ALL	-	8 years (2019)	2019		1	Affected mother, grandfather, and uncles (m)	Bone marrow	No	Chemotherapy	Remission	WES (index)	Yes
10	M	B-ALL	-	2 years (2019)	2019		1	Affected mother, grandmother, and father's aunt	Bone marrow	Unknown	Chemotherapy	Remission	WES (index)	Yes
11	F	B-ALL		12 years (2019)	2019	Pectus excavatum	1-2-4-5	Affected mother and father's uncle	Peripheral blood	Yes	Chemotherapy	Remission	WES (index)	Yes
12	M	Hodgkin Lymphoma	-	11 years (2022)	2022	-	1	Affected mother and mother's uncle	Peripheral blood	No	Chemotherapy	Remission	WES (index)	Yes
13	M	Hodgkin lymphoma	-	13 years (2021)	2021	-	1-2	Affected father, brother, and uncle (p)	Peripheral blood and swab	Yes	Chemotherapy and bone marrow transplantation	Remission	CES (index, affected father, and brother); WGS (index, affected father and brother, and unaffected mother); RNAseq (index)	Yes
14	M	pre-B-ALL	Renal Cell Carcinoma	4 years(2019)	2019	-	2	Affected aunt (m)	Peripheral blood	Same village	Chemotherapy	Ex	WES (index); WGS (index, unaffected twin, father, mother, and brother)	Yes
15	F	B-ALL	-	4 years (2008)	2021	-	1	Affected aunt (m) and grandmother (m)	Peripheral blood	No	Chemotherapy	Remission	WES (index); WGS (index, unaffected father and mother, and affected aunt and grandmother)	Yes
16	F	B-ALL	-	2 years (2018)	2019	Sparse eyebrow	1-4	Affected cousin	Peripheral blood	Yes	Chemotherapy	Remission	WES (index and affected cousin); WGS (index and affected cousin)	Yes
17	F	Non-Hodgkin’s lymphoma	-	6 years (2017)	2021	-	1	Affected grandmother (p) and grandmother's sister(p)	Peripheral blood	No	Chemotherapy	Remission	CES (index)	Yes
18	F	B-ALL	-	5 years (2019)	2021	-	1	Affected grandmother (m) and grandfather (m)	Peripheral blood	No	Chemotherapy	Remission	CES (index)	Yes
19	F	T-ALL	T-ALL	4 years (2010)	2021	-	1	Affected aunts (p)	Peripheral blood	No	Chemotherapy	Remission	CES (index)	Yes
20	F	B-ALL	-	6 years (2015)	2021	-	1	Affected aunt (m) and grandfather (p)	Peripheral blood	No	Chemotherapy	Remission	CES (index)	Yes

### 3.1 Baseline characteristics

We analyzed 36 individuals, including 20 index cases (diagnosis age median: 8 years, female/male: 11/9) and nine affected and seven unaffected family members. Seven out of 20 families (35%) reported consanguinity. Of the index cases, 14 were diagnosed with B-ALL (acute lymphoblastic leukemia), two with T-ALL, one with T-lymphoblastic lymphoma, two were Hodgkin’s lymphoma cases, and one was a non-Hodgkin’s lymphoma case (Table 1; Supplementary File 2).

### 3.2 Frequency and spectrum of germline variants

**FIGURE 2:**
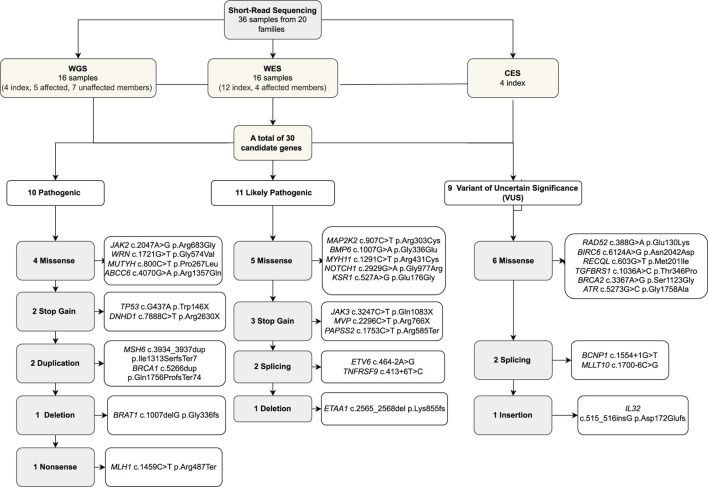
Cohort of the study.

**FIGURE 3:**
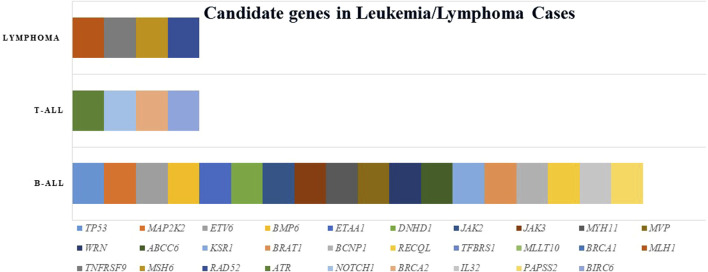
Candidate genes in leukemia and lymphoma.

Twenty index patients presented 30 candidate variants (Figure 2; Table 2). The candidate genes observed in the B-ALL cases were *ABCC6, BCNP1, BMP6, BRAT1, BRCA1, DNHD, ETAA1, ETV6, JAK2, JAK3, KSR1, MAP2K2, MLLT10, MUTYH, MYH11, MVP, PAPSS2, RAD52, RECQL, TGFBRS1, TP53,* and *WRN*. The candidate genes observed in the T-ALL cases were *ATR, BRCA2, BIRC6,* and *NOTCH1*, and those observed in lymphoma cases were *MLH1, MSH6, RAD52,* and *TNFRSF9* (Figure 3).

**TABLE 2:** Candidate gene variants. A total of 30 candidate variants were determined in 20 index cases. (Chr; chromosome, Het; heterozygous, Hom; homozygous).

Case Vignette Code	Chr	Position	Ref_Base	Alt_Base	Gene	Transcript	Sequence_Ontology	cDNA_change	Protein_Change	rs ID	ClinVar ID	CADD_Exome_phred (GRCH38-V1.7)	VAMPP score	AlphaMissense	GERP++	Turkish Variome	Turkish # of het	Turkish # of hom	GnomAD_allel_frequency	Zygosity	ACMG criteria
1	17	7675175	C	T	*TP53*	NM_000546	StopGain	c.437G>A	p.Trp146X	rs1206165503	634785	35	-	-	5.3	NA	NA	NA	0.000001	Het	Pathogenic (PVS1, PP5, and PM2)
2	12	11869422	A	G	*ETV6*	NM_001987	Splicing	c.464-2A>G	-	-	-	28.4	-	-	5.38	NA	NA	NA	NA	Het	Likely Pathogenic (PVS1, PM2, and PP1)
3	19	4099213	G	A	*MAP2K2*	NM_030662.4	Missense	c.907C>T	p.Arg303Cys	rs770521279	1334259	27.7	0.156361	0.22	4.4	0.0006228	5	NA	0.000004	Hom	Likely Pathogenic (PP3, PM2, and PP1)
3	6	7862301	G	A	*BMP6*	NM_001718.6	Missense	c.1007G>A	p.Gly336Glu	rs1463761372	-	32	0.303169	0.62	5.8	0.0001236	1	NA	NA	Het	Likely Pathogenic (PM2, PP3, and PP1)
4	2	67405247	AAG	-	*ETAA1*	NM_019002	Frameshift deletion	c.2565_2568del	p.Lys855fs	rs759474663	-	33	-	-	-	NA	NA	NA	0.000001	Het	Likely Pathogenic (PM2, PP1, and PP4)
4	11	6557183	C	T	*DNHD1*	NM_144666	StopGain	c.7888C>T	p.Arg2630X	rs1017065128	-	36	-	-	3.43	0.001116	9	NA	NA	Het	Pathogenic (PVS1, PM2, and PP3)
5	2	47806583	-	AGTT	*MSH6*	NM_000179.3	Duplication	c.3934_3937dup	p.Ile1313SerfsTer7	rs760190301	418610	-	-	-	-	NA	NA	NA	0	Hom	Pathogenic (PVS1, PM2, and PP5)
6	3	142503377	C	G	*ATR*	NM_001184.4	Missense	c.5273G>C	p.Gly1758Ala	-	-	27.2	0.310000	0.86	5.34	NA	NA	NA	NA	Hom	Variant of unknown significance (PM1, PM2, and PP1)
6	9	136509773	C	T	*NOTCH1*	NM_017617.5	Missense	c.2929G>A	p.Gly977Arg	rs1240954845	-	27.2	-	0.88	4.5	NA	NA	NA	NA	Het	Likely Pathogenic (PP2, PM2, and PP3)
6	2	32468780	A	G	*BIRC6*	NM_016252.4	Missense	c.6124A>G	p.Asn2042Asp	rs758886165	-	24.2	-	0.19	5.69	NA	NA	NA	0.000004	Het	Variant of unknown significance (PM2 and PP2)
7	8	31090834	G	T	*WRN*	NM_000553.6	Missense	c.1721G>T	p.Gly574Val	-	933329	35	0.304208	0.21	4.74	NA	NA	NA	NA	Het	Pathogenic (PP3, PP5, PM1, and PM2)
8	16	15759686	G	A	*MYH11*	NM_002474.3	Missense	c.1291C>T	p.Arg431Cys	-	-	33	0.389103	0.60	5.75	NA	NA	NA	NA	Het	Likely Pathogenic (PM1,PM2, PP1,PP2, and PP3)
9	9	5078360	A	G	*JAK2*	NM_004972.4	Missense	c.2047A>G	p.Arg683Gly	rs1057519721	375951	33	0.300726	0.50	4.55	NA	NA	NA	NA	Het	Pathogenic (PM5, PM1, PP3, PM2, and PP5)
10	19	17826871	G	A	*JAK3*	NM_000215.4	StopGain	c.3247C>T	p.Gln1083X	-	-	38	-	-	-	NA	NA	NA	NA	Het	Likely Pathogenic (PVS1 and PM2)
11	16	29847227	C	T	*MVP*	NM_005115.5	StopGain	c.2296C>T	p.Arg766X	rs764310278	-	37	-	-	2.78	NA	NA	NA	0.00001	Het	Likely Pathogenic (PM2, PP2, and PP3)
11	16	3069303	-	G	*IL32*	NM_001376923.1	Frameshift insertion	c.515_516insG	p.Asp172Glufs*	rs398100042	-	-	-	-	-	NA	NA	NA	NA	Het	Variant of unknown significance (PM2)
12	12	927224	C	T	*RAD52*	NM_134424.4	Missense	c.388G>A	p.Glu130Lys	-	-	32	-	0.96	4.18	0.001112	9	NA	NA	Het	Variant of unknown significance (PM2)
13	1	7937684	A	G	*TNFRSF9*	NM_001561.6	Splicing	c.413+6T>C	-	-		20.8	-	-	-	NA	NA	NA	NA	Hom	Likely Pathogenic (PP3, PM2, and PP1)
14	19	17654441	G	T	*BCNP1*	NM_001321827.2	Splicing	c.1554+1G>T	-	rs755232157	-	30	-	-	-	0.0003708	3	NA	0.00008	Het	Variant of unknown significance (PM2 and PP1)
14	12	21483473	C	A	*RECQL*	NM_002907.4	Missense	c.603G>T	p.Met201Ile	-	1751237	33	0.214766	0.37	4.67	NA	NA	NA	NA	Het	Variant of unknown significance (PM2)
14	9	99144794	A	C	*TGFBRS1*	NM_004612.4	Missense	c.1036A>C	p.Thr346Pro	rs1827740361	-	27.8	0.329917	0.55	4.73	NA	NA	NA	0.000006	Het	Variant of unknown significance (PM1 and PM2)
14	10	87745863	C	T	*PAPSS2*	NM_001015880.2	StopGain	c.1753C>T	p.Arg585Ter	rs1853931024	-	47	0.419909	-	5	NA	NA	NA	0.000006	Het	Likely Pathogenic (PVS1 and PM2)
14	10	21977414	C	G	*MLLT10*	NM_004641.4	Splicing	c.1700-6C>G	-	-	-	22.2	-	-	-	NA	NA	NA	NA	Het	Variant of unknown significance (PM2)
15	17	27583063	A	G	*KSR1*	NM_014238.2	Missense	c.527A>G	p.Glu176Gly	-	-	26.1	-	0.10	5.6	NA	NA	NA	NA	Het	Likely Pathogenic (PM2 and PP1)
15	7	2542128	C	-	*BRAT1*	NM_001350626	Frameshift deletion	c.1007delG	p.Gly336fs	rs2128393384	1453640	-	-	-	-	NA	NA	NA	NA	Het	Pathogenic (PVS1, PP5, and PM2)
16	1	45332215	C	T	*MUTYH	NM_001048174.2	Missense	c.800C>T	p.Pro267Leu	rs374950566	185242	27.9	0.457363	0.55	5.5	0.00297	22	1	0.00001	Het	Pathogenic (PP3, PP5, PM2, and PP2)
17	3	37028833	C	T	*MLH1*	NM_000249.4	Nonsense	c.1459C>T	p.Arg487Ter	rs63749795	89744	35	-	-	-	NA	NA	NA	0.00009	Het	Pathogenic (PVS1, PP5, and PM2)
18	17	43057063	-	G	*BRCA1*	NM_007294.4	Frameshift duplication	c.5266dup	p.Gln1756ProfsTer74	rs80357906	17677	-	-	-	-	NA	NA	NA	0.00005	Het	Pathogenic (PVS1, PS3, PP5, and PM2)
19	13	32337722	A	G	*BRCA2	NM_000059.4	Missense	c.3367A>G	p.Ser1123Gly	rs80358581	51455	25.7	-	0.19	5.3	NA	NA	NA	0.000001	Het	Variant of unknown significance (PP3, PM2, and BP1)
20	16	16154766	G	A	*ABCC6	NM_001171.6	Missense	c.4070G>A	p.Arg1357Gln	rs201275608	1359254	29.8	0.365574	0.40	3.63	0.0002475	2	NA	0.00007	Het	Pathogenic (PM5, PM1, PM2, and PP3)

AlphaFold provided pLDDT scores for 28 variants (*DNHD1* and *BIRC6* were not available), and 13 of these (*TP53, BMP6, JAK2/3, MYH11, MVP, RAD52, WRN, MUTYH, TGFBRS1, RECQL, ABCC6,* and *PAPSS2)* had very high scores. To assess the impact on protein dynamics and stability, we analyzed the stabilization status using DynaMut2 (https://biosig.lab.uq.edu.au/dynamut2/). Eleven missense variants were found to be destabilizing *(BMP6, JAK2, MYH11, ATR, NOTCH, RAD52, WRN, MUTYH, RECQL, BRCA2,* and *ABCC6*) (Supplementary File 3).

Additionally, 15 missense variants were also evaluated by the AlphaMissense tool, and eight of them had a score of >0.5 (Supplementary File 1). Two splice-site variants (*ETV6* and *BCNP1*) were at the essential splice site (Supplementary file 2). The spliceAI algorithm yielded higher than 0.5 scores of *TNFRSF9* c.413 + 6T>C variant in case #13, and the RNA sequencing of the index revealed an alternative splice site between exons 5 and 6 (Supplementary File 2.15–2.16).

Seven candidate variants (*MAP2K2, BMP6, DNHD1, RAD52, MUTYH, RECQL,* and *ABCC6)* presented allele frequencies <0.001 in Turkish Variome (Table 2).

Altogether, based on all the provided evidence, 10 variants were classified as pathogenic (P), 11 were likely pathogenic (LP), and nine were VUS. Eleven out of the 16 missense variants were suitable for VAMPP score evaluation, and four of them (*MYH11, MUTYH, TGFBRS1,* and *ABCC6*) showed scores >0.35 but did not change the variant classification of VUS (Table 2; Figure 2).

### 3.3 Variants causing cancer predisposition

In the 13 index cases, we hypothesized that the candidate variants caused cancer predisposition. The detailed information, pedigrees, and genotypes of the cases and variants are provided as case vignettes in Supplementary File 2.

Index case #1 (Supplementary File 2.1) was diagnosed with B-ALL, and we determined a germline pathogenic stop-gain variant in *TP53* c.437G>A p.Trp146Ter. The patient successfully completed treatment for B-ALL in 2019; however, she was diagnosed with acute myeloid leukemia (AML) in 2021. In index case #2 (Supplementary File 2.2), who had B-ALL, we found a maternally inherited LP *ETV6* c.464-2A>G variant. It was also detected in the patient’s affected brother, who died of AML after a T-ALL diagnosis.

The LP *MAP2K2* c.907C>T p.R303C and LP *BMP6* c.1007G>A p.Gly336Glu variants were detected in B-ALL index case #3 (Supplementary File 2.4) and his brother with Hodgkin’s lymphoma. In addition, our case presented elevated iron levels. Their parents were first-degree cousins and were heterozygous for both variants.

In index case #4 (Supplementary File 2.5), the diagnosis was of B-ALL. The patient’s father and one paternal uncle had been treated for Hodgkin’s lymphoma; they suffered relapse after 10 and 20 years, respectively, after the first diagnosis, and additional thyroid papillary carcinoma and synovial sarcoma were diagnosed in the father. The patient’s parents were consanguineous, and all the affected family members were positive for the likely pathogenic variant *ETAA1* c.2565_2568del p.K855fs and the pathogenic variant *DNHD1* c.7888C>T p.R2630X, whereas the mother and the siblings, who were unaffected, were negative.

Index case #5 (Supplementary File 2.6) was found to be homozygous for the pathogenic variant *MSH6* c.3934_3937dup p.Ile1313SerfsTer7. T-ALL was diagnosed when the patient was 12 years old, and 3 years later, primary AML and astrocytoma were diagnosed. Their parents were first-degree cousins, and both were heterozygous for the candidate variant.

We hypothesized that the VUS *ATR* c.5273G>C p.Gly1758Ala was the causative factor in case #6 (Supplementary File 2.7), who had T-ALL. Both the patient and his brother, who also had T-ALL and died due to treatment toxicity, were homozygous for this variant. The buccal swab analysis confirmed the homozygous status of the variant in the index case. A year later, the index case developed a low-grade glioma. The parents were first-degree cousins, and both were heterozygous for this variant. The patient was also heterozygous for the LP *NOTCH1* c.2929G>A p.Gly977Arg and homozygous VUS *BIRC6* c.6124A>G p.Asn2042Asp variants. *NOTCH1* was also heterozygous in the affected brother and the father, but the mother was wild-type (WT), whereas *BIRC6* was heterozygous in the mother and homozygous in the index. The affected brother’s DNA material was insufficient to check the *BIRC6* variant.

In index case #7 (B-ALL, Supplementary File 2.8) and in the mother (Hodgkin’s lymphoma), the pathogenic variant present was *WRN* c.1721G>T p. Gly574Val was detected. His parents were second-degree cousins. B-ALL was diagnosed in index case #8; AML had been diagnosed in her father. They were positive for the LP *MYH11* c.1291C>T p.Arg431Cys variant. The family reported members on the paternal side who had died of lymphoma and osteosarcoma.

We found heterozygosity for the LP *MVP* c.2296C>T p.Arg766X and *IL32* c.515_516insG p.Asp172Glufs* variants in both the index case #11 (Supplementary File 2.12) and her mother, in whom colon cancer had been diagnosed at an early age. The parents were consanguineous, and bladder cancer had been diagnosed in the father’s paternal uncle.

Hodgkin’s lymphoma was diagnosed not only in index case #13 (Supplementary File 2.14) but also in his brother, his father, and two of his paternal uncles. WGS revealed homozygosity for the likely pathogenic variant *TNFRSF9* c.413 + 6T>C in all the affected cases, and the variant was comprehensively evaluated by RNA sequencing for possible alternative transcripts. The variant was confirmed in the buccal swab of the index patient. The parents were consanguineous, and the mother was heterozygous for the variant.

WES revealed heterozygosity for the pathogenic variant *BRAT1* c.1007delGG336fs in index case #15 (Supplementary File 2.19), who had B-ALL. The mother was also heterozygous for the same variant, whereas the maternal aunt, who had had ovarian cancer, and the maternal grandmother, who had breast cancer, were WT. Subsequent WGS analysis revealed the LP *KSR1* c.527A>G p.Glu176Gly in all affected family members, and this variant was, therefore, considered causative. Another clinically relevant variant was pathogenic *MLH1* c.1459C>T p.Arg487Ter. We determined that index case #17(Supplementary File 2.21), who had non-Hodgkin’s lymphoma, was heterozygous for this variant, as was her unaffected father. The father’s mother, maternal aunt, and maternal grandmother had died of breast cancer.

We also determined the heterozygous pathogenic *BRCA1* c.5266dup p.Gln1756ProfsTer74 variant in both the index case #18 (Supplementary File 2.22) and her mother by CES analysis. The maternal grandmother had died of breast cancer at the age of 45; the mother’s father was WT, which confirms that the variant was maternally inherited by the patient’s mother.

For case #9, case #10, case #12, and case #19 (Supplementary File 2.10, 2.11, 2.13, and 2.23, respectively), the analysis revealed pathogenic *JAK2*, likely pathogenic *JAK3, VUS RAD52*, *and VUS BRCA2* variants, respectively, but only the index case’s samples were available, and further analysis could not be performed within the families. The evidence was insufficient to support the potential causative effect of these variants, and they remained unsolved (Table 2). Case #14 was diagnosed with B-ALL, and soon after the initial diagnosis, brain metastasis occurred, and the patient died following the development of renal cell carcinoma. The WGS analysis of the peripheral blood sample revealed candidate variants in *BCNP1, RECQL, TGFBRS1, PAPSS2,* and *MLLT10*, but none showed strong enough evidence to be considered causative. Likewise, in case #16, an incidental pathogenic heterozygous *MUTYH* variant was identified. However, pathogenicity in *MUTYH* is typically associated with bi-allelic variants, particularly in the context of *MUTYH*-associated polyposis. This variant was heterozygous and not present in the affected cousin. Furthermore, the family had no history of gastrointestinal symptoms suggestive of polyposis. Therefore, we did not consider this variant to be related to leukemia predisposition. Although we detected candidate variants, we found no strong evidence for variants detected in cases #14, #16 (Supplementary File 2.20), and #20 (Supplementary File 2.24), and they remained unsolved.

### 3.4 Clinical utility of germline findings

For the clinical utility of these results, we evaluated the genetic findings with the patient’s primary hematologist/oncologist. For instance, in case #1, the *TP53* variant changed the diagnosis to Li–Fraumeni syndrome, and the family was referred to a clinical geneticist for counseling and surveillance plan. In case #5, the homozygous *MSH6* variant changed the diagnosis from neurofibromatosis to constitutional mismatch repair deficiency (CMMRD), and the family was referred to a clinical geneticist for counseling and surveillance plan for the risk of Lynch syndrome. Our findings also led to a change in the treatment or donor candidate. In cases #2, #3, #5, #6, and #7, the unaffected donor siblings were also found to be carriers of the candidate genes, and the transplant planning was changed to be performed either from the WT siblings or unrelated donors.

Genetic counseling was provided by clinical geneticists at the referral centers, in accordance with the NCCN Guidelines for genetic/familial high-risk assessment (https://www.nccn.org/guidelines/category_1). For index cases with established cancer predisposition syndromes—such as Li–Fraumeni syndrome (*TP53*) or constitutional mismatch repair deficiency (CMMRD, *MSH6*)—counseling included a structured discussion of the associated cancer risks, current clinical guidelines, and established surveillance protocols (e.g., whole-body MRI for Li–Fraumeni and colonoscopy for CMMRD). Families were offered cascade testing and referred to relevant specialty clinics, including adult genetics services, to ensure long-term coordination of care.

For variants of uncertain significance or novel candidate variants without established disease associations or management protocols, clinical geneticists focused on transparent communication regarding the current level of evidence, limitations in interpretation, and potential—but not definitive—implications for cancer risk. These sessions emphasized the importance of regular phenotypic monitoring and re-evaluation as new data become available. Surveillance recommendations in these cases were tailored based on the family history, clinical phenotype, and expert consensus. To support clinical integration of the genetic results, all cases with potential diagnostic or therapeutic relevance were presented at multidisciplinary tumor boards, where available. These boards typically included pediatric oncologists, hematologists, pathologists, and clinical geneticists. Discussions focused on treatment implications, variant reinterpretation, and potential surveillance strategies for both the index cases and at-risk relatives. This collaborative setting enabled shared decision-making, particularly in complex or uncertain scenarios.

In centers lacking access to a clinical geneticist, the patient’s primary physician assumed the responsibility of conveying results and implementing follow-up care, supported by structured reports and consultation with the central study team. Families with asymptomatic carriers were counseled on possible future risks and the need for longitudinal follow-up, while psychosocial concerns were addressed through individualized discussions.

Overall, the genetic counseling process—supported by multidisciplinary input—was critical in translating germline findings into personalized clinical action, guiding diagnosis, treatment, and long-term risk management for families affected by childhood hematologic malignancies.

### 3.5 Use of NetworkAnalyst for leukemia/lymphoma predisposition

**FIGURE 4:**
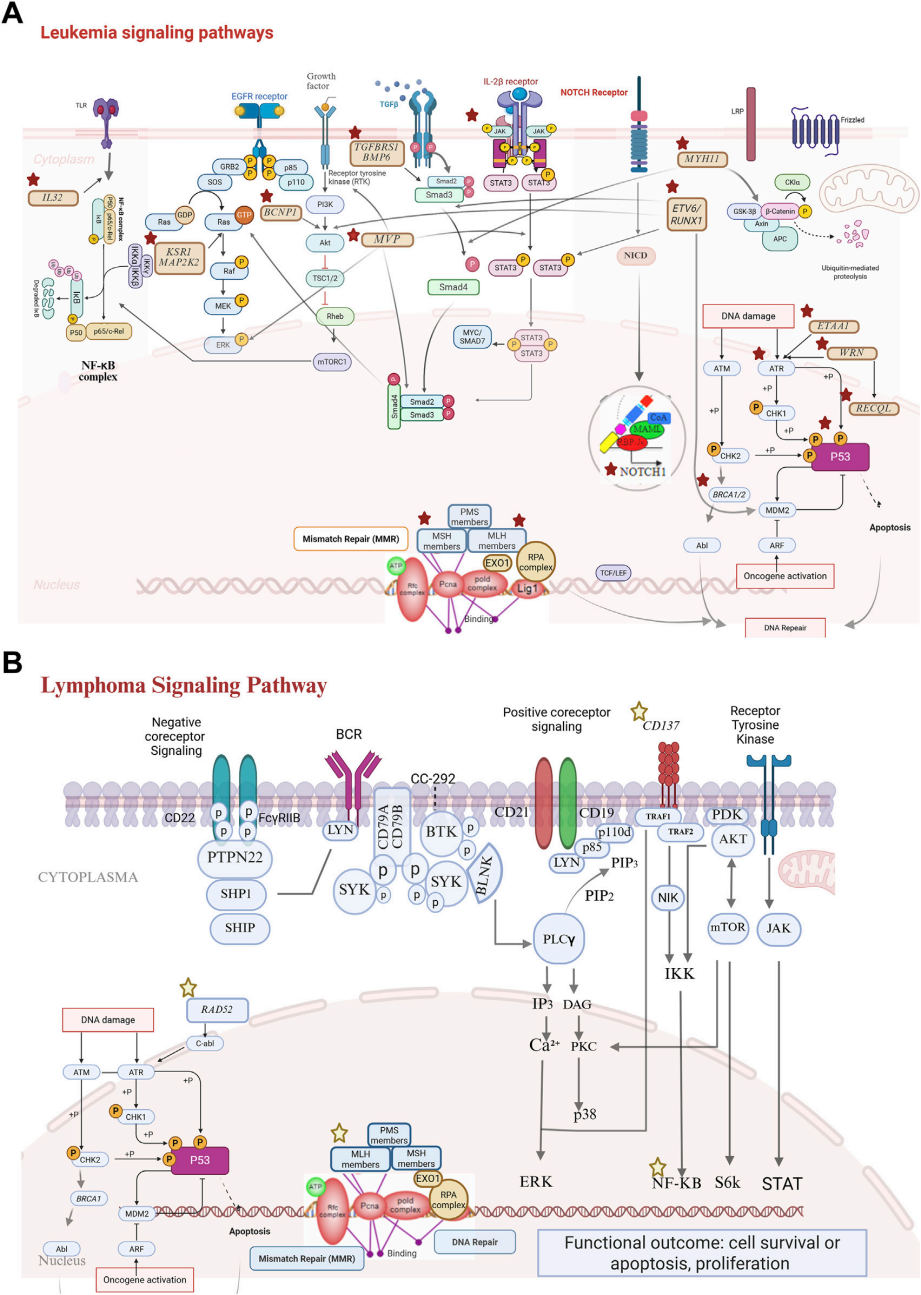
Signaling pathways of candidate genes (stars: genes identified as potential candidates in leukemia/lymphoma cases). **(A)** Leukemia signaling pathways: (cytoplasmic pathways; NF-κB pathway: activation through *IL32* signaling leads to transcriptional regulation via the NF-κB complex. MAPK pathway: initiated through receptor tyrosine kinases, including EGFR, and mediated by KSR1 and *MAP2K2*, driving cell proliferation. PI3K/AKT/mTORC1 pathway: a critical survival and growth axis influenced by *BCNP1* and TGFβ signaling. NOTCH and IL-2β signaling; NOTCH1: NICD (notch intracellular domain) signaling contributes to transcriptional regulation associated with leukemia progression. STAT3 activation: IL-2β receptor engagement results in STAT3 phosphorylation, which impacts the transcriptional activity. DNA repair mechanisms; mismatch repair (MMR): key components such as *PMS, MLH, MSH*, and *EXO1* ensure genomic stability. DNA damage response (DDR): genes such as *WRN, RECQL,* and *BRCA1/2* coordinate repair and apoptotic signaling via the *ATM* and *ATR* pathways. β-catenin pathway: dysregulation of WNT signaling (via GSK-3β inhibition) contributes to leukemic transformation. Tumor suppressor pathways; *P53*: central to apoptosis and cell cycle arrest and frequently inactivated in leukemias. *ETV6:* common in childhood leukemia and drives oncogenic processes.). **(B)** Lymphoma signaling pathways: NF-κB signaling via *TNFRSF9*: TNFRSF9 activation recruits adapter proteins (e.g., TRAF molecules), leading to the phosphorylation and degradation of IκB. This allows the NF-κB complex to translocate to the nucleus, where it regulates genes involved in inflammation, survival, and proliferation. Dysregulation of this pathway is a hallmark of many lymphomas. DNA repair pathways; *RAD52:* critical for homologous recombination repair and for addressing DNA double-strand breaks that arise during replication or due to genotoxic stress. *MSH6* and *MLH1:* ensure replication fidelity by correcting mismatched base pairs. Loss of MMR function increases mutation rates and contributes to lymphomagenesis.

The STRING interaction network within NetworkAnalyst revealed that 26 genes were interconnected either directly or indirectly. Separate network analyses were conducted for all candidate and causative genes (Supplementary Figure 3a, b), as well as for genes associated specifically with leukemia and lymphoma (Supplementary Figure 3c,d). *TP53* was both the central hub and the most connected gene in the network. In the leukemia-specific network, *TP53, NOTCH1, JAK2,* and *BRCA1/2* were found to be key hub genes; in the lymphoma-specific network, *MLH1* was identified as a key hub gene. Of the 18 causative genes, nine *(TP53, ATR, ETAA1, BRCA1, BRCA2, BRAT1, WRN, MLH1,* and *MSH6*) were identified as being responsible for DNA repair and damage response (Figures 4a,b).

## 4 Discussion

Leukemia and lymphoma are the most common childhood hematological malignancies. Approximately 5% of childhood hematological malignancies arise from germline cancer-predisposing gene variants (Zhang et al., 2015; Bakhuizen et al., 2024; Brady et al., 2022; Salmoiraghi et al., 2016) Many cancer-predisposing genes and their association with syndromes or isolated tumors have been described in the literature (Rahman, 2014; Li et al., 2021). Despite these advancements, awareness of genetic predisposition to cancer remains low.

Various guidelines (Baliakas et al., 2019; Goudie et al., 2021) and tools are utilized to assess the childhood predisposition to cancer (Maese et al., 2024; Schlegelberger et al., 2021; Lazic et al., 2023). For the patient selection, we utilized the Jongmans’ criteria (Jongmans et al., 2016) due to its clarity and applicability in clinical settings, particularly in centers where cancer or medical geneticists are not available. We enrolled cases presenting at least one of these following signs: cancer affecting multiple family members across three generations, patients developing multiple primary tumors, bilateral cancer in paired organs, cancer appearing earlier than expected, or patients showing severe toxicity to cancer treatment.

We found strong evidence of the clinical correlation between the variants and cancer predisposition in 13 out of 20 cases. A germline stop-gain *TP53* variant was identified in a B-ALL patient, a mutation known to cause Li–Fraumeni syndrome and recently associated with leukemia (Qian et al., 2018). In our case, we could not obtain samples from the affected family members form the paternal side, but the mother was WT, and we assumed paternal inheritance. Similarly, *ETV6* is one of the most well-known leukemia-predisposing genes. Germline variants are found in the *ETV6* gene and comprehensively described the clinical features in children with ALL who carry these risk variants (Moriyama et al., 2015). We determined the variant at the essential splice site of the *ETV6* gene in our case #2 and his brother, who died during the study. In addition to B-ALL, the patient exhibited syndromic features and thrombocytopenia. The germline *ETV6* variant has been described previously in leukemia/lymphoma cases in a family with thrombocytopenia (Rampersaud et al., 2019). We showed that the variant was maternally inherited. *ETV6* germline variants show reduced penetrance, which may explain why the carrier mother was unaffected (Rampersaud et al., 2019).

Case #3, who had B-ALL, had variants of both the *MAP2K2* and *BMP6* genes, which were also detected in his brother, who had Hodgkin’s lymphoma. *MAP2K2* plays an important role in the RAS signaling pathway, and germline variants in this pathway lead to RASopathies (Tidyman and Rauen, 2016). The activation of the MAPK pathway promotes cell proliferation and survival, and *MAP2K2* is also considered a cancer-predisposing gene, as in Noonan syndrome (Alba-Pavón et al., 2023; Cavé et al., 2016). The additional findings of mild facial dysmorphism may correspond to the effect of the *MAP2K2* homozygous variant. The *MAP2K2* variants were detected in primary central nervous system lymphoma (Fukumura et al., 2016). Among our study cohort, two siblings, one with syndromic leukemia (case #3) and one with Hodgkin’s lymphoma, were homozygous for the *MAP2K2* variant. We also identified a second variant at the pro-peptide domain of *BMP6*, which is necessary for proper folding of the protein and maturation. The variations in this domain have been reported to show a dominant negative effect and are related to iron overload (Daher et al., 2016). Case #3 also exhibited elevated iron levels; however, this might also have been a result of transfusions. NetworkAnalyst revealed *MAP2K2* as a central hub, and it was connected to *TP53* via other MAPK pathway members, which strengthens *MAP2K2* as a predisposing factor.

In case #4 (B-ALL), along with his father and paternal uncle who had Hodgkin’s lymphoma, we detected variants of both *ETAA1* and *DNHD1* genes. To date, *ETAA1* has been reported to cause predisposition to pancreatic cancer and nonpolyposis colorectal cancer. *ETAA1* is a key activator of the ATR-dependent DNA damage response. It directly binds to RPA-coated single-stranded DNA at the stalled replication forks and activates ATR-CHK1 signaling. Germline or somatic mutations in ETAA1 may impair genome integrity and replication stress response, increasing susceptibility to tumorigenesis, particularly in rapidly proliferating tissues such as hematopoietic cells (Yu et al., 2018; Childs et al., 2015). However, the COSMIC database includes variants of *ETAA1* in lymphoma cell lines. The *ETAA1* gene may be a novel predisposition gene that emerges in hematologic cancers in both children and adults. Somatic variants of both candidate genes have been associated with soft-tissue sarcomas and lymphomas in the St. Jude database (https://pecan.stjude.cloud/). Thyroid papillary carcinoma and synovial sarcoma developed in the father, both of which could be related to the germline *DNHD1* variant. *DNHD1* encodes a protein that belongs to the dynein family, which is involved in intracellular transport and, potentially, centrosome stability. Although not previously linked to hematologic malignancies directly, somatic variants of *DNHD1* have been reported in soft-tissue sarcomas and lymphomas in the St Jude database (https://pecan.stjude.cloud/variants/proteinpaint?gene=DNHD1). Aberrant intracellular transport and spindle checkpoint dysregulation may contribute to chromosomal instability, thus indirectly promoting malignant transformation in hematopoietic cells.

Case #5, with cafe-au-lait–like spots on the skin, received a diagnosis of T-ALL at another center and was found negative for the *NF1* gene. Subsequent WES at our institution revealed a homozygous *MSH6* variant. *MSH6* is a member of the DNA mismatch repair system, and its somatic variations have been related to various tumors (Ercan et al., 2024). Similarly, the germline *MSH6* variants are related to Lynch syndrome and CMMRD. T-ALL was diagnosed in case #5, followed by the development of AML and astrocytoma. Consanguinity increases the incidence of CMMRD, and hematologic malignancies are detected frequently in such families (Ercan et al., 2024; Ripperger and Schlegelberger, 2016). The parents were related, and all siblings, except one, were heterozygous for the same mutation; one sister was homozygous. The family was informed about the risk of Lynch syndrome, and surveillance was suggested. In addition, the homozygous sister, who was 4 years old at the time of the study, was referred to related clinics for close monitoring for CMMRD.

We determined a homozygous VUS *ATR* variant, together with the paternally inherited *NOTCH1* and maternally inherited *BIRC6* variants, in case #6, who had T-ALL. This patient’s older brother had had T-ALL and had died of treatment-related toxicity at the age of 5 years. Homozygous *ATR* gene variants are also observed in patients with Seckel syndrome. AML development was reported in a patient with Seckel syndrome who died of severe treatment-related toxicity (Hayani et al., 1994). In our patient, deep phenotyping revealed no dysmorphic features consistent with Seckel syndrome. The amino acid change appeared to have a mild effect; this may explain the non-syndromic but cancer-predisposing effect of the variant. In addition, our patient exhibited loss of chromosome 5q, as reported previously (Hayani et al., 1994). *ATR* plays a role in the DNA damage sensor; therefore, chromosomal instability is expected in the affected patients. *ATR* is a central kinase in the DNA damage response pathway, and it is especially activated by replication stress. It phosphorylates multiple targets, including CHK1, to halt the cell cycle and repair damaged DNA. Miao et al. showed that *NOTCH1* restores the cell cycle deficiency in *BRCA1*-mutated triple-negative breast cancer through ATR/CHK1 signaling (Miao et al., 2020). In our case, both genes were affected, and further studies are needed to investigate the combined effects of *NOTCH1* and *ATR* variants. Because the father was WT for *BIRC6*, the patient’s homozygous status must have resulted from a novel second mutation and led to astrocytoma development (Chen et al., 1999).

We performed WGS for four families whose WES/CES panels were negative, and we were able to define a possible predisposing factor in two cases. Samples from case #13, the affected brother, and the affected father were studied with CES, and no candidate variant could be determined. Later, WGS revealed a novel *TNFRSF9* variant. The *TNFRSF9* gene encodes the cell surface receptor CD137, which contributes to the proliferation, survival, and development of T-cells (Shen et al., 2023). We detected homozygosity for the variant of this gene in this Hodgkin’s lymphoma family; all affected family members were homozygous for the mutation. The constitutive expression of the CD137 ligand has been shown in the serum of patients with B-cell lymphomas (Souza-Fonseca-Guimaraes et al., 2016), which may explain the contribution of this variant to cancer predisposition. Moreover, homozygosity for variants of this gene was shown to lead to immunodeficiency with lymphoproliferation, but Shen et al. described wide clinical heterogeneity, including differences in age at onset and abnormalities ranging from severe to mild (Shen et al., 2023). Our patients did not present any immunodeficiency yet, which may develop later in life.

In case #15, WES revealed a heterozygous *BRAT1* variant both in the patient and in the unaffected mother. However, the maternal grandmother with breast cancer and the maternal aunt with ovarian cancer were WT for *BRAT1*. Subsequent WGS revealed a novel *KSR1* variant in all the affected cases. It falls within the N-terminal regulatory domain of the KSR1 protein, which plays a role in scaffold-mediated regulation of the MAPK/ERK signaling pathway. This domain is essential for mediating protein–protein interactions with RAF and MEK, which is crucial for transmitting RAS signaling. Variants in this region may impair MAPK cascade regulation, contributing to oncogenic processes including leukemogenesis. *KSR1* consists of 22 exons, and translation begins at exon 4 (Liu et al., 2023), where our variant is also located. Disruption of this region has been reported to inhibit protein expression, which may also be occurring in our patients and should be checked by further studies.

Both leukemia and lymphoma are disorders of hematopoietic cells, and they share common molecular pathways. However, their organs of origin and the affected cells differ. Members of the DNA repair or JAK/STAT pathways are deregulated in both conditions (Fernández et al., 2023; Liang et al., 2024), whereas NFKB is prominently involved in lymphoma development (Grondona et al., 2018), and *TP53* or MAPK/RAS is prominently involved in leukemia development (Yu et al., 2020; Pikman and Stieglitz, 2021). Our study findings were consistent with these characteristics. In both case #5 and case #17, we detected variants in genes (*MLH1* and *MSH6*) that play a role in DNA repair mechanisms. However, the *TNFRSF9* variant, an NFKB pathway member, was found in case #13, who belonged to family with Hodgkin’s lymphoma.

Some of the affected family members did not provide samples for segregation analysis. This major limitation emphasizes the critical need for biobanking of samples from affected individuals. However, after the variant was detected, some families declined to provide additional samples due to anxiety. Beyond the general concerns about knowing one’s cancer susceptibility, families also worry about the cancer risk in unaffected but variant-positive siblings (Bon et al., 2022). While the patients in this study were enrolled from the main hematology/oncology centers, the demographic distribution was diverse. However, patients living in rural areas were often unable to travel, and the clinical features could not be assessed, limiting detailed phenotyping. Furthermore, the underrepresentation of Turkish genomes in public databases complicates variant analysis and interpretation, posing significant challenges for precision medicine and genetic diagnostics in Türkiye. Thus, the genetic background of approximately 35% of the cohort remained unclear.

In conclusion, we identified known and novel variants that may contribute to genetic predisposition to childhood leukemia/lymphoma. Our findings can guide physicians in developing treatment strategies, surveillance plans, and genetic counseling approaches for the family members at risk. Our study also raised awareness of childhood cancer predisposition among the participating hemato-oncology clinics across multiple hospitals in Türkiye. This study represents the largest cohort in Türkiye for investigating genetic predisposition to childhood leukemia/lymphoma.

## Data Availability

The raw data supporting the findings of this article will be made available by the authors upon reasonable request.
