# Supplementary material for: Genetic heterogeneity in childhood leukemia/lymphoma: a Turkish cohort with strong predisposition
Source: Front Genet. 2025 Sep 9;16:1624306. doi: 10.3389/fgene.2025.1624306 (PMC12454056; doi:10.3389/fgene.2025.1624306)
Supplement: Supplementary file 1 [file DataSheet2.pdf]

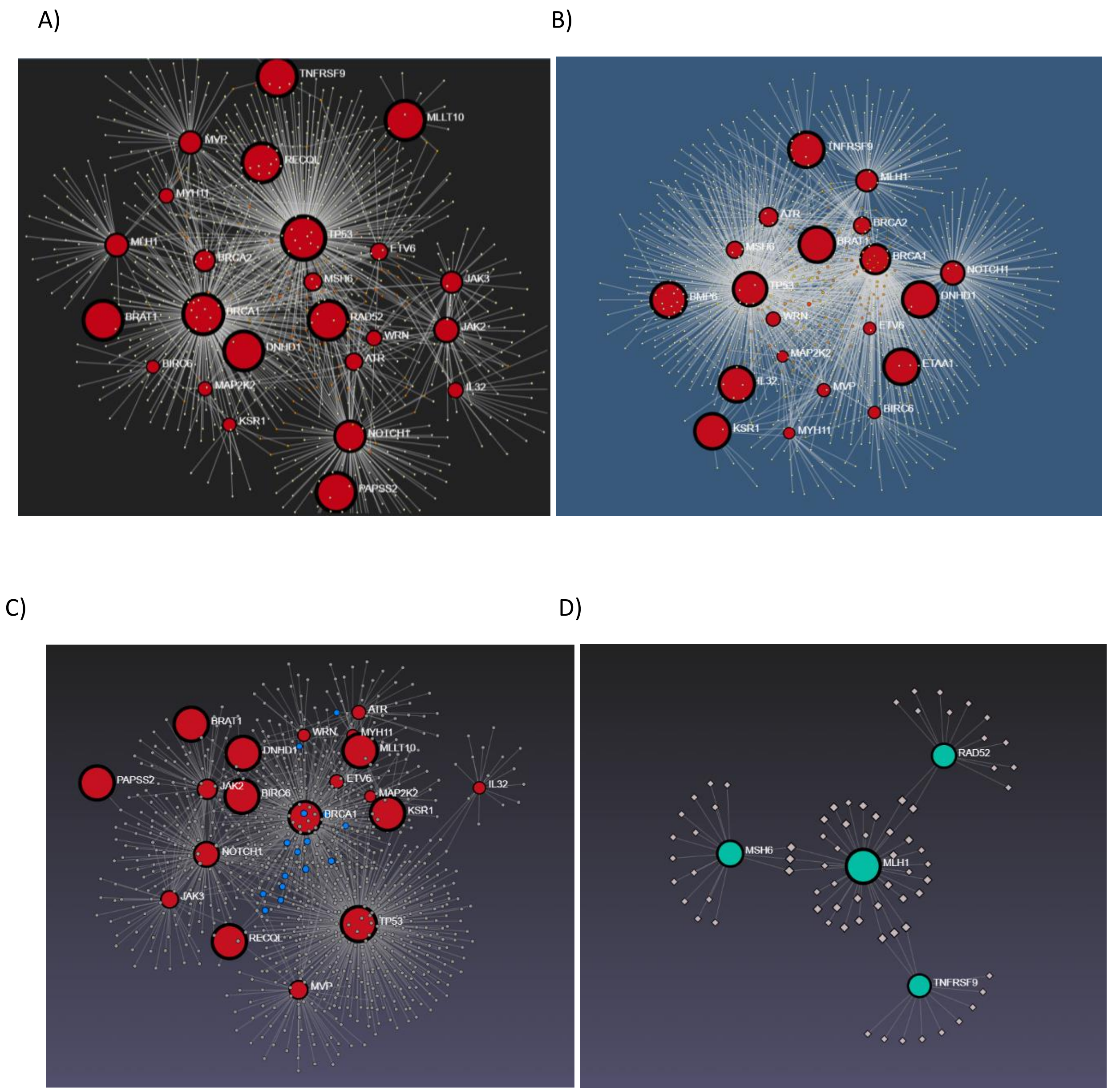


**Supplemental Figure 3**: (A) NetworkAnalyst results for all candidate genes. (B) NetworkAnalyst results for candidate causative genes. (C) NetworkAnalyst results for genes observed only in leukemia cases. (D) NetworkAnalyst results for genes observed only in lymphoma cases.
