# Supplementary material for: Genetic heterogeneity in childhood leukemia/lymphoma: a Turkish cohort with strong predisposition
Source: Front Genet. 2025 Sep 9;16:1624306. doi: 10.3389/fgene.2025.1624306 (PMC12454056; doi:10.3389/fgene.2025.1624306)
Supplement: Supplementary file 2 [file DataSheet4.pdf]

TP53: p.Trp146X

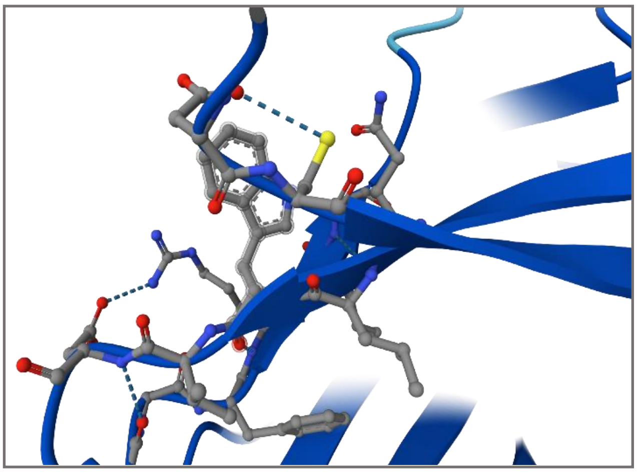

Field	Value
AF Model	AF-P04637-F1
Model	1
Instance	1_555
Chain and Residue	A
UniProt ID and Residue	UNP P04637 146 W
pLDDT Score (1 Residue)	97.91 (Very High)

ETV6:c.464-2A>G splicing

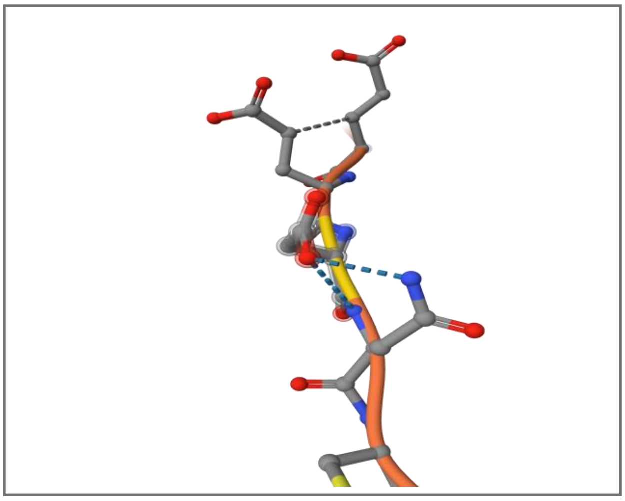

Field	Value
AF Model	AF-P41212-F1
Model	1
Instance	1_555
Chain and Residue	A
UniProt ID and Residue	UNP P41212 155 D
pLDDT Score (1 Residue)	50.60 (Low)

SpliceAI: acceptor loss:0.98/acceptor gain:0.59

BMP6: p.Gly336Glu

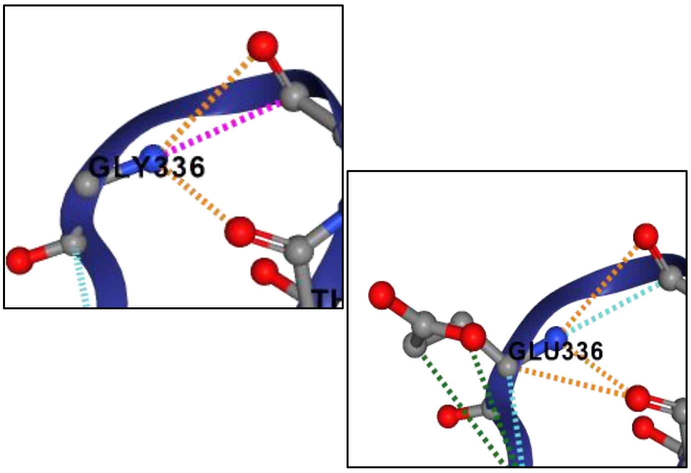

Field	Value
AF Model	AF-P22004-F1
Model	Model 1
Instance	Instance 1_555
Chain and Residue	A
UniProt ID and Residue	UNP P22004 336 G
pLDDT Score (1 Residue)	91.90 (Very High)

Predicted Stability Change: -0.31kcal/mol Destabilizing

JAK2: p.Arg683Gly

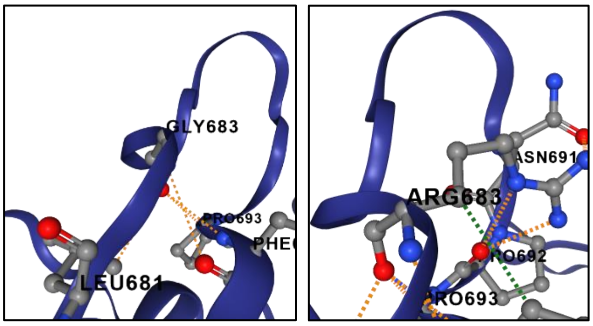

Field	Value
AF Model	AF-O60674-F1
Model	Model 1
Instance	Instance 1_555
Chain and Residue	A
UniProt ID and Residue	UNP O60674 683 R
pLDDT Score (1 Residue)	93.87 (Very High)

Predicted Stability Change: -1.44 kcal/mol Destabilizing

JAK3: p.Gln1083X

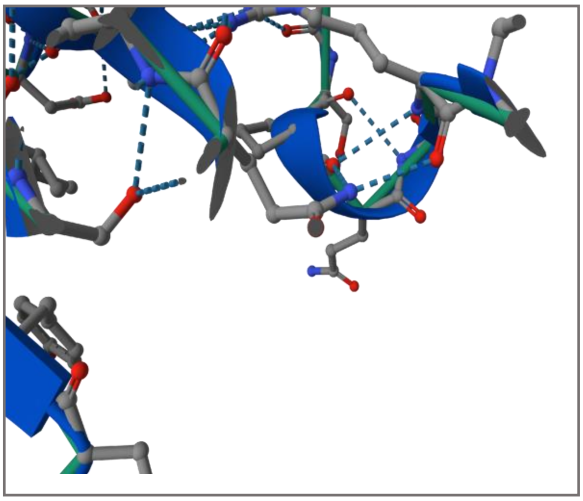

Field	Value
AF Model	AF-P52333-F1
Model	Model 1
Instance	Instance 1_555
Chain and Residue	A
UniProt ID and Residue	UNP P52333 1083 Q
pLDDT Score (1 Residue)	94.78 (Very High)

MSH6: p.Ile1313SerfsTer7

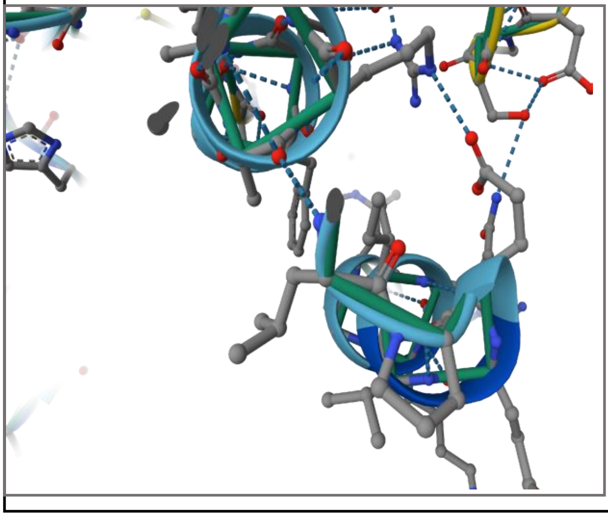

Field	Value
AF Model	AF-P52701-F1
Model	Model 1
Instance	Instance 1_555
Chain and Residue	A
UniProt ID and Residue	UNP P52701 1313 I
pLDDT Score (1 Residue)	87.84 (Confident)

MYH11: p.Arg431Cys

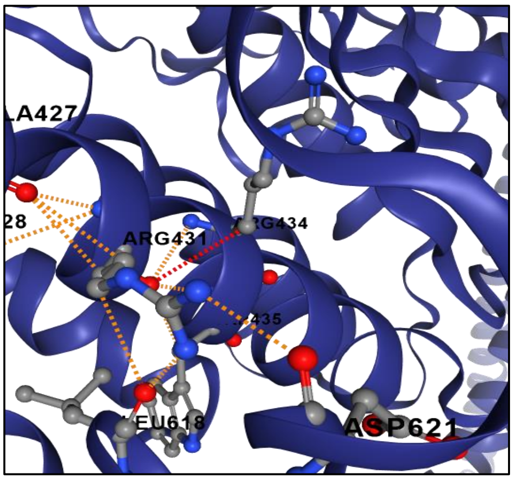

Field	Value
AF Model	AF-P35749-F1
Model	Model 1
Instance	Instance 1_555
Chain and Residue	A
UniProt ID and Residue	UNP P35749 431 R
pLDDT Score (1 Residue)	91.35 (Very High)

Predicted Stability Change: -0.69 kcal/mol Destabilizing

ATR: p.Gly1758Ala

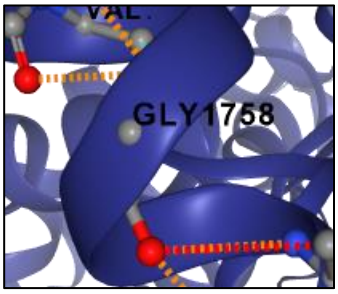

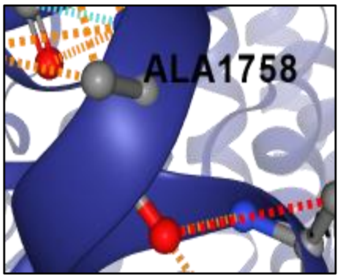

**Field**	**Value**
AF Model	AF-Q13535-F1
Model	Model 1
Instance	Instance 1_555
Chain and Residue	A
UniProt ID and Residue	UNP Q13535 1758 G
pLDDT Score (1 Residue)	84.05 (Confident)

Predicted Stability Change: -0.19 kcal/mol Destabilizing

NOTCH1: Gly977Arg

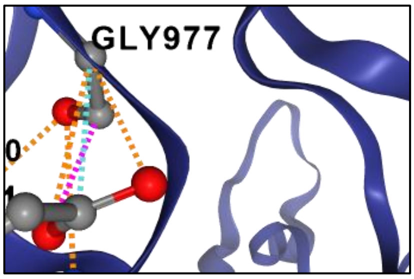

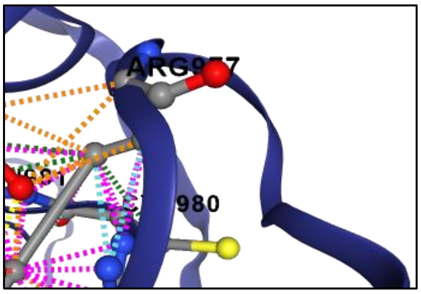

**Field**	**Value**
AF Model	AF-P46531-F1
Model	Model 1
Instance	Instance 1_555
Chain and Residue	A
UniProt ID and Residue	UNP P46531 977 G
pLDDT Score (1 Residue)	57.38 (Low)

Predicted Stability Change: -0.25 kcal/mol Destabilizing

MVP: p.Arg766X

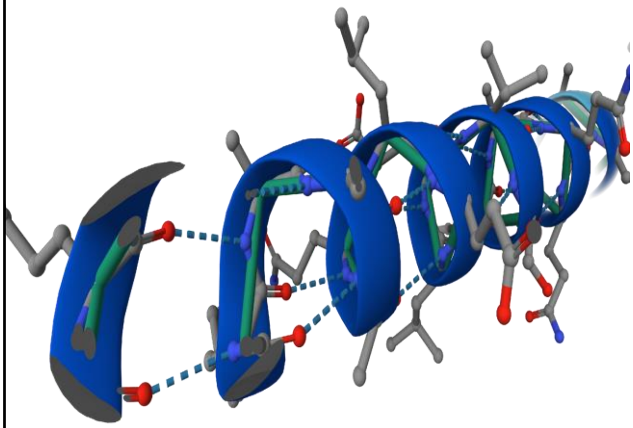

Field	Value
AF Model	AF-Q14764-F1
Model	Model 1
Instance	Instance 1_555
Chain and Residue	A
UniProt ID and Residue	UNP Q14764 766 R
pLDDT Score (1 Residue)	91.61 (Very High)

IL32: p.Asp172Glufs*

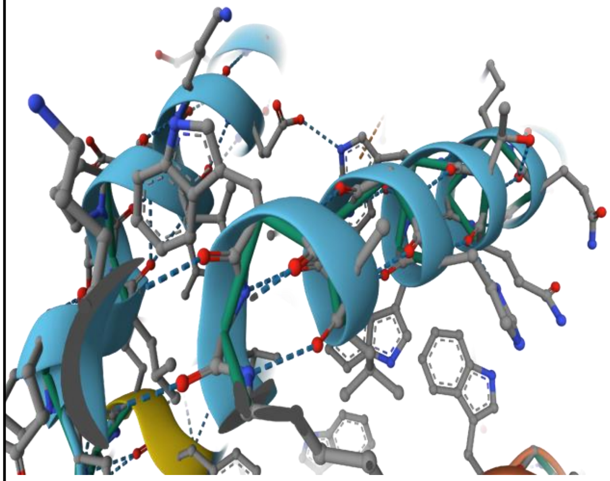

Field	Value
AF Model	AF-P24001-F1
Model	Model 1
Instance	Instance 1_555
Chain and Residue	A
UniProt ID and Residue	UNP P24001 172 V
pLDDT Score (1 Residue)	80.59 (Confident)

RAD52: p.Glu130Lys

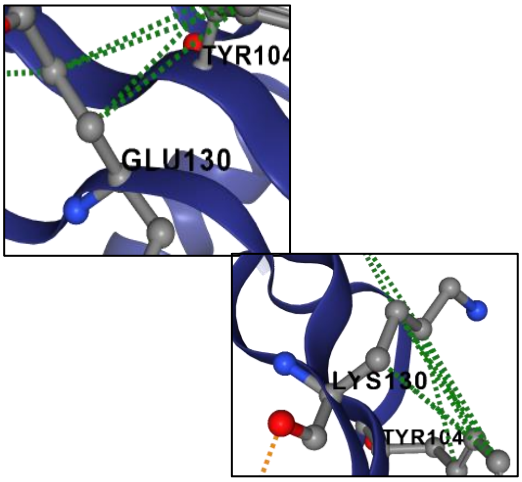

Field	Value
AF Model	AF-P43351-F1
Model	Model 1
Instance	Instance 1_555
Chain and Residue	A
UniProt ID and Residue	UNP P43351 130 E
pLDDT Score (1 Residue)	96.49 (Very High)

Predicted Stability Change: -0.12 kcal/mol Destabilizing

WRN: p.Gly574Val

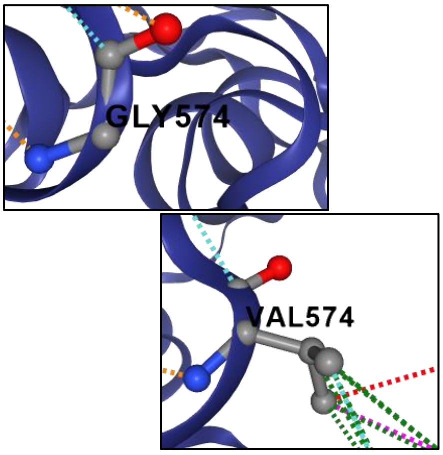

Field	Value
AF Model	AF-P43351-F1
Model	Model 1
Instance	Instance 1_555
Chain and Residue	A
UniProt ID and Residue	UNP P43351 130 E
pLDDT Score (1 Residue)	96.49 (Very High)

Predicted Stability Change: -1.35 kcal/mol Destabilizing

MUTYH: p.Pro267Leu

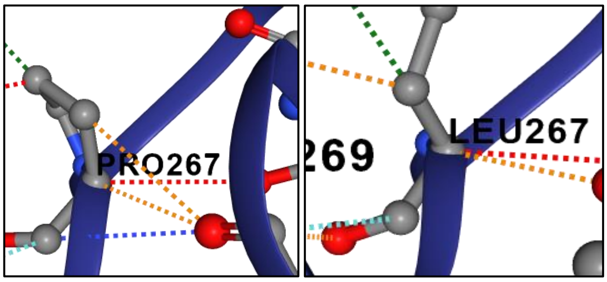

Field	Value
AF Model	AF-A0A5F9ZI14-F1
Model	Model 1
Instance	Instance 1_555
Chain and Residue	A
UniProt ID and Residue	UNP A0A5F9ZI14 267 P
pLDDT Score (1 Residue)	97.25 (Very high)

Predicted Stability Change: -0.52 kcal/mol Destabilizing

TNFRSF9:c.413+6T>C splicing

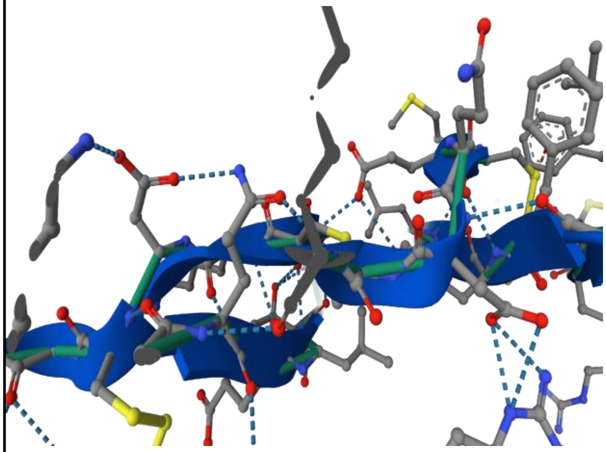

Field	Value
AF Model	AF-Q07011-F1
Model	Model 1
Instance	Instance 1_555
Chain and Residue	A
UniProt ID and Residue	UNP Q07011 117 C
pLDDT Score (1 Residue)	96.23 (Very High)
pLDDT Score (255 Residues avg.)	81.93 (Confident)

SpliceAI: acceptor loss:0.54/donor loss:0.65

BCNP1: c.1554+1G>T

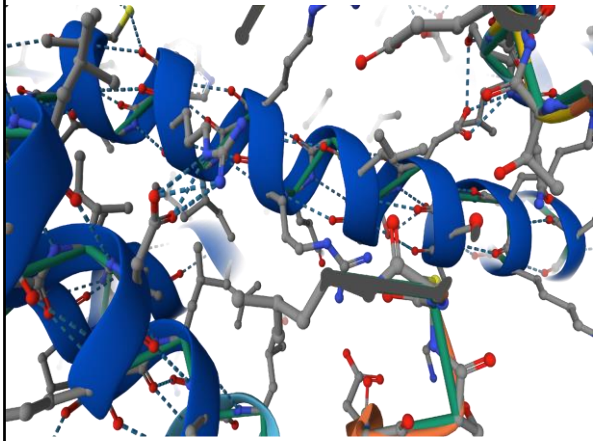

Field	Value
Protein	Protein Niban 3
AF Model	AF-Q86XR2-F1
Model	Model 1
Instance	Instance 1_555
Chain and Residue	A
UniProt ID and Residue	UNP Q86XR2 524 R
pLDDT Score (1 Residue)	93.69 (Very High)
pLDDT (697 residues avg.)	77.37 (Confident)

SpliceAI: acceptor loss:0.72/donor loss:0.81

RECQL: p.Met201Ile

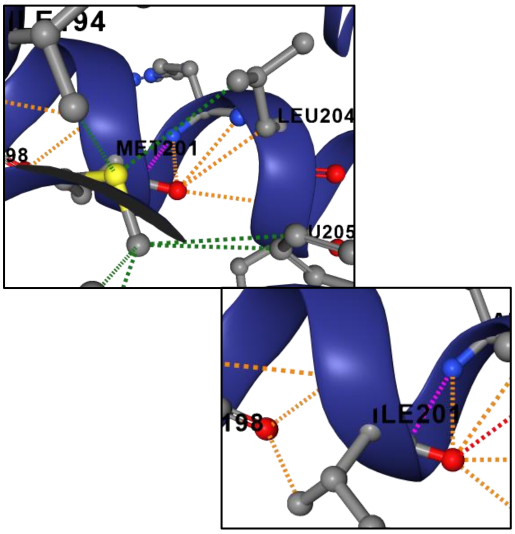

Field	Value
Protein	ATP-Dependent DNA Helicase Q1
AF Model	AF-P46063-F1
Model	Model 1
Instance	Instance 1_555
Chain and Residue	A
UniProt ID and Residue	UNP P46063 201 M
pLDDT Score (1 Residue)	97.33 (Very High)

Predicted Stability Change: -0.71 kcal/mol Destabilizing

TGFBRS1: p.Thr346Pro

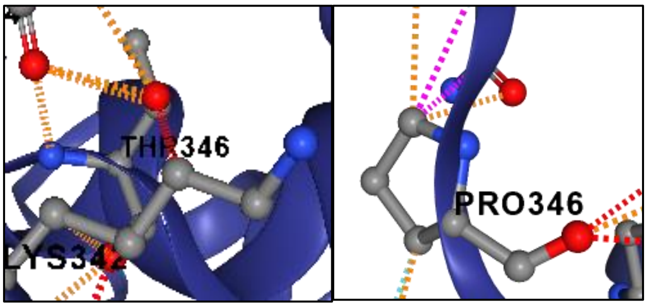

Field	Value
**AF Model**	AF-P36897-F1
**Model**	Model 1
**Instance**	Instance 1_555
**Chain and Residue**	A
**UniProt ID and Residue**	UNP P36897 346 T
**pLDDT Score (1 Residue)**	98.40 (Very high)

Predicted Stability Change: 0.35 kcal/mol Stabilizing

PAPPSS2: p.Arg585Ter

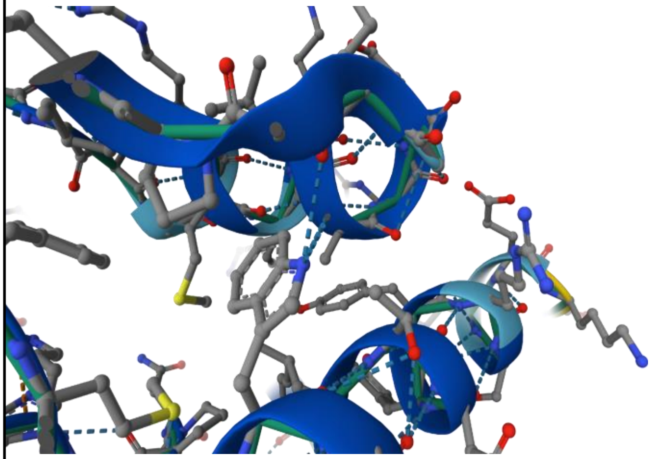

**Field**	**Value**
AF Model	AF-O95340-F1
Model	Model 1
Instance	Instance 1_555
Chain and Residue	A
UniProt ID and Residue	UNP O95340 585 A
pLDDT Score (1 Residue)	92.10 (Very High)

BRCA1: p.Gln1756ProfsTer74

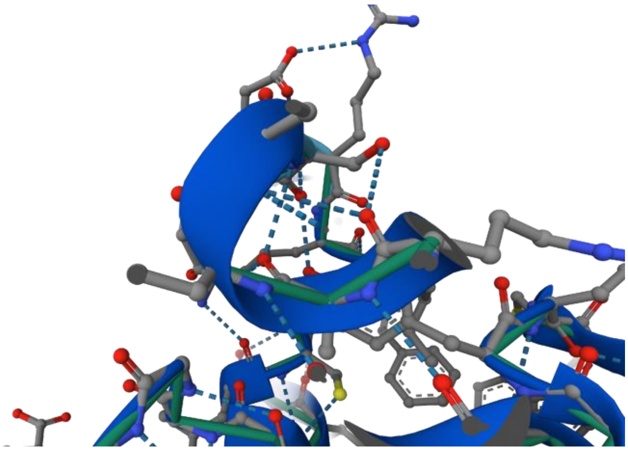

**Field**	**Value**
AF Model	AF-P38398-F1
Model	Model 1
Instance	Instance 1_555
Chain and Residue	A
UniProt ID and Residue	UNP P38398 1756 Q
pLDDT Score (1 Residue)	80.59 (Confident)

BRCA2: p.Ser1123Gly

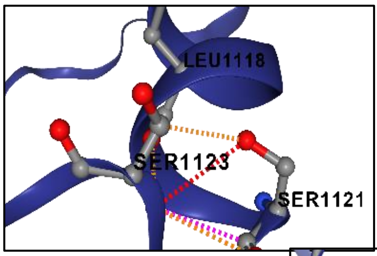

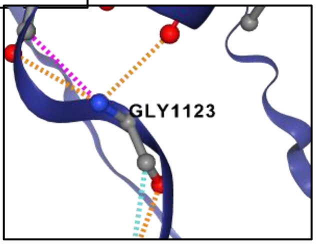

Field	Value
AF Model	AF-A0A386IPX8-F1
Model	Model 1
Instance	Instance 1_555
Chain and Residue	A
UniProt ID and Residue	UNP A0A386IPX8 1123 S
pLDDT Score (1 Residue)	48.03 (Very Low)

Predicted Stability Change: -0.11 kcal/mol destabilizing

ABCC6: p.Arg1357Gln

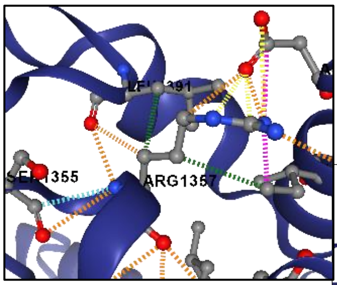

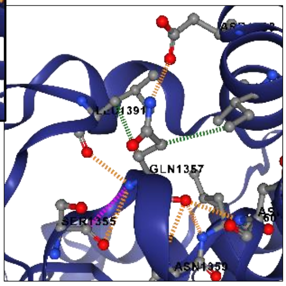

Field	Value
AF Model	AF-O95255-F1
Model	Model 1
Instance	Instance 1_555
Chain and Residue	A
UniProt ID and Residue	UNP O95255 1357 R
pLDDT Score (1 Residue)	92.24 (Very High)

Predicted Stability Change: -0.11 kcal/mol destabilizing
