# Supplementary material for: Genetic heterogeneity in childhood leukemia/lymphoma: a Turkish cohort with strong predisposition
Source: Front Genet. 2025 Sep 9;16:1624306. doi: 10.3389/fgene.2025.1624306 (PMC12454056; doi:10.3389/fgene.2025.1624306)
Supplement: Supplementary file 3 [file DataSheet6.docx]

**Supplemental File 2: Case Vignettes**

Here, we provide the details on the clinics, enrollment conditions, and the genomic findings of the index cases. Additionally, a detailed pedigree and family history are added. The Jongman Criteria are summarized in the Table below.

| **Jongman Criteria (Jongman et al., 2016)** | **Criteria No** |
| --- | --- |
| Family history  >3-generation family pedigree | 1 |
| Detection of one of the different cancers (e.g. early-onset, ALL (lhypodiploid), ALL (ring chromosome 21), ALL (Robertsonian translocation 15;21), ALL relapse (*TP53* mutated), AML (Monosomy 7), Basal cell carcinoma, Botryoid Rhabdomyosarcoma) | 2 |
| The presence of two or more tumors in the patient (e.g. secondary, bilateral, multifocal) | 3 |
| A case with congenital anomaly/  specific different symptoms | 4 |
| A child with severe treatment-related toxicity | 5 |

**Case #01 (WES)**

The index case was admitted to the Pediatric Hematology and Oncology Department of Medical Park Samsun Hospital, in 2017 at the age of 15 and diagnosed with B-ALL. Despite treatment toxicity, she was in remission after the treatment. In 2019 at the age of 17, she underwent surgical excision of the left breast due to a benign fibroadenoma. In addition, the patient and her mother were beta-thalassemia carriers. The patient did not present facial dysmorphism. The mother declared no known consanguinity but was from the same village as her spouse. The father of the index case died due to stomach cancer at the age of 45, and her paternal grandfather died due to an unknown type of leukemia at the age of 55. Her paternal uncle was diagnosed with an unknown type of leukemia and is still under surveillance. Her paternal aunt was diagnosed with ovarian cancer at the age of 30. We enrolled this patient with the Jongman criteria of 1,2 and 5 and performed WES analysis from the remission peripheral blood sample. Gradual filtering detected a known heterozygous pathogenic ***TP53* c.437G>A:p.Trp146Ter stopgain**  **variant (ClinVar ID:** 634785). During the study at the age of 18, the patient presented a primary AML (Jongman criteria 2), and her bone marrow sample with a blast load of 34% revealed the same *TP53* variant. The segregation analysis from the blood samples of her mother and siblings showed that they were WT for the same variant. Since the father of the patient was deceased, the other affected family members refused to provide any samples and genetic counseling. Currently, her treatment continues as AML BFM protocol.


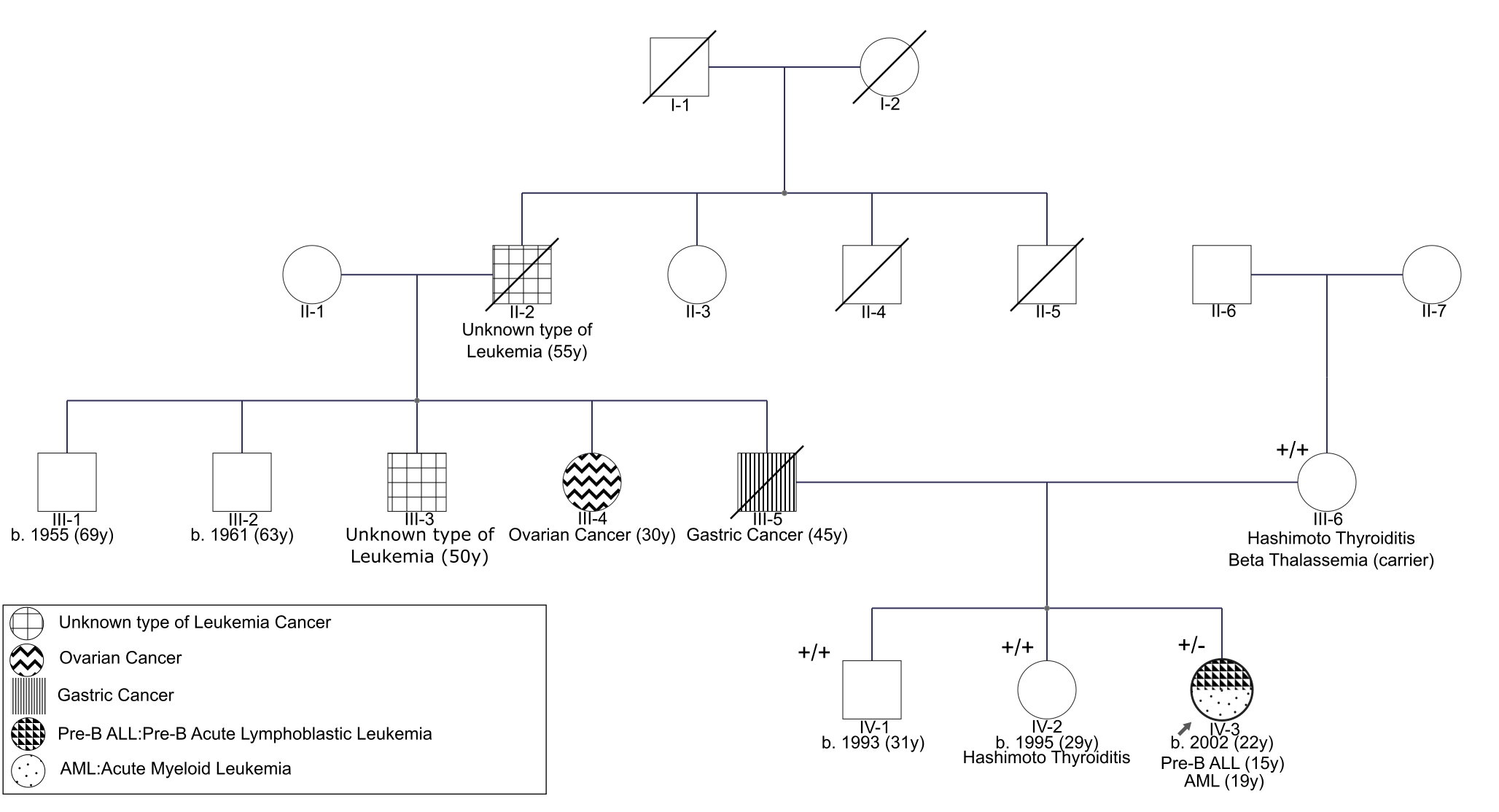


**Supp File 2. 1:** Case#1 pedigree (+/+ Wild Type (WT), +/- Heterozygous)

**Case Vignette #02 (WES)**

Case #02 was hospitalized at the Pediatric Hematology-Oncology Department of Ankara Şehir Hospital with a B-ALL diagnosis at nine. She presented anguli oris hypoplasia, vascular lesions and thrombocytopenia. She presented low ear and dental problems. We enrolled this patient with the Jongman criteria of 1,2 and 4. There is no known consanguinity in her parents. The patient's brother was also diagnosed with T-ALL when he was 4 years old, and he was diagnosed with AML when he was 16 years old. There was also a family history of miscarriage of unknown cause. We ran the bone marrow sample of the index case and the peripheral blood sample of her affected brother through WES and performed joint analysis. WES revealed a pathogenic heterozygous splicing variant of the ***ETV6*** **gene** **c.464-2A>G in both cases**. The variant has not been previously reported in the ClinVar database. During the study, the index's affected brother died. The segregation analysis showed that the patient's father was WT. However, the heterozygous variant was detected in the mother and unaffected sibling. The transplantation was planned for the index case, and the donor was decided to be non-related due to the carrier risk. Genetic counseling suggested long-term follow-up for the heterozygous sibling.


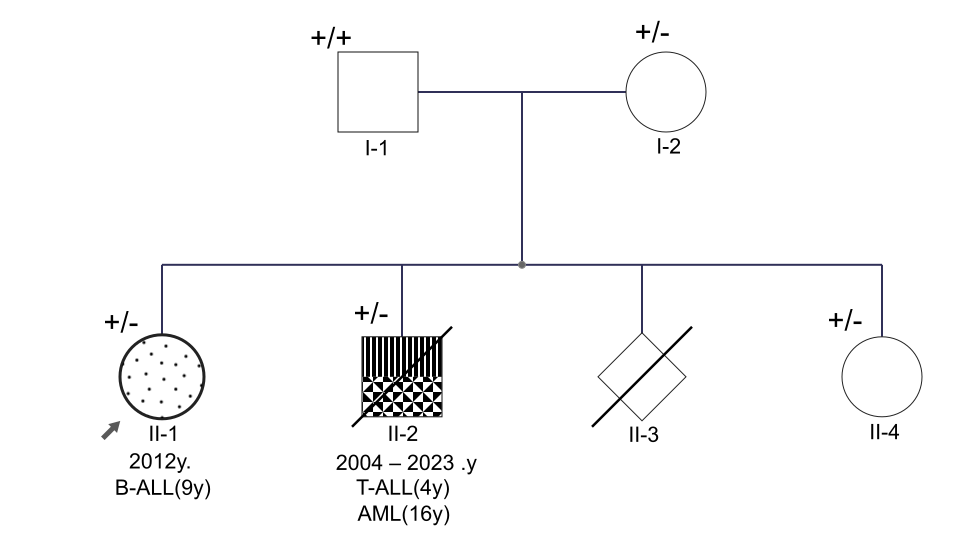


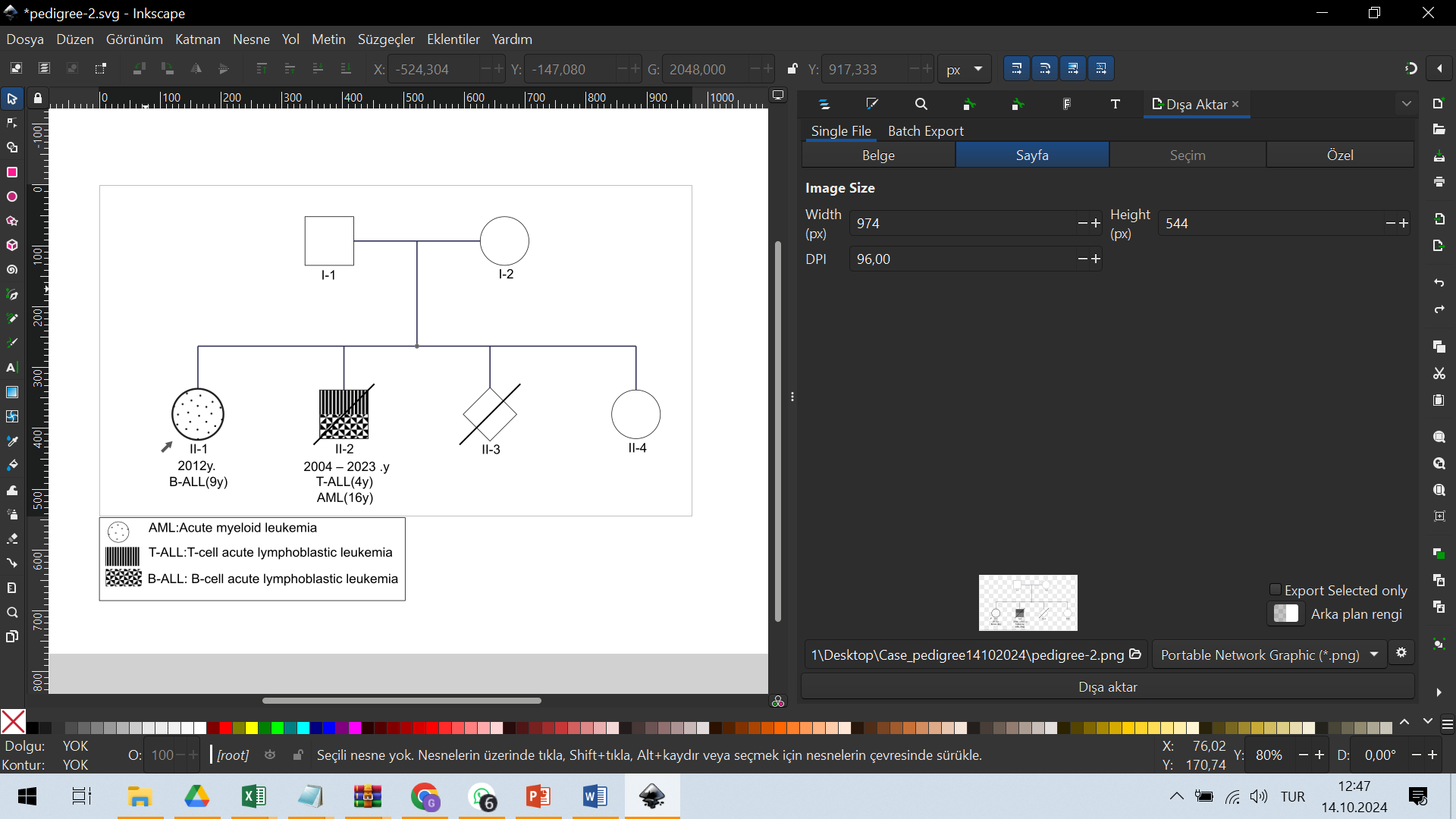


**Supp File 2. 2:** Case#2 pedigree (+/+ WT, +/- Heterozygous)

**
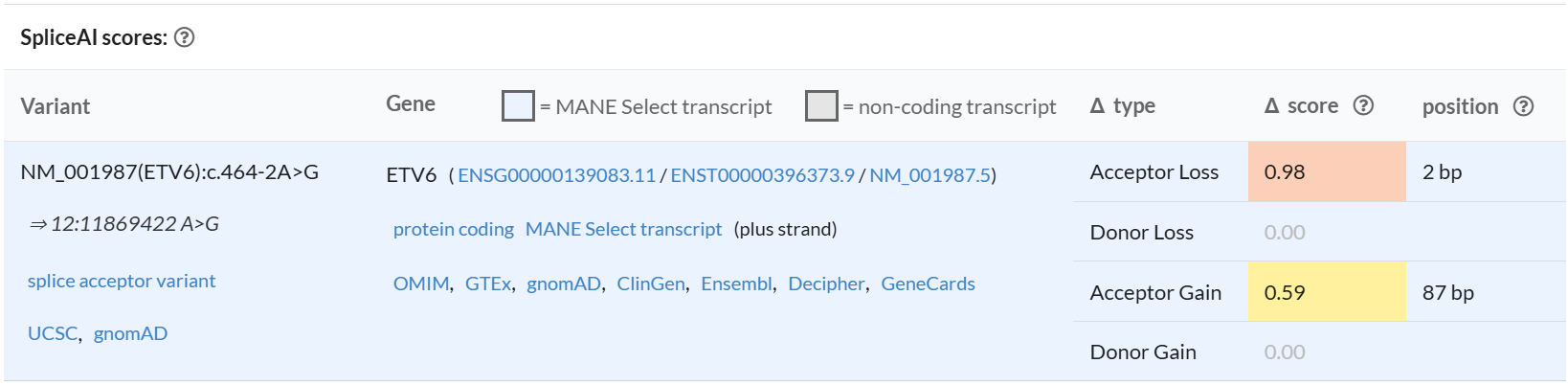
**

**Supp File 2. 3:** SpliceAI score of *ETV6* c.464-2A>G gene variant

**Case Vignette #03 (WES)**

Case #03 was a 14-year-old patient born into a consanguinous marriage and was diagnosed with B-ALL at the age of 9 at the Pediatric Hematology-Oncology Department of Ankara Şehir Hospital. The patient presented mild facial dysmorphisms: a prominent forehead, thick eyebrows, large eyes, large ears, flat philtrum hepatosplenomegaly, and hepatosteatosis. The affected brother of the patient was diagnosed with Hodgkin’s Lymphoma when he was 8 years old, and there were no dysmorphic features. Samples of these two siblings were run through whole exome sequencing and analyzed together. We have determined two candidate variants. One was a homozygous known (ClinVar ID: 1334259) variant of uncertain significance (VUS) splicing variant of the ***MAP2K2* gene c.907C>T p.R303C.** Together with the PP3, PM2, and PP1 evidence, we classified the variant as ‘likely pathogenic’. Sanger sequencing confirmed the candidate variant; the patient's parents were heterozygous, and unaffected siblings were observed as WT.

The other candidate variant was a heterozygous likely pathogenic ***BMP6 c.1007G>A p.Gly336Glu***. The variant was confirmed by Sanger sequencing as heterozygous in affected siblings. The patient's mother, father and unaffected brother were also heterozygous, and his unaffected sister was WT.

**a)**


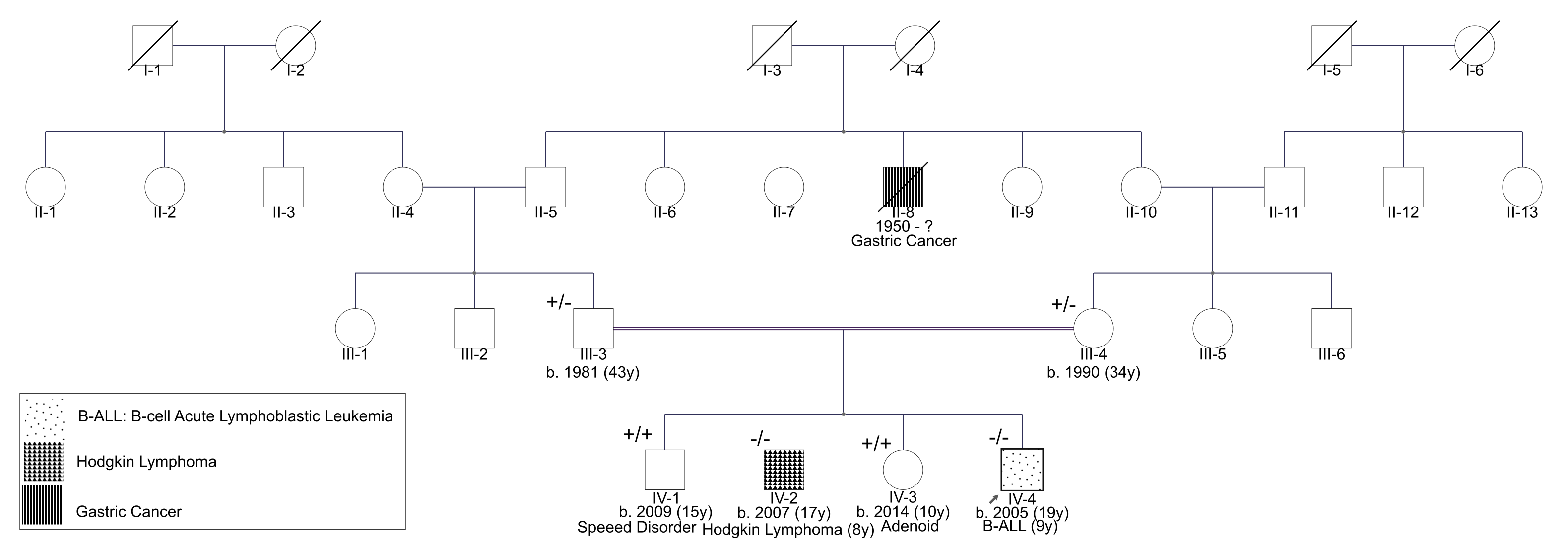


**b) *MAP2K2* gene c.907C>T*
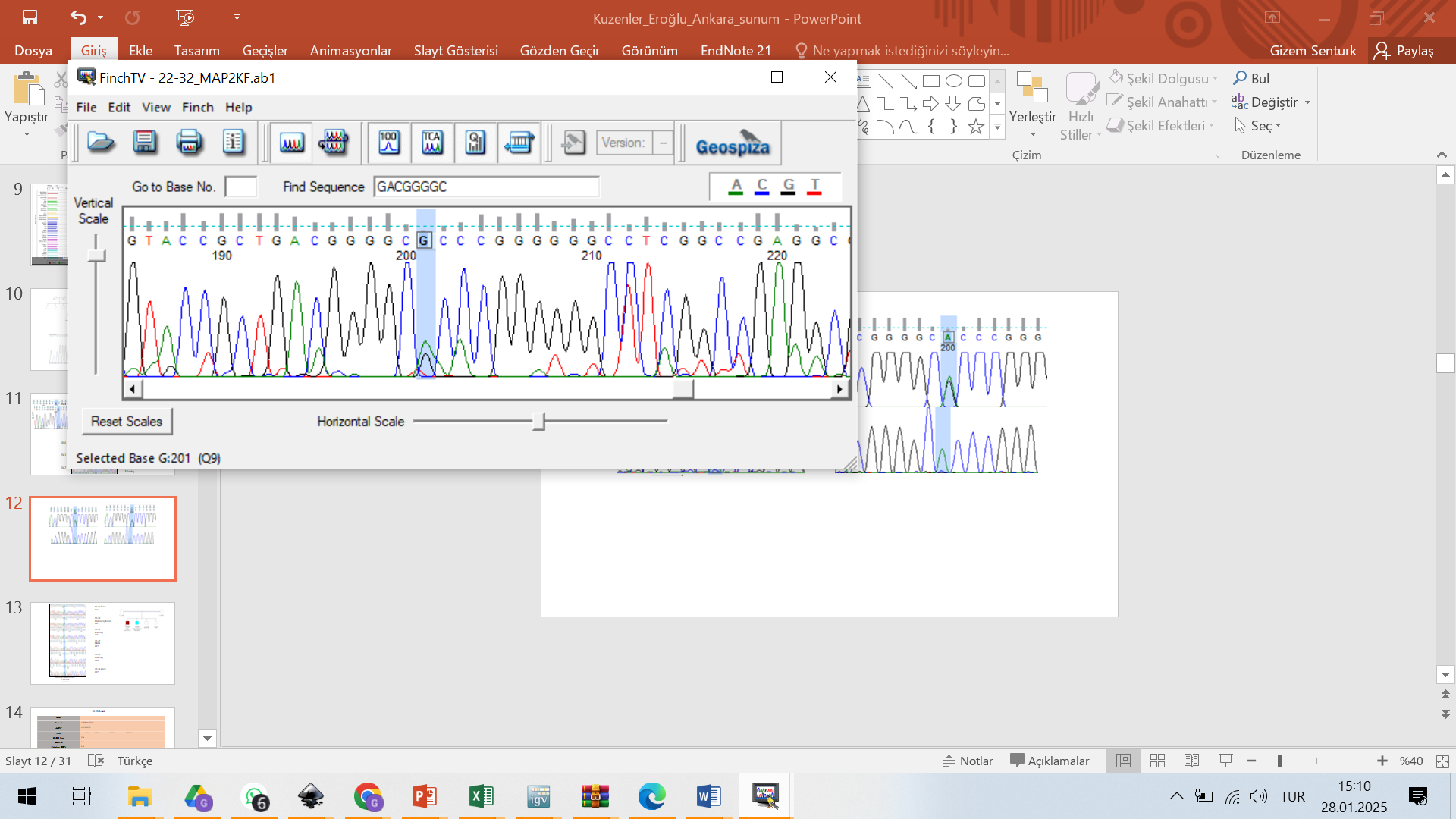
***
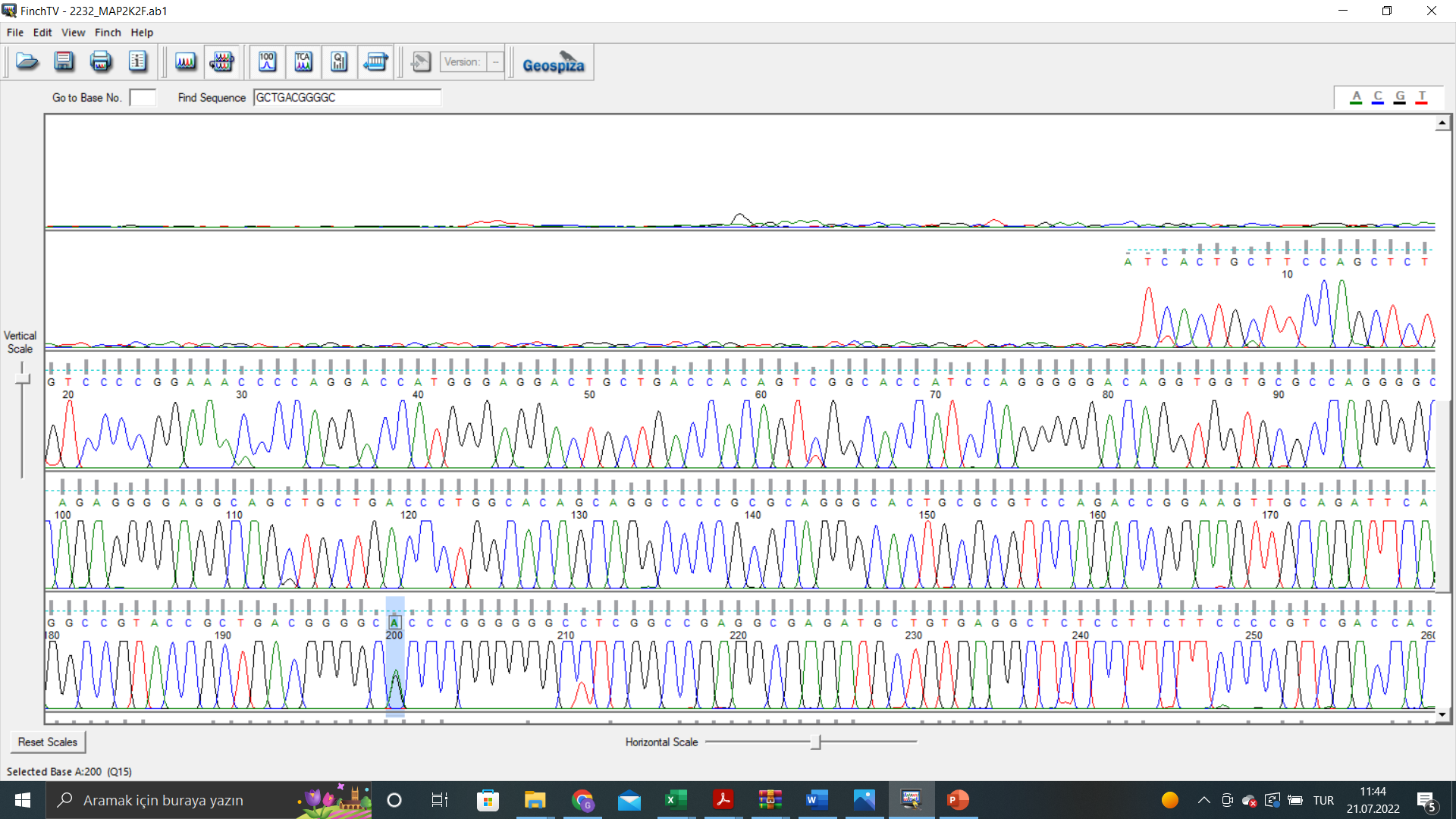

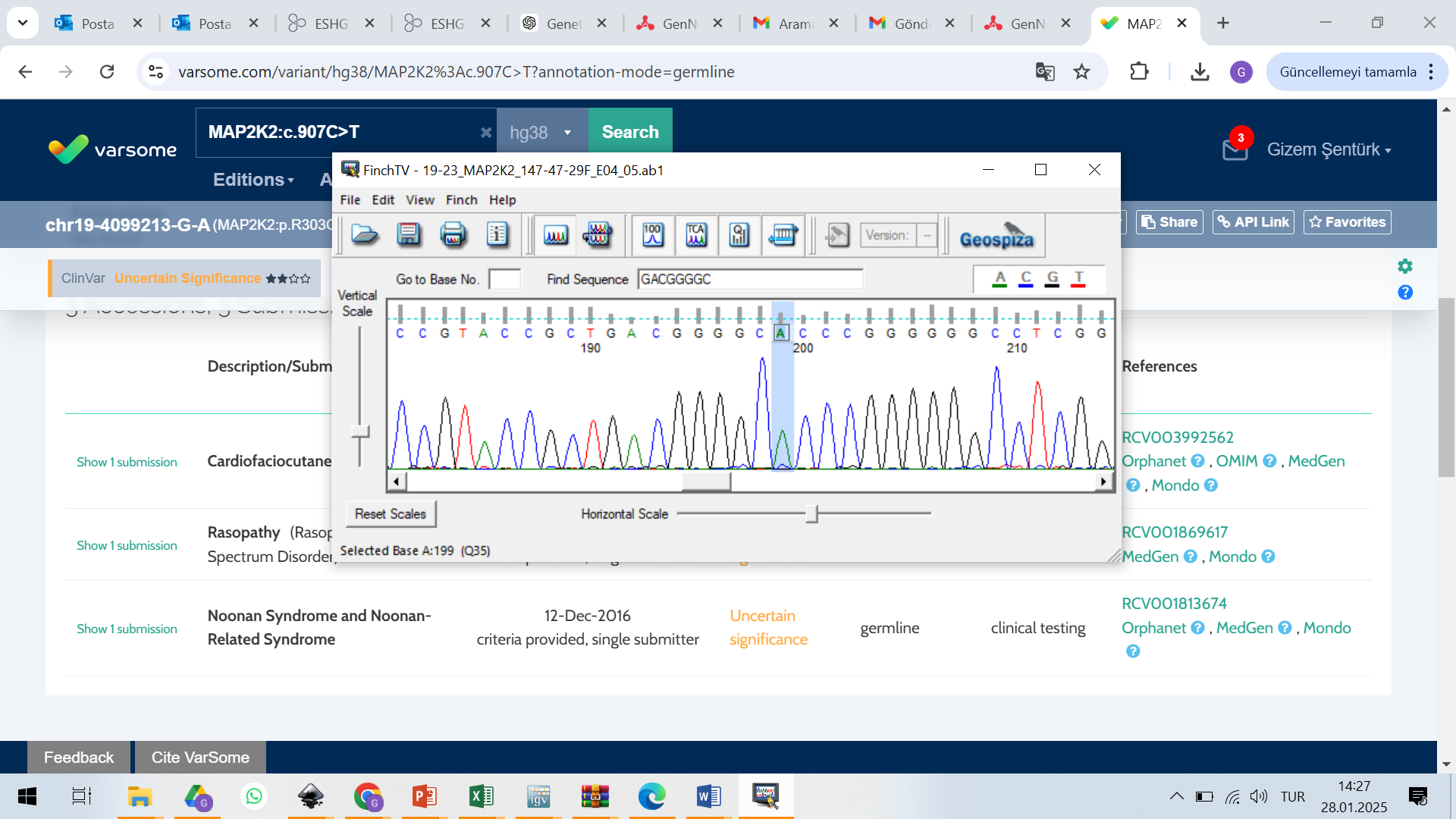


IV-2 III-3 III-4

**Supp File 2. 4:** a) Case#3 pedigree (+/+ Wild type, +/- Heterozygous, -/- Homozygous for *MAP2K2* candidate gene variant). b) Result of sanger sequencing in the index case, mother and father

**Case Vignette #04 (WES)**

Case **#**4 was diagnosed with pre-B-ALL at the age of three and was enrolled due to a positive family history. The index case was born into a consanguineous marriage. The father and the paternal uncle were diagnosed with Hodgkin Lymphoma at the ages of 20 and 25, respectively. Father's cousin was also diagnosed with acute lymphoblastic leukemia at the age of 25. The parallel WES analysis of the index case and the father revealed two likely pathogenic variants. One was a heterozygous frameshift deletion at the exon 5 of the ***ETAA1* gene (c.2565_2568del:p.K855fs)**. Sanger sequencing confirmed the variant in the index case; the father, paternal uncle, and unaffected younger brother were positive for the variation. The mother and the other unaffected brother were WT. The variant has not been previously reported in the ClinVar database. The other candidate variant was ***DNHD1* c.7888C>T:p.R2630X** stop gain alteration. The Sanger analysis confirmed the variant in the index case affected the father and affected uncle, while it was observed as WT in the mother and unaffected siblings. After detecting the candidate variant, both the affected father and uncle developed Hodgkin Lymphoma recurrence in 2023. Also, his father developed soft tissue sarcoma in the same year.

a)


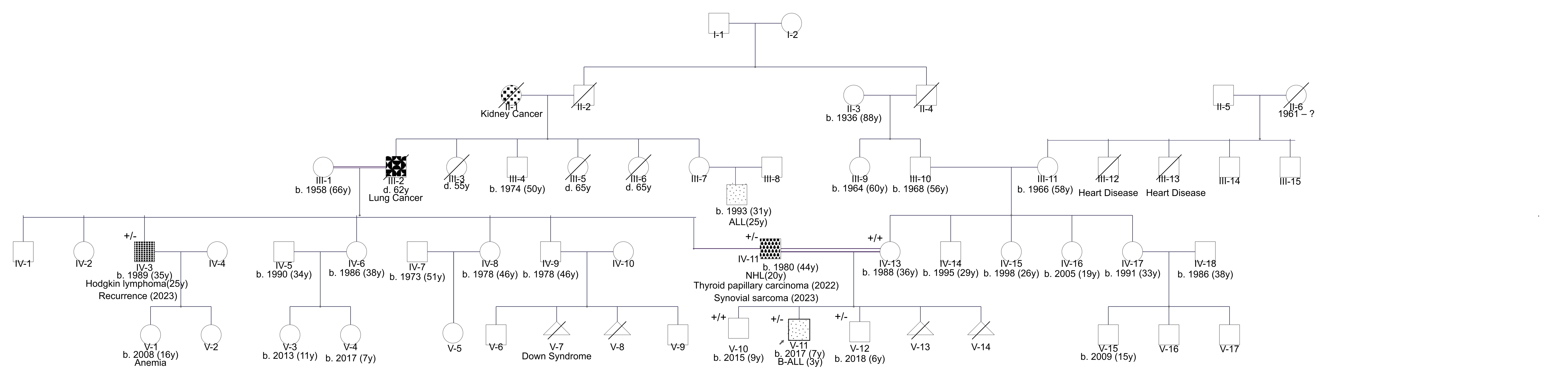


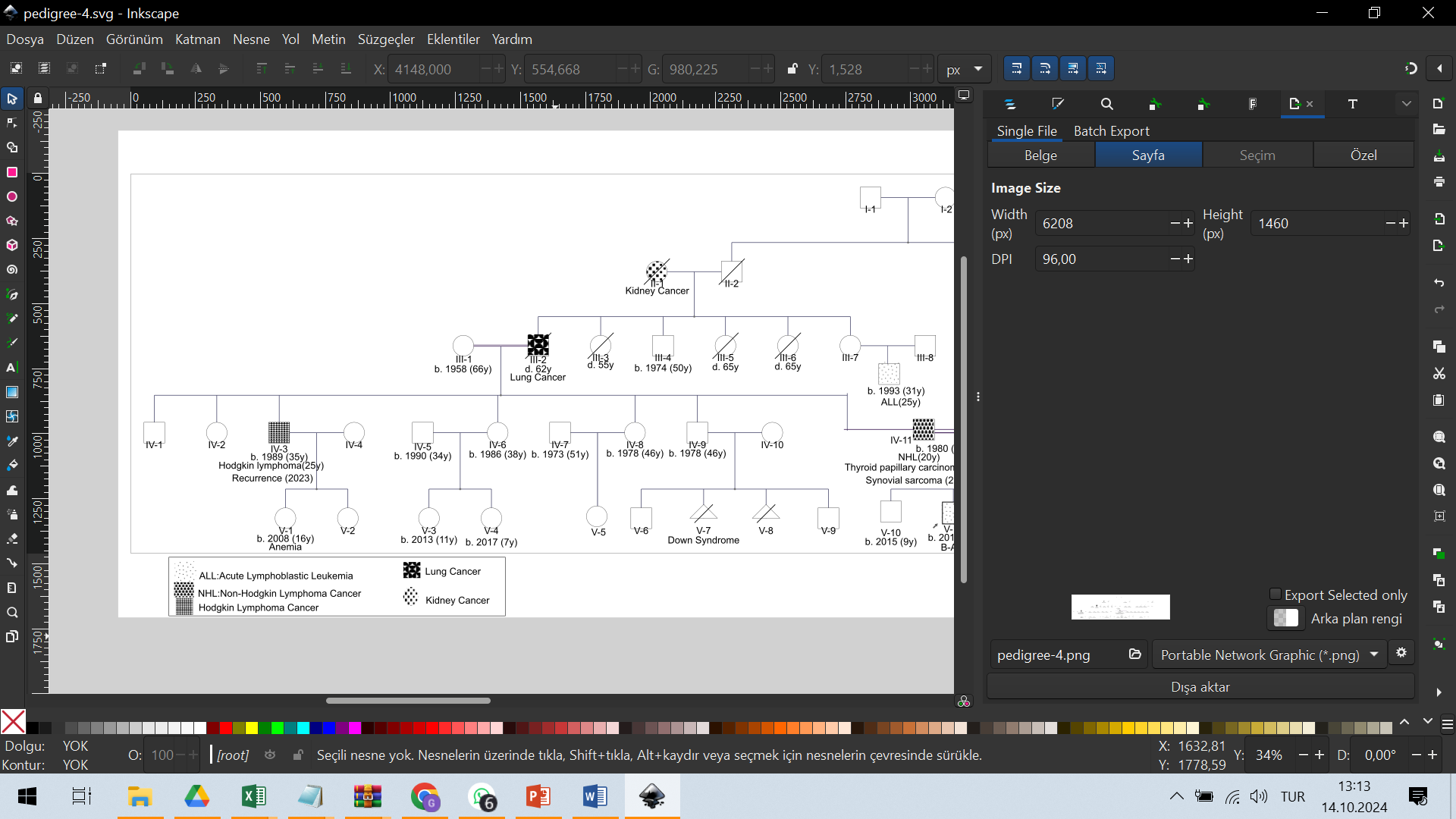


b)

**
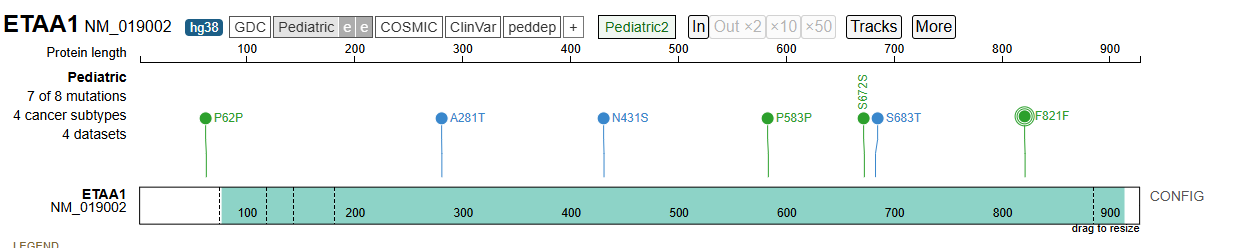
**

**
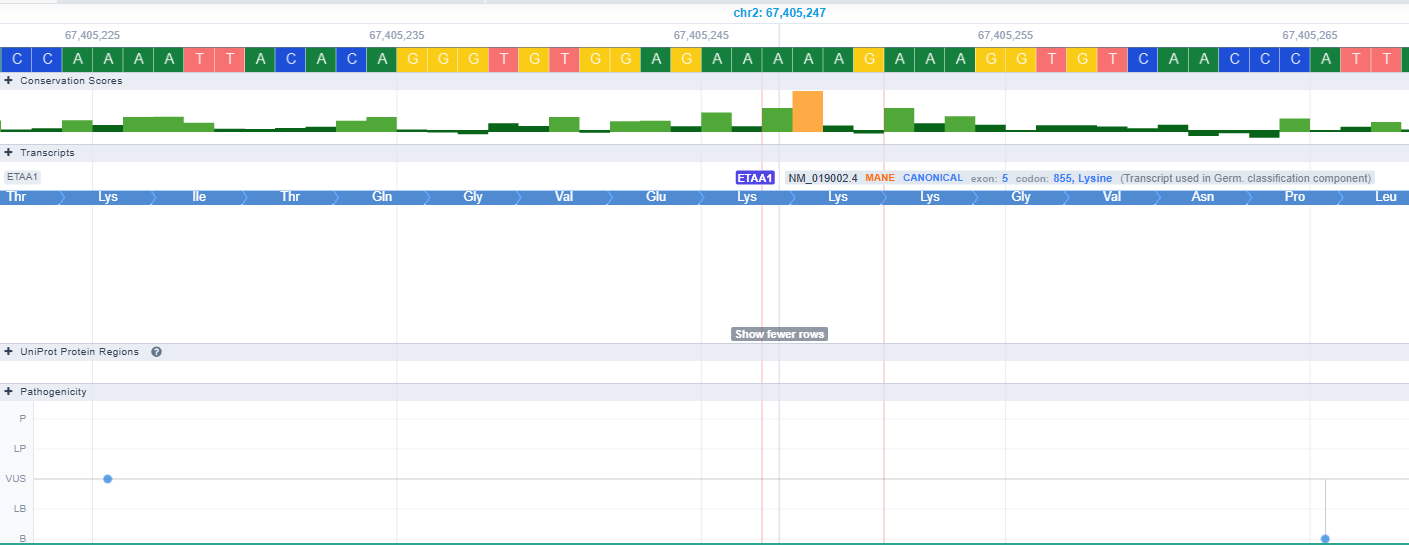
**

c)

**
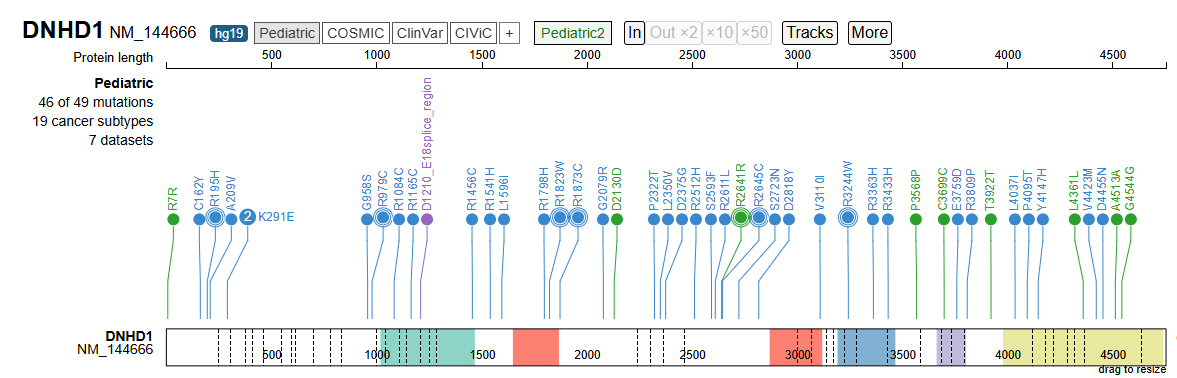
**

**
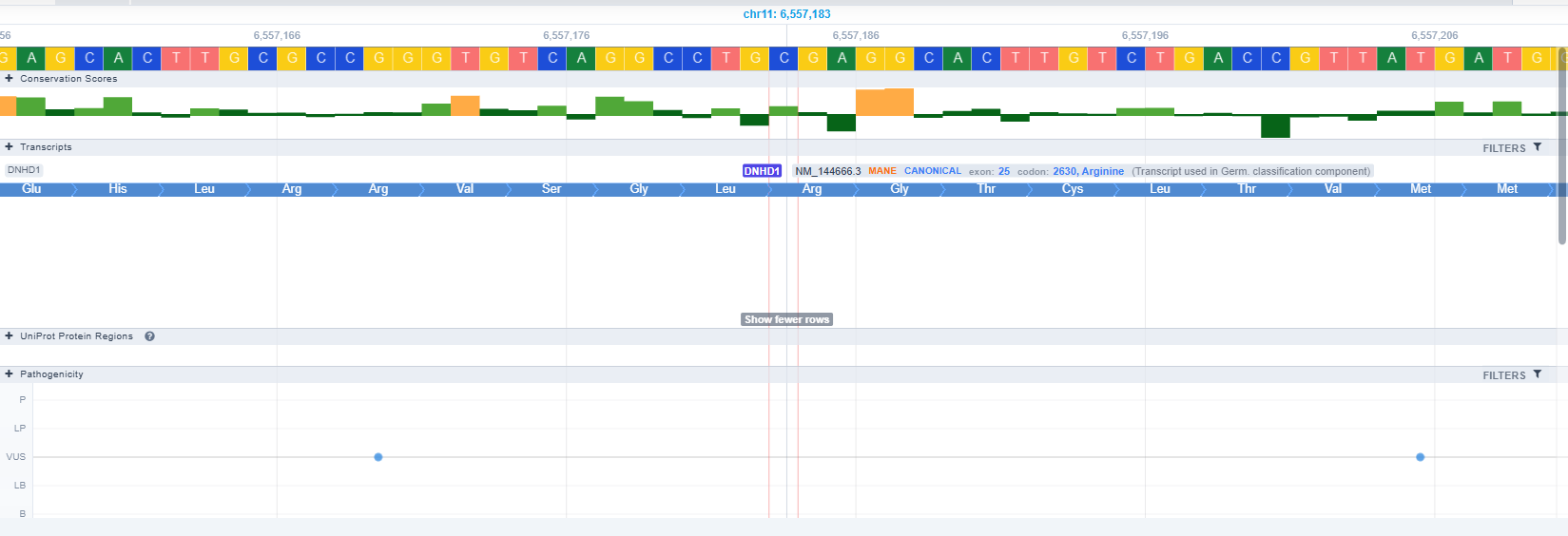
**

**Supp File 2. 5:** **a)** Case#4 pedigree ( +/+ Wild type, +/- Heterozygous for *ETAA1* gene variant), b) The c.2565_2568del (p.Lys855fs) frameshift deletion occurs at the C-terminal end of the *ETAA1* protein, specifically at position 855 of the full-length 856-amino-acid protein. (It is marked in red) (<https://pecan.stjude.cloud/variants/proteinpaint?gene=ETAA1>) c) The c.7888C>T (p.Arg2630*) stop-gain variant is located near the C-terminal region of the *DNHD1* protein, within the domain spanning approximately amino acids 2500–3000. This nonsense mutation introduces a premature stop codon at residue 2630, potentially resulting in truncated protein lacking essential downstream functional domains. ( <https://pecan.stjude.cloud/variants/proteinpaint?gene=DNHD1> ) (It is marked in red)

**Case Vignette#05 (NF Panel + WES)**

Case #05, who was born into a consanguineous marriage, was admitted to the Pediatric Hematology and Oncology Department of Medical Park Ankara Sehir Hospital and diagnosed with T cell lymphoblastic lymphoma at the age of 7. Three years later, she developed a primary AML exhibiting café-au-lait-like spots and presented astrocytoma in the brain. No translocation was found in cytogenetic tests. Due to the presence of café-au-lait-like spots, Neurofibromatosis (NF) was suspected, and an NF gene panel was performed at an external center, but yielded negative results. We enrolled the patient in the study according to Jongman criteria 1, 2, and 4 and performed whole exome sequencing. The index case showed a pathogenic homozygous ***MSH6* c.3934_3937dup p.Ile1313SerfsTer7** variant (ClinVar ID: 418610). We confirmed the variant in the buccal swab and her parents. All unaffected siblings except one was heterozygous; one of her siblings was homozygous (b.2018). Therefore, since the unaffected sibling was found to be heterozygous, transplantation planning was changed, and it was decided that the donor would be unrelated. Together with the genetic findings, the patient was diagnosed with CMMRD. Additionally, family members who were heterozygous for this variant were placed under surveillance due to the risk of Lynch Syndrome.

a)


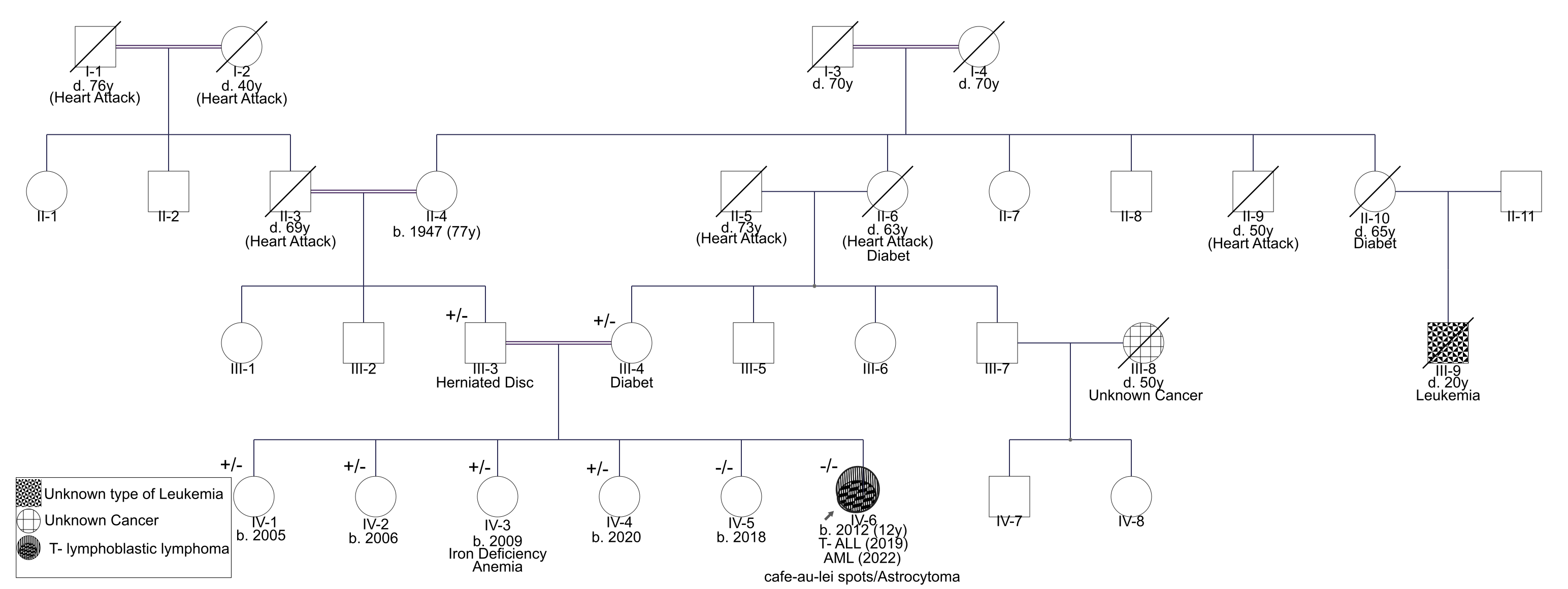


**b)  *MSH6* c.3934_3937dup p.Ile1313SerfsTer7**

**
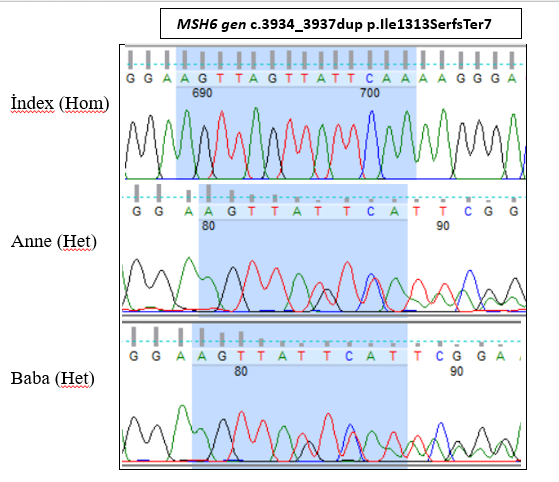

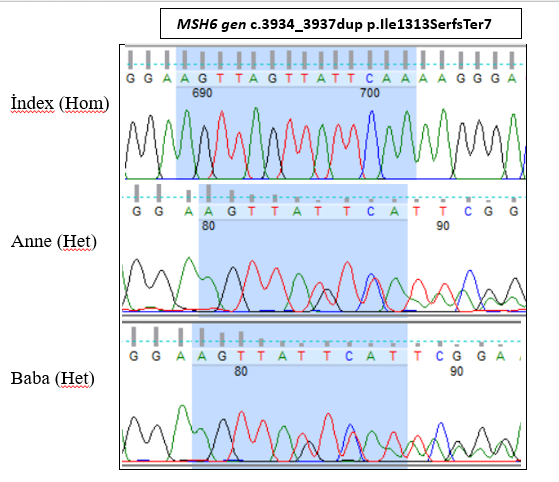
**

IV-6 III-4


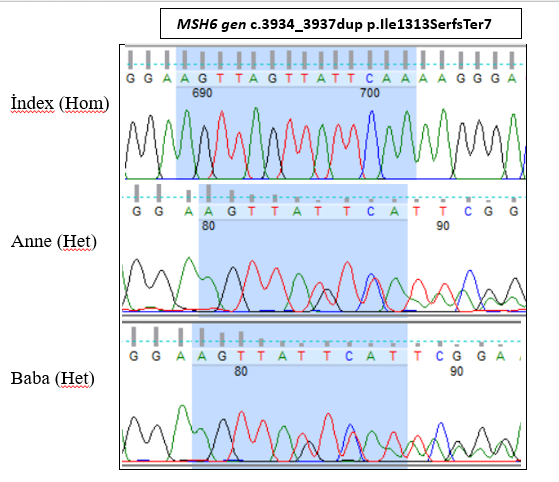


III-3

**Supp File 2. 6:** a) Case#5 pedigree (-/-Homozygous, +/- Heterozygous), b) Result of Sanger sequencing in the index case, mother and father

**Case Vignette #6 (WES)**

Case #6 was born in a consanguineous marriage and diagnosed with T-ALL at the age of 7. Her cytogenetic test revealed that she was t(12;21) and 5q deletion positive and was later diagnosed with bi-phenotypic leukemia. The patient's older brother was diagnosed with T-cell lymphoma when he was 5 years old and died at the age of 15. Peripheral blood and buccal swab samples were taken from the index. We reached a Formalin-fixed paraffin-embedded (FFPE) sample of the affected brother from the Marmara University Pendik Training and Research Hospital archive. The WES analysis of the index’s peripheral samples revealed two VUS and one likely pathogenic candidate variants. The first variation was a homozygous missense variant in ***ATR* gene *c.5273G>C p.Gly1758Ala*.** The variant has not been previously reported in the ClinVar database. It is classified as VUS (PM2, PP2, PP1) according to ACMG criteria. Sanger confirmed the homozygous gene variant in the index swab, blood tissue, and the FFPE tissue of the deceased affected sibling. The patient's mother, father, and unaffected siblings were heterozygous for this variant. Another candidate was a heterozygous ***NOTCH1* c.2929G>A p.Gly977Arg.** It is classified as LP (PP2, PP3, PM2) according to ACMG criteria. In segregation analysis, the variant was detected as heterozygous in the blood tissue of the patient, while it was observed as wild type in the intraoral swap tissue. Also, we detected the variant as heterozygous in the FFPE tissue of the deceased brother. While the variant was observed as heterozygous in the father, it was wild-type in the mother and unaffected siblings. The patient developed low-grade glioma during the study, so we also included the *BIRC6* variant as a candidate. The index was homozygous ***BIRC6* c.6124A>G p.Asn2042Asp** and classified as VUS (PM1, PM2). According to the Sanger result, the variant was detected as homozygous in the patient's blood tissue. While the variant was observed as WT in the father and one of the unaffected siblings, it was heterozygous in the mother and one of the unaffected siblings. The FFPE samples were insufficient for Sanger analysis, so we could not confirm the *BIRC6* variant of the deceased brother. From a clinical perspective, considering the unaffected sibling who would be the donor was found to be heterozygous, the decision was made to forgo the transplantation and opt for an unrelated donor.

a)


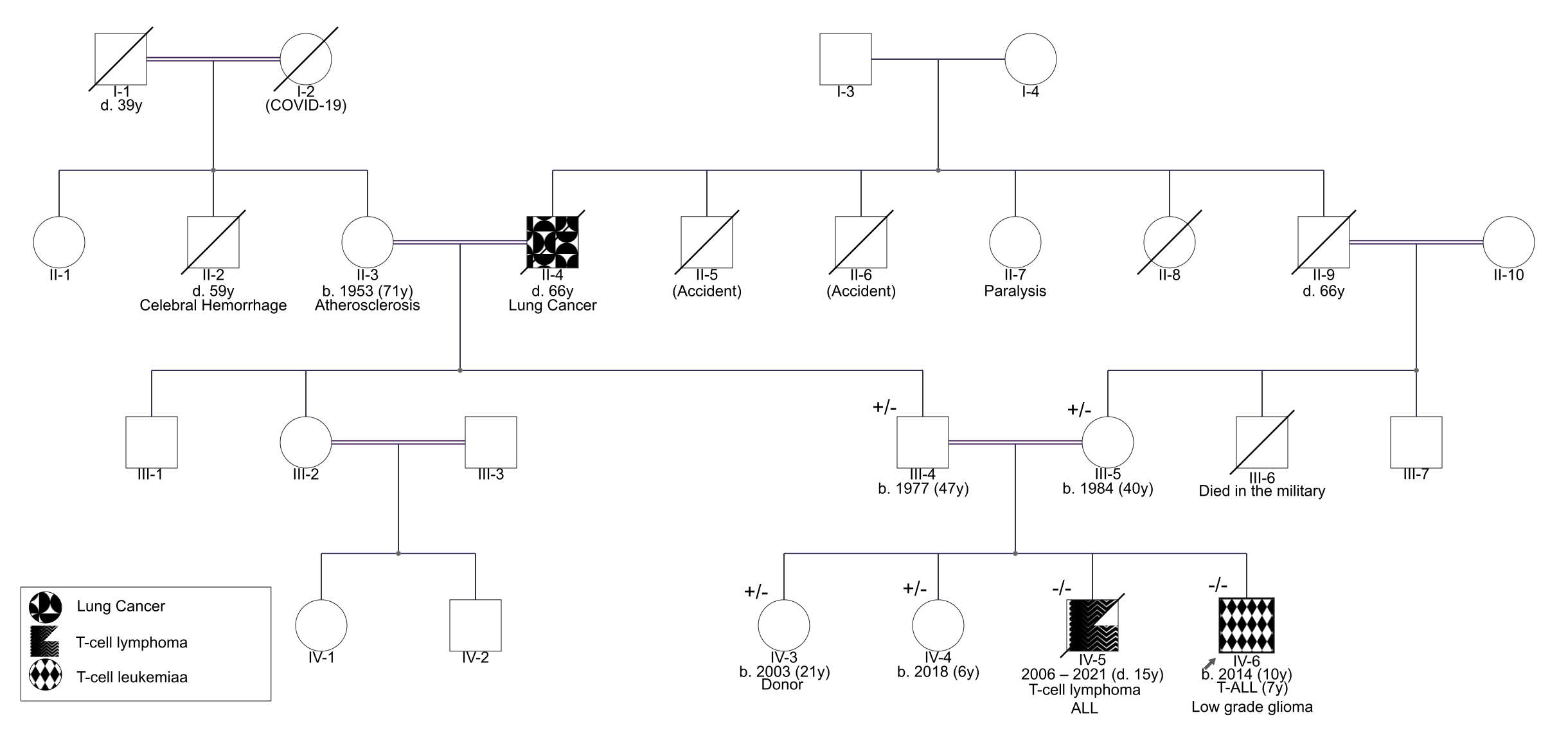


b) ***ATR* gene *c.5273G>C p.Gly1758Ala***


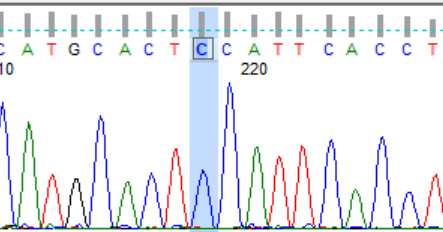

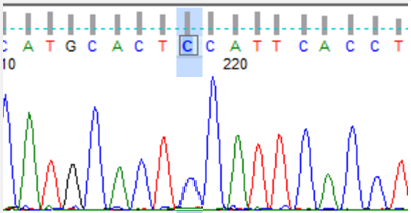


IV-5 IV-6


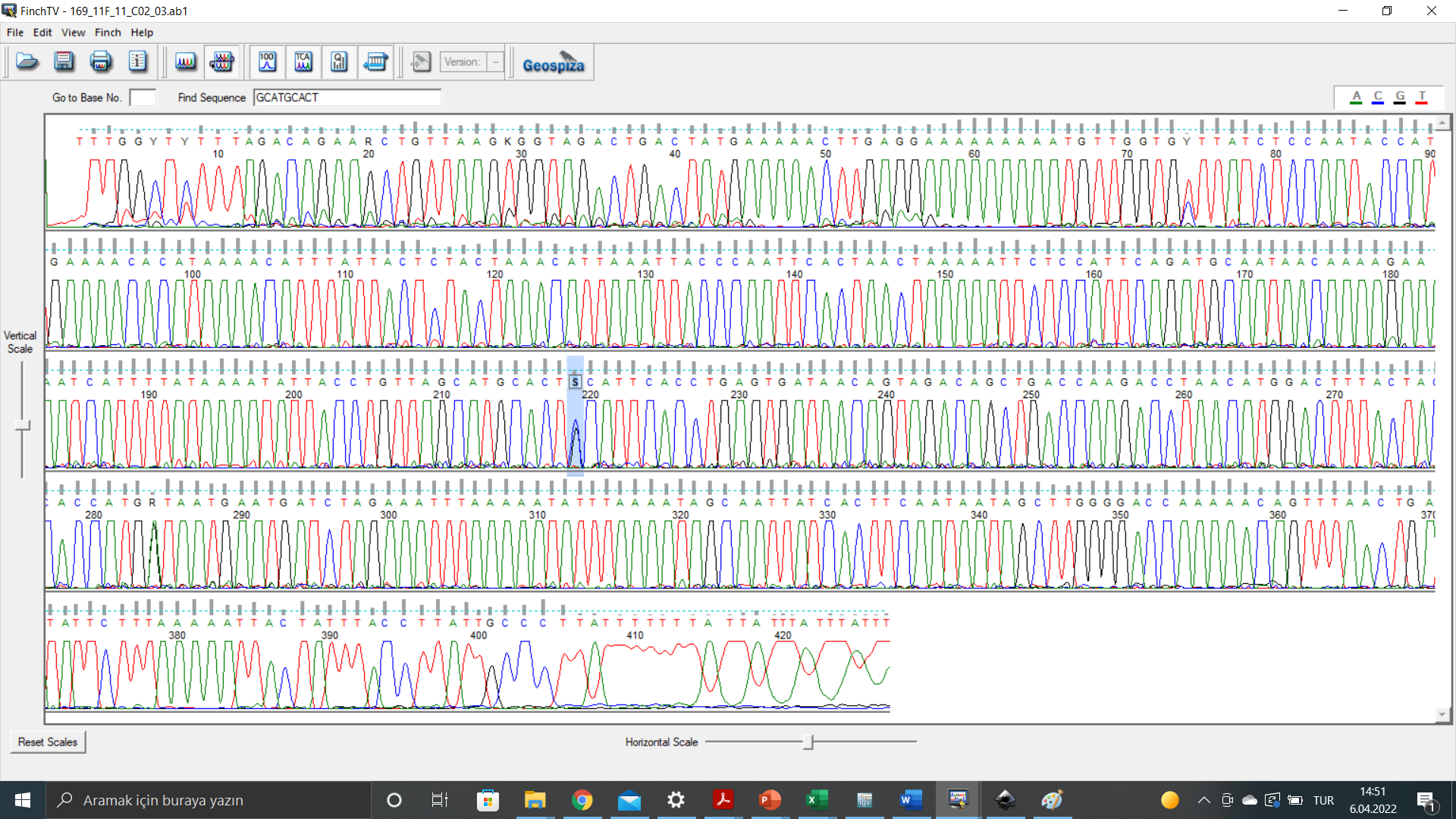

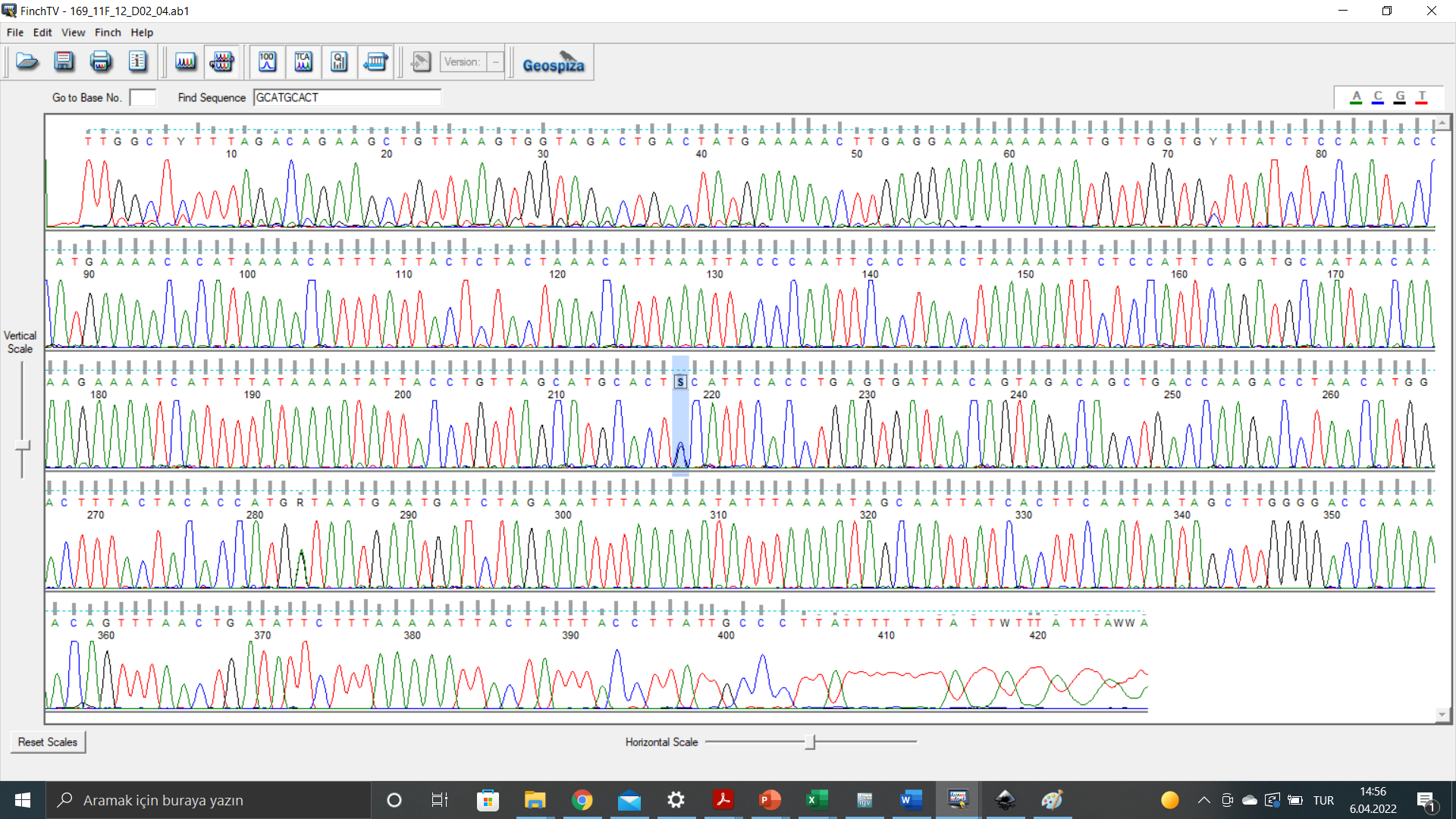


III-4 III-5

c)


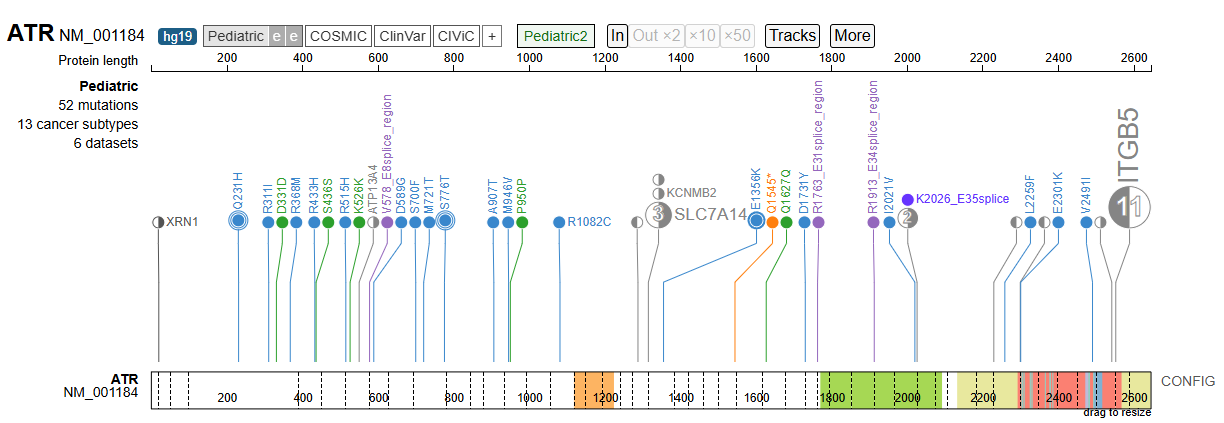


**
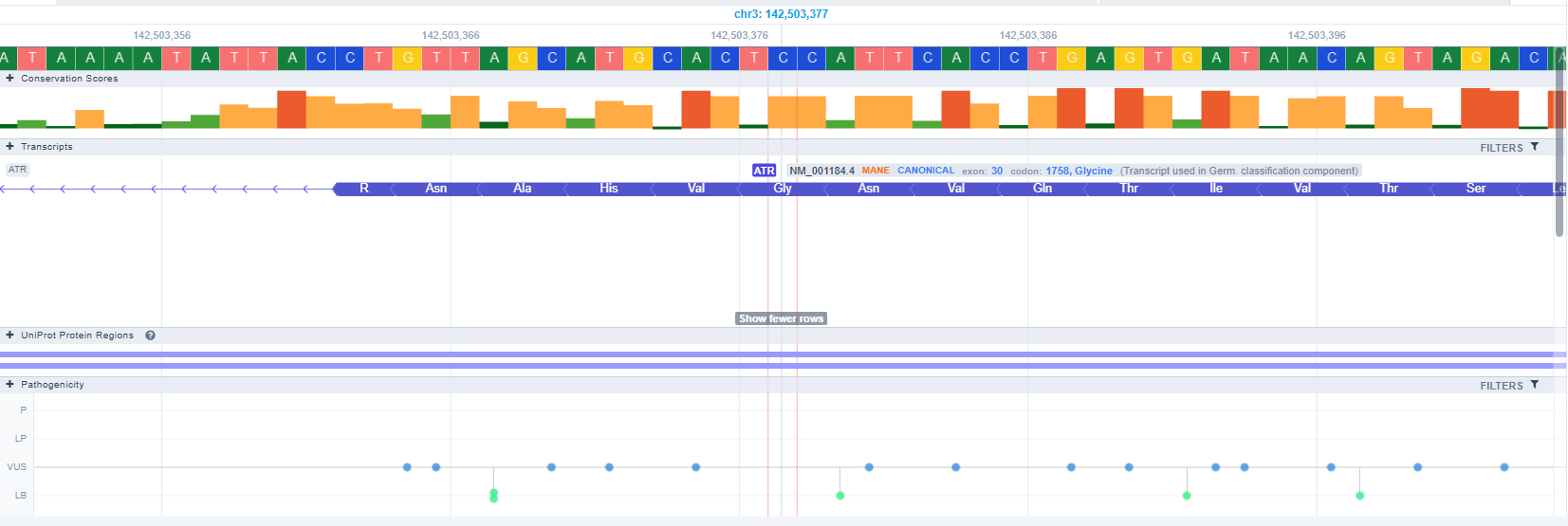
**

Supp File 2. 7: a) Case#6 Pedigree (-/-Homozygous, +/- Heterozygous for *ATR* gene variant ), b) Result of Sanger sequencing in the index case, affected brother, father and mother, c) *ATR* gene variant is located at amino acid position 1758, which corresponds to a critical kinase domain of the protein. This region is essential for *ATR’s* role in the DNA damage response pathway, and alterations within this domain may impair its kinase activity, potentially leading to genomic instability and increased cancer susceptibility (<https://pecan.stjude.cloud/variants/proteinpaint?gene=ATR> ) (It is marked in red).

**Case Vignette #7 (WES)**

Case #7 was admitted to the clinic due to an enlarged lymph node and later diagnosed with B-ALL at the age of 12. His parents were distantly related, and his mother was diagnosed with Hodgkin Lymphoma at the age of 45. The patient showed severe infections and drug resistance. We enrolled the patient with the Jongman criteria 1 and 5 and performed whole exome sequencing. The WES revealed a heterozygous missense variant ***WRN gene* c.1721G>T p.Gly574Val.** The variant was classified as VUS in ClinVar (ClinVar ID: 933329). However, the variant was at the first base of the acceptor site, and we classified it as pathogenic, with strong evidence attributions in PP3, PP5, PM1, and PM2. The segregation analysis determined the variant as heterozygous in the index, the affected mother, and the unaffected sister. The unaffected father was WT. The patient was treated with bone marrow transplantation from an unrelated donor.


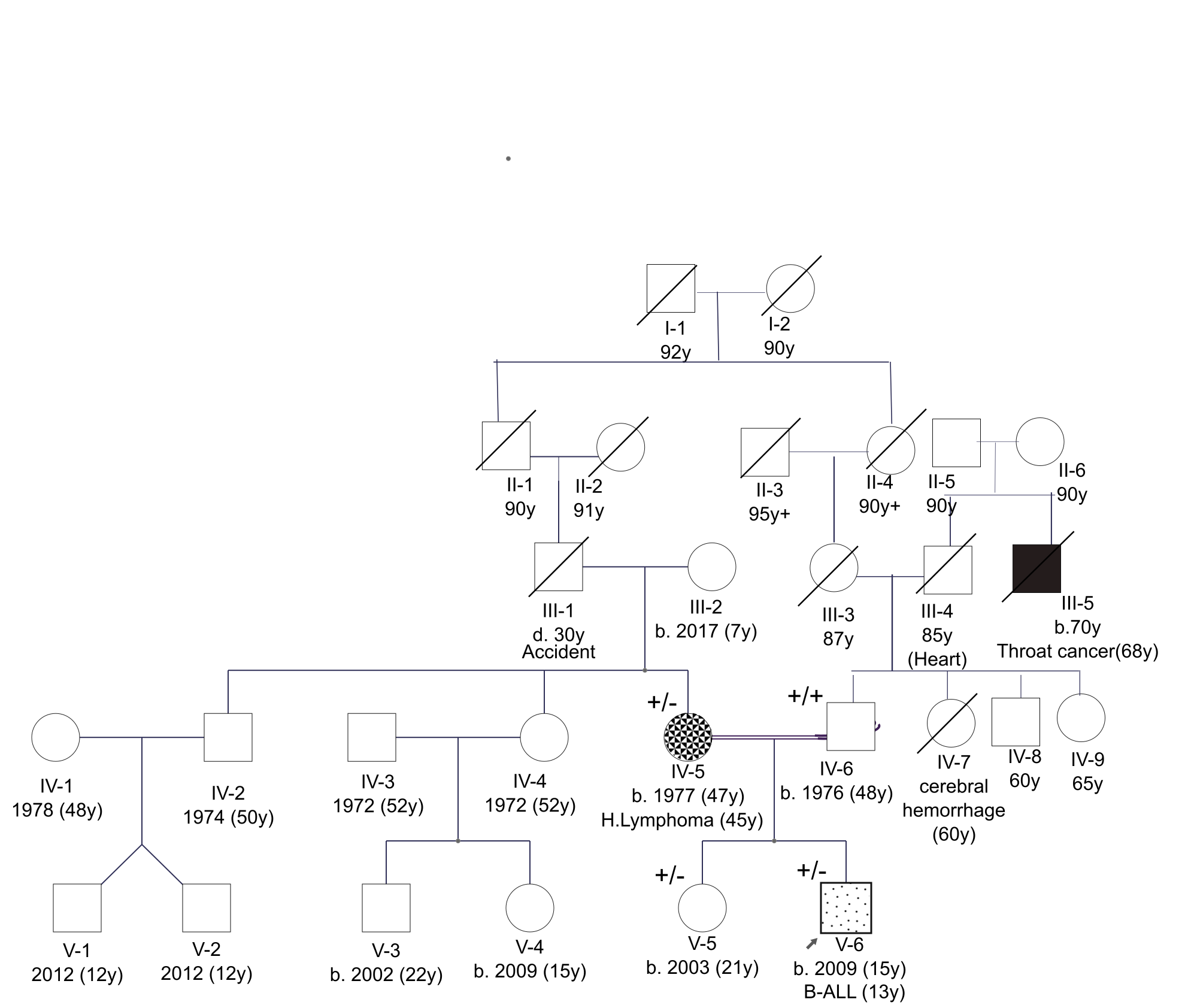


**
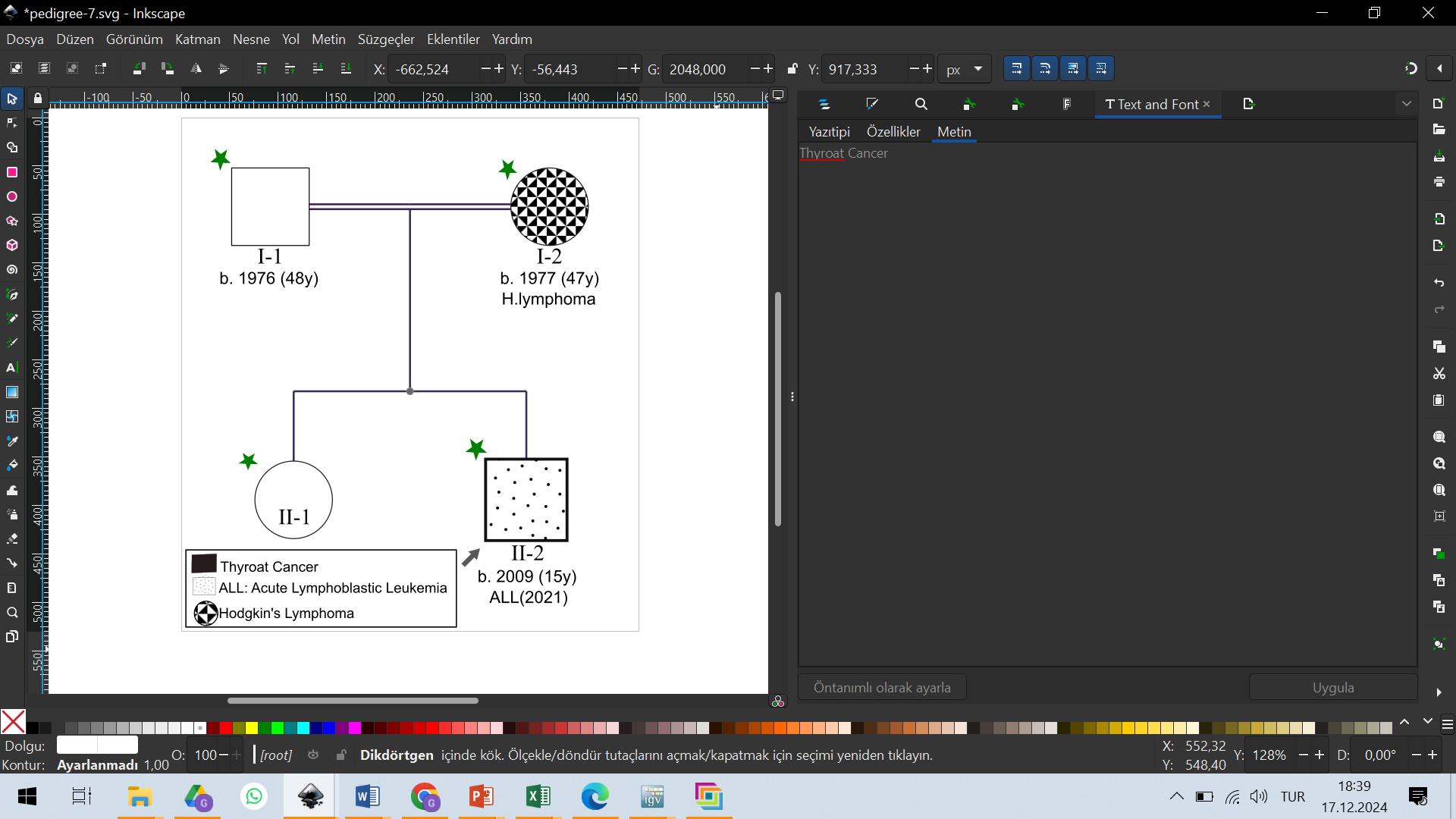
**

**Supp File 2. 8:** Case#7 pedigree (+/+ Wild Type, +/- Heterozygous)

**Case Vignette #8 (WES)**

Case #8 was diagnosed with B-ALL at the age of 2. Index's father was diagnosed with AML at the age of 44, and he was responsive to treatment. One of the paternal uncles of the father died due to lymphoma, and the other died due to osteosarcoma. With cancer presentation in 3 generations, we enrolled the patients in the study with Jongman criteria 1. The WES analysis determined a heterozygous ***MYH11 c.1291C>T p.Arg431Cys***  variant, classified as LP (PM1, PM2, PP2, PP3) according to ACMG criteria. The variant has not been previously reported in the ClinVar database. Sanger sequencing confirmed it both in the index case and the affected father. Mother and unaffected sibling were wild type.


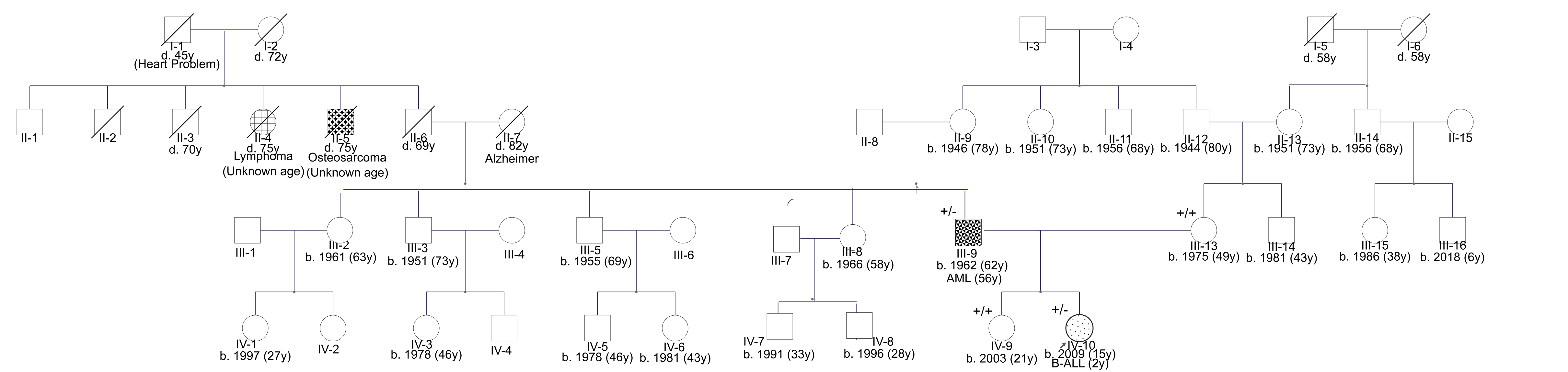


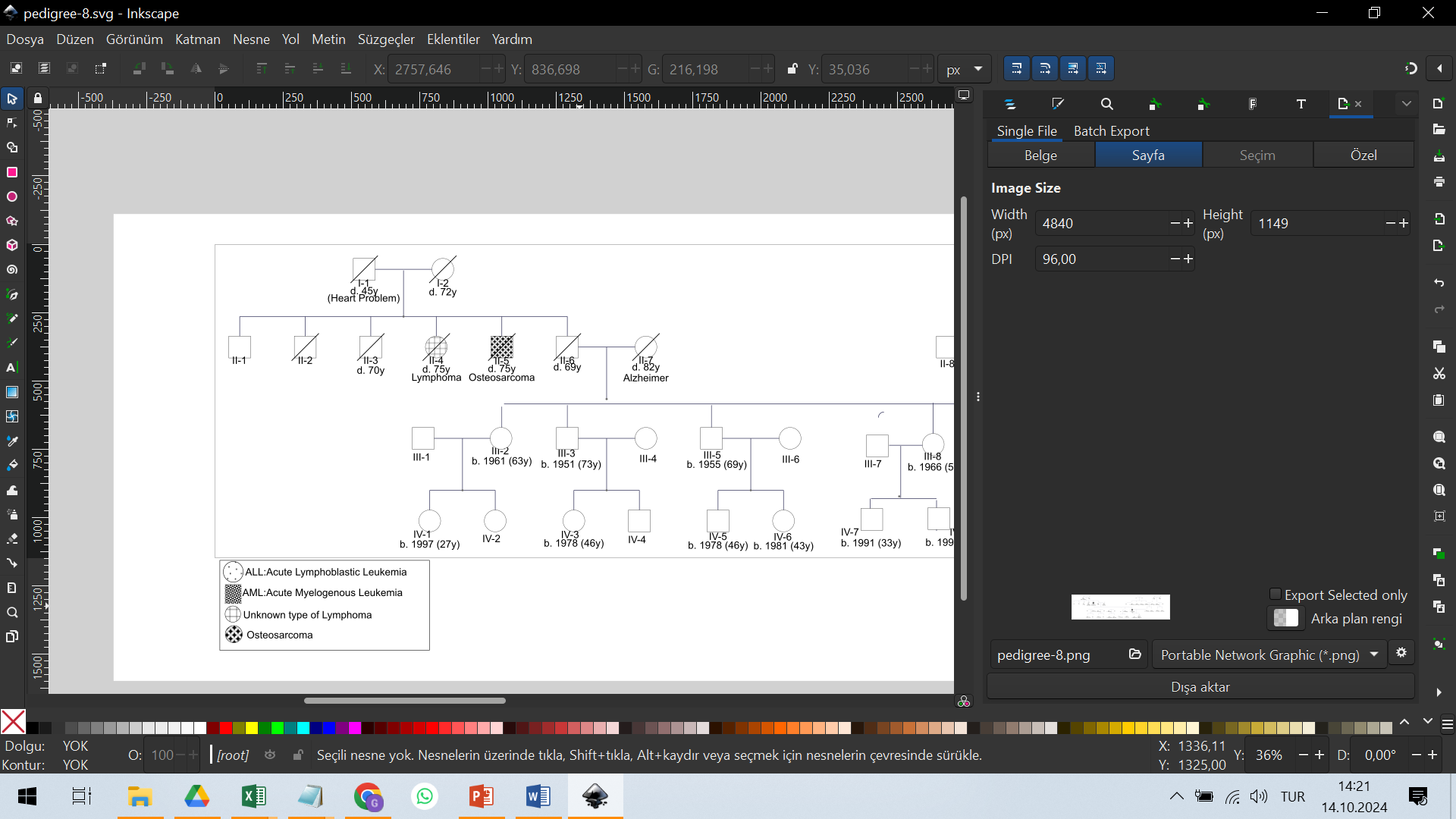


**Supp File 2. 9:** Case#8 pedigree (+/+ Wild Type, +/- Heterozygous)

**Case Vignette#9 (WES)**

The index case is 13 years old, and she was diagnosed with B-ALL when she was 8 years old. Her mother died of lung cancer at the age of 27; her maternal uncle died due to brain cancer at an unknown age. Another maternal uncle was also diagnosed with kidney cancer, and her maternal grandfather died due to lung cancer at an unknown age. Her father was diagnosed with Schızophrenia, her paternal aunt was the guardian, and she mentioned that the family was not very cooperative. Hence, no material or detailed information could be obtained from the maternal side. The Jongman criteria one was applied and the patient was enrolled in the study. The WES analysis performed in the peripheral blood sample of the index case detected a heterozygous ***JAK2 gene c.2047A>G:p.R683G.*** (**ClinVar ID:** 375951). The variant was classified as pathogenic/likely pathogenic in ClinVar, and we classified it as pathogenic with the evidence attributions of PM5, PM1, PP3, PM2, PP5. Candidate variants were validated by Sanger sequencing.


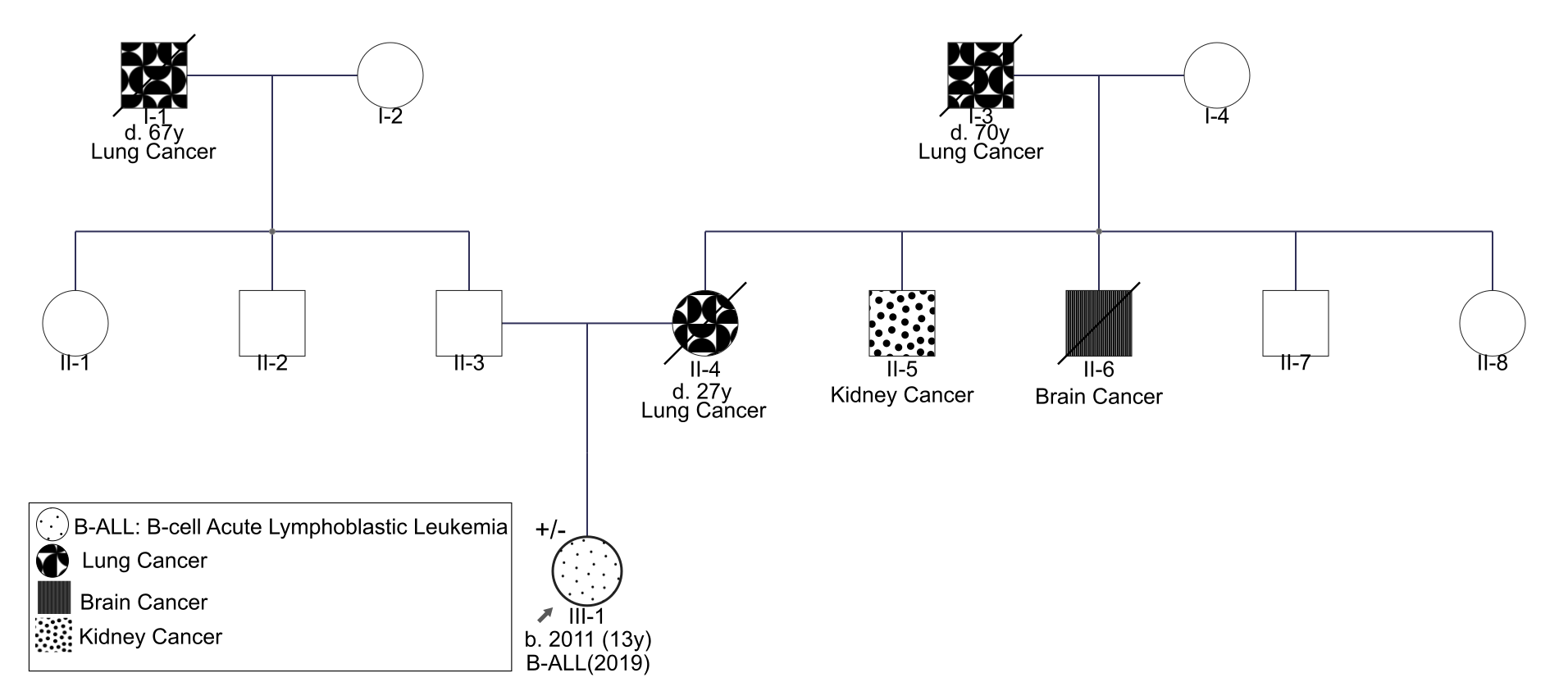


**Supp File 2. 10:** Case#9 pedigree (+/- Heterozygous)

**Case Vignette#10 (WES)**

Case #10 was diagnosed with B-ALL at the age of two. There is no known consanguinity between the parents. His mother died of ovarian cancer at the age of 30, and his grandfather died of lung cancer at the age of 60. Only the sample of the index case was available, and we determined a heterozygous stop gain variant of ***JAK3* gene c.3247C>T:p.Q1083X.** The variant has not been previously reported in the ClinVar database.


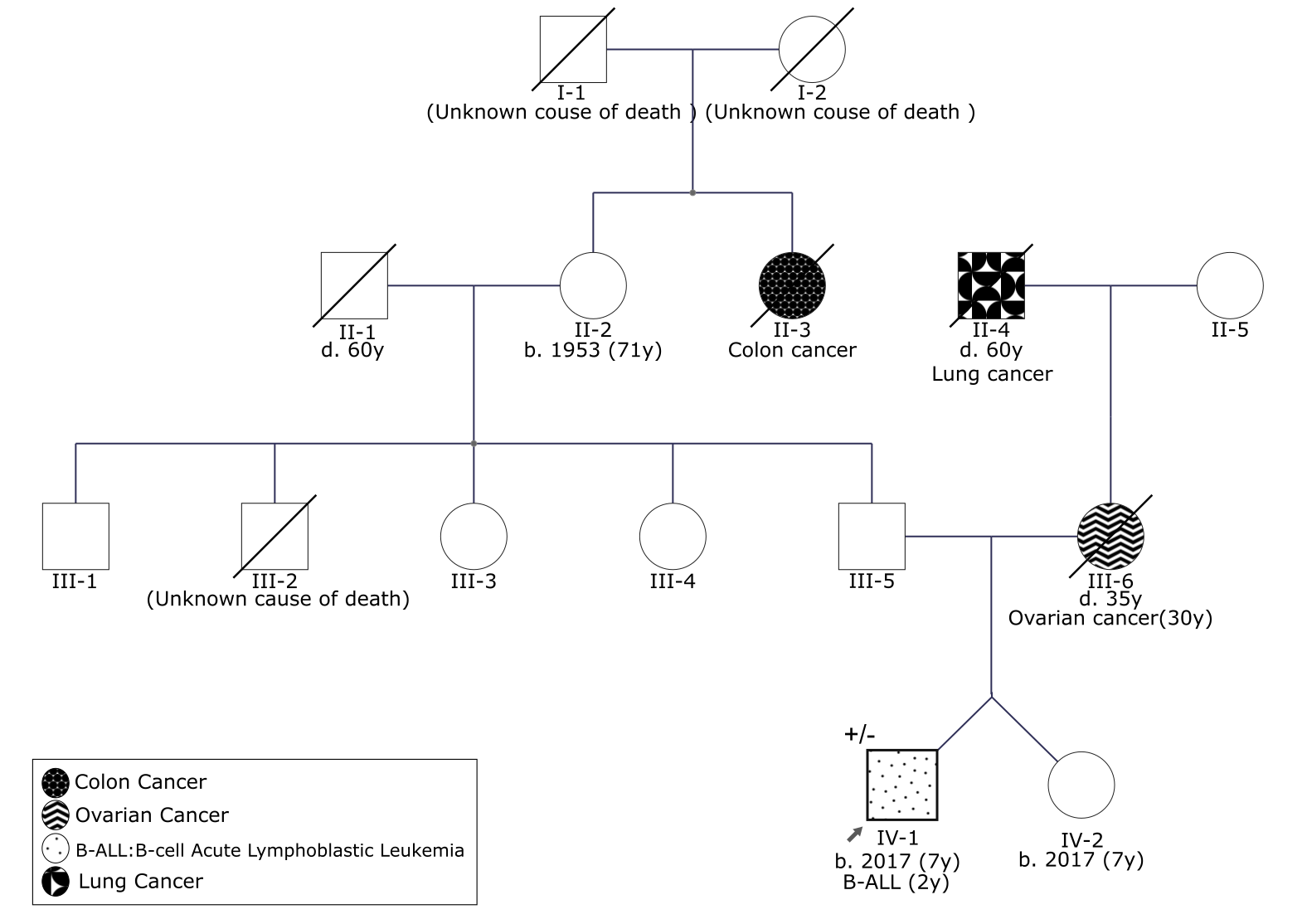


**Supp File 2. 11:** Case#10 pedigree (+/- Heterozygous)

**Case Vignette #11 (WES)**

Case #11 was born into a consanguinous marriage and diagnosed with B-ALL when she was 12 years old. The cytogenetic test result showed hypodiploidy and t(12;21). The index's mother was diagnosed with colon cancer at the age of 36 and her father’s maternal uncle was diagnosed with bladder cancer at an unknown age. The physician also reported treatment toxicity in the index case. We enrolled the patient in the study according to the Jongman criteria 1, 2, 4 and 5 and performed WES analysis. The results revealed two candidate variants: First was a heterozygous stopgain variant in ***MVP* gene c.2296C>T p.R766X.** The variant has not been previously reported in the ClinVar database. It is classified as LP (PM2, PP2, PP3) according to ACMG criteria. The second variant was a heterozygous frameshift insertion ***IL32* gene c.515_516insG p.Asp172Glufs***. This was classified as VUS with the PM2 and PP1 evidence attribution. Both variants were confirmed in the index case and the affected mother.


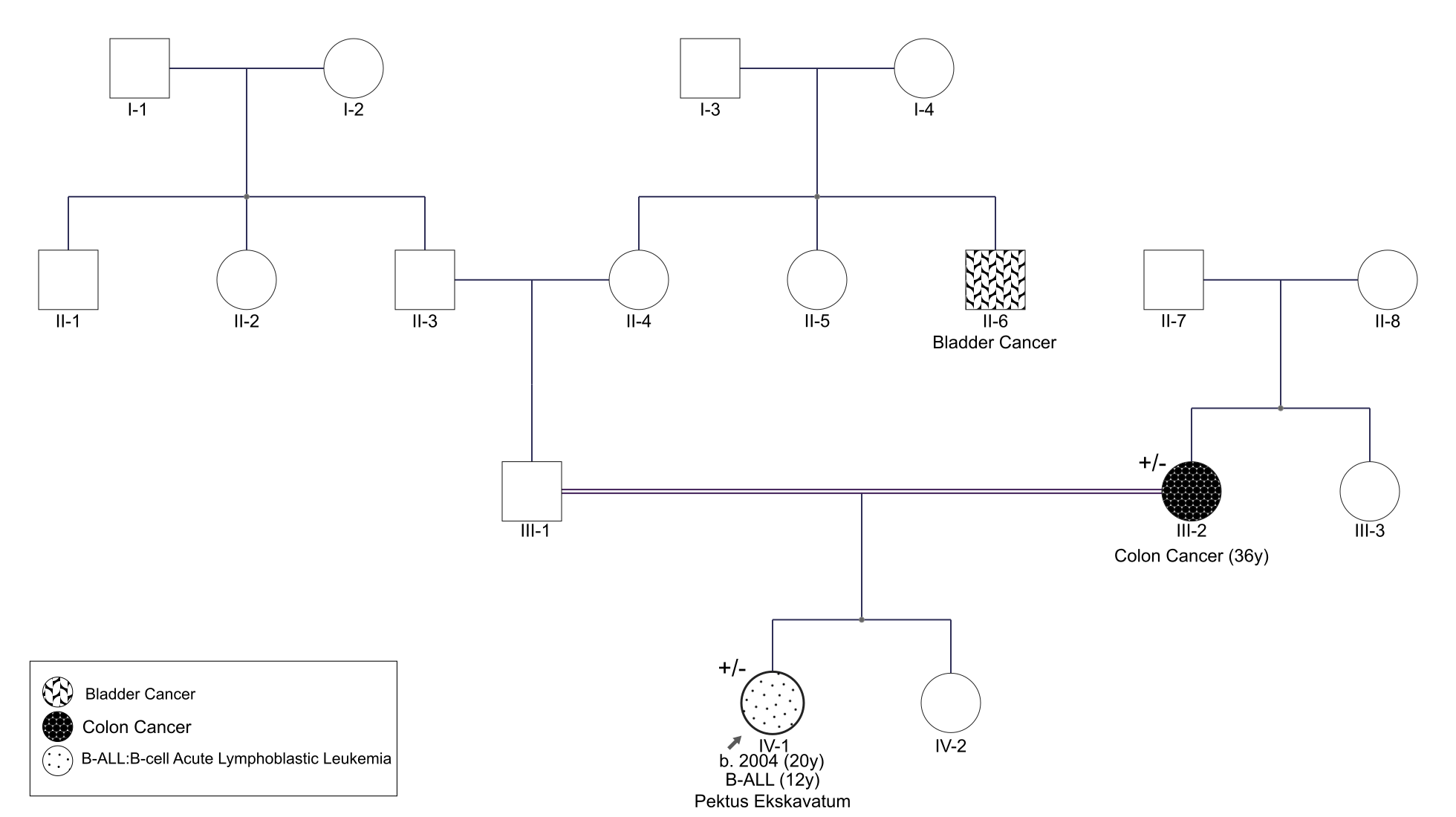


**Supp File 2. 12:** Case#11 pedigree (+/- Heterozygous for *MVP and IL32* gene variant)

**Case Vignette #12 (Panel + WES)**

Case #12 was diagnosed with Hodgkin Lymphoma when he was 11 years old. There is no known consanguinity between his parents, and his mother was diagnosed with breast cancer at the age of 40. The mother’s paternal uncle died due to lung cancer at the age of 60. A targeted panel sequencing was performed on the index at an external center, and no causative variant was detected. We enrolled the patient with Jongman criteria 1, and WES analysis revealed a heterozygous missense variant in ***RAD52* gene c.388G>A p.Glu130Lys**. The variant has not been previously reported in the ClinVar database. It is classified as VUS (PM2) according to ACMG criteria. The mother refused to provide a sample for segregation analysis, and the index sample was confirmed by Sanger sequencing.


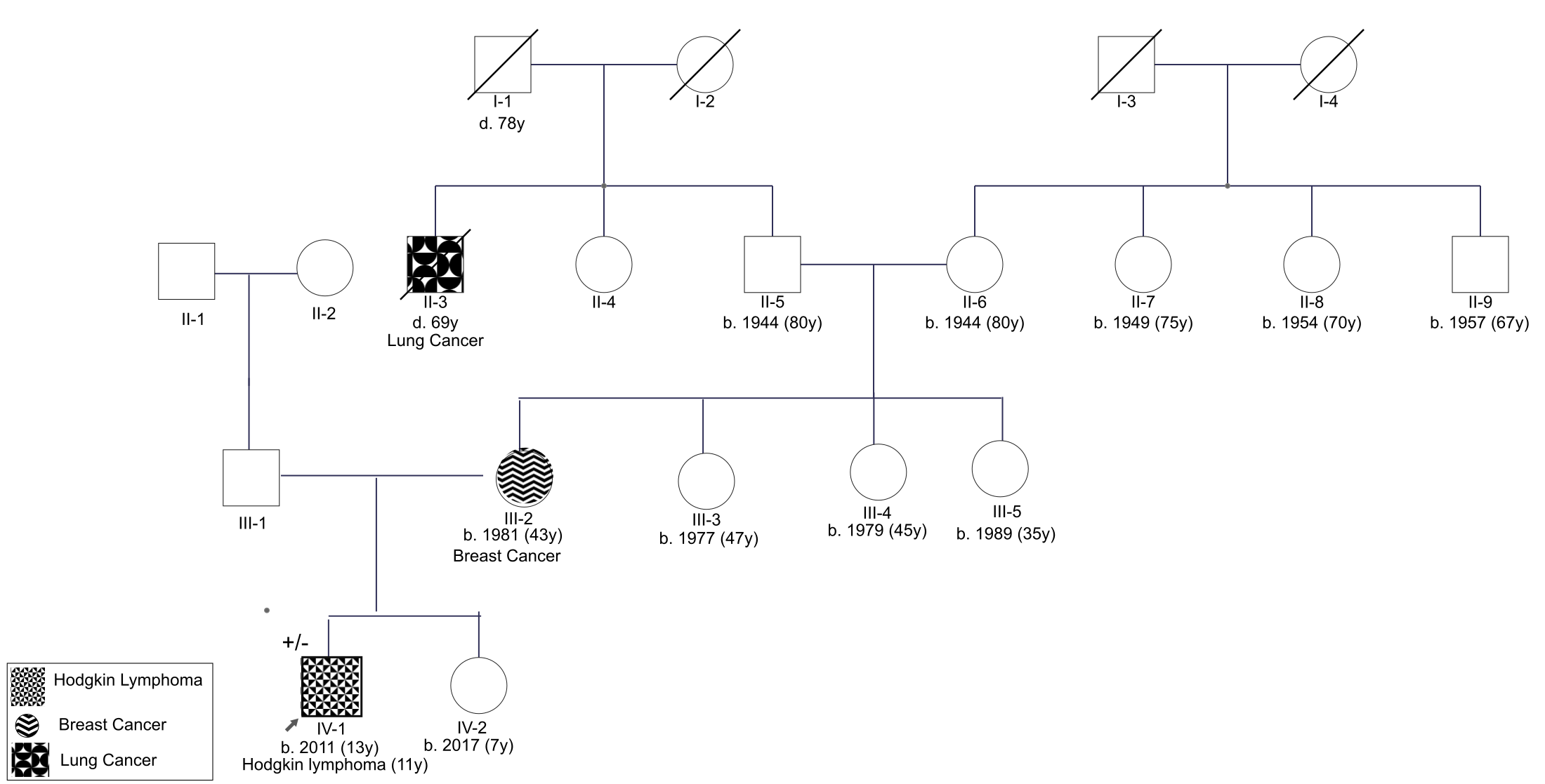


**Supp File 2. 13:** Case#12 pedigree (+/- Heterozygous)

**Case Vignette #13 (CES + WGS+RNA seq)**

Case #13 was born into a consanguinous marriage and diagnosed with Hodgkin Lymphoma at the age of 13. He had a younger brother who was diagnosed with Hodgkin Lymphoma at the age of 8. The father and the two paternal uncles were also diagnosed with Hodgkin Lymphoma at the ages younger than 30. The family was run by clinical exome sequencing at an external center, and no candidate variant was detected. Hence, we enrolled the index case, his affected sibling, his affected father, and his unaffected mother in the WGS analysis. The joint analyses revealed a homozygous intronic variant ***TNFRSF9* c.413+6T>C** in all affected cases, and the mother was heterozygous. The variant has not been previously reported in the ClinVar database and classified as Likely Pathogenic (PP3, PM2, PP1) according to ACMG criteria. Since the variant was in the intronic region and close to the splice site, we also performed RNA sequencing to analyze the consequences on the RNA level. The splice AI tool revealed that the variant creates an alternative splice site between exon 5 and 6. The variant was also confirmed to be homozygous in the affected uncle's blood tissue. Additionally, the variant was confirmed in the buccal swab of the index case. Functional analysis of this variation is ongoing.

a)


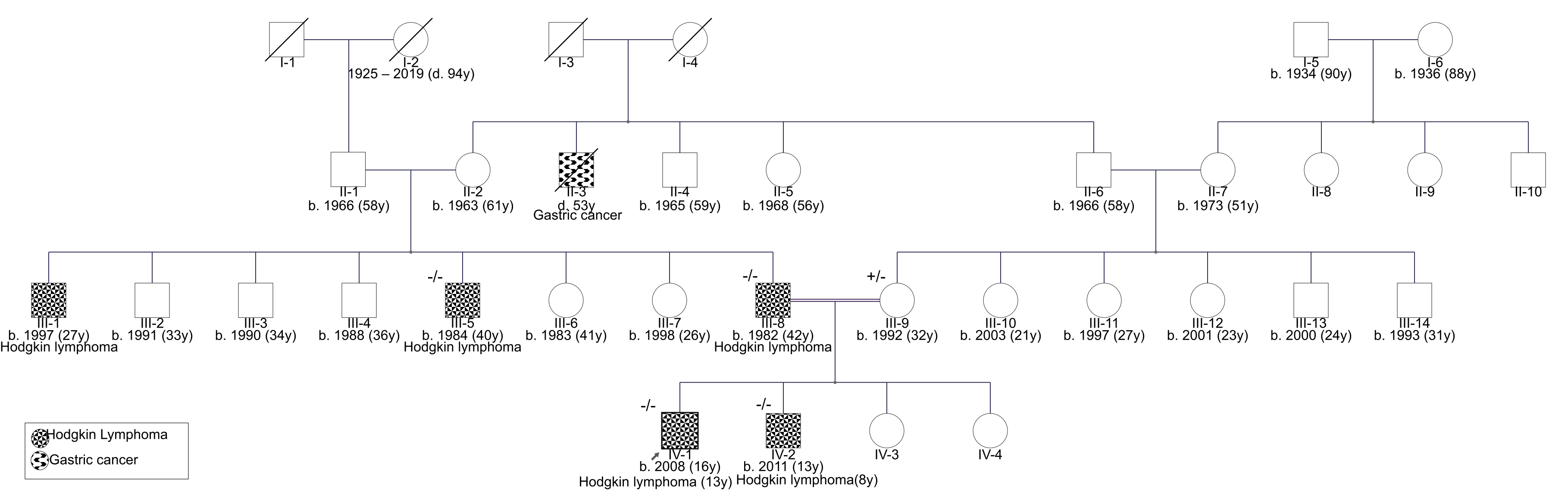


**b) *TNFRSF9* c.413+6T>C**

**
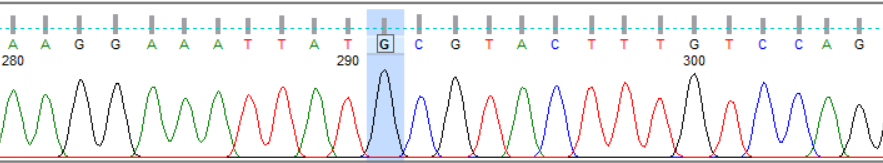

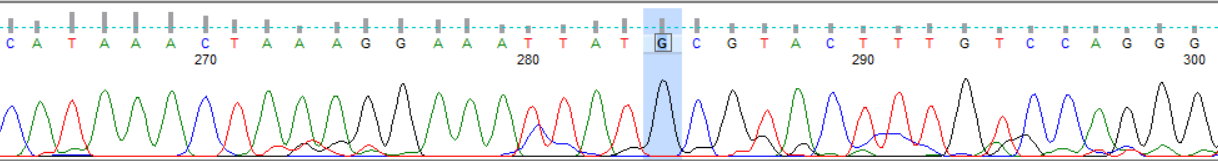
**

**IV-1 IV-2**

**
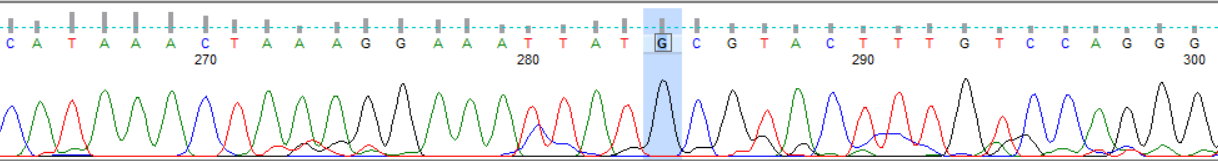

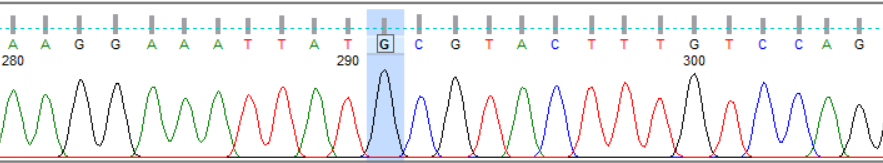
**

**III-8 III-5**

c)


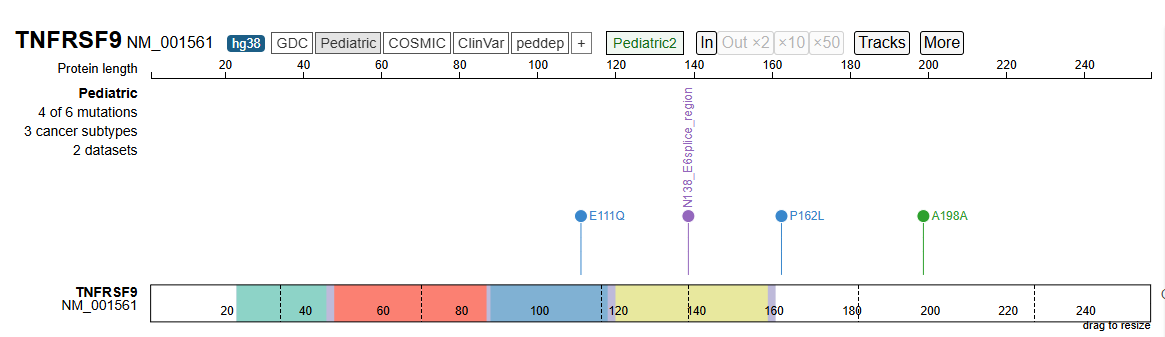


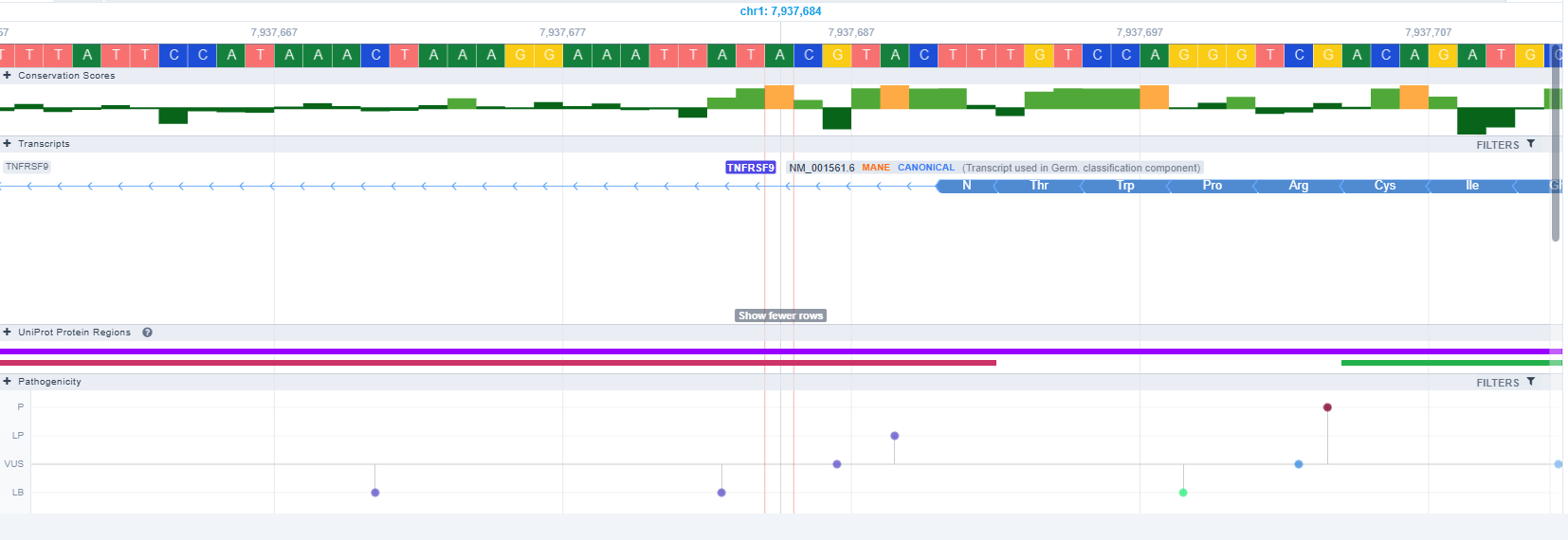


**Supp File 2. 14:** Case#13 pedigree (-/- Homozygous, +/- Heterozygous). b) Result of Sanger Sequencing in the index case, affected brother, father and uncle, c) The c.413+6T>C variant in the *TNFRSF9* gene is an intronic substitution located 6 base pairs, within the donor splice site region. It is located within the CRD3 [structural motif]. (<https://pecan.stjude.cloud/variants/proteinpaint?gene=TNFRSF9> ) (It is marked in red.)

**
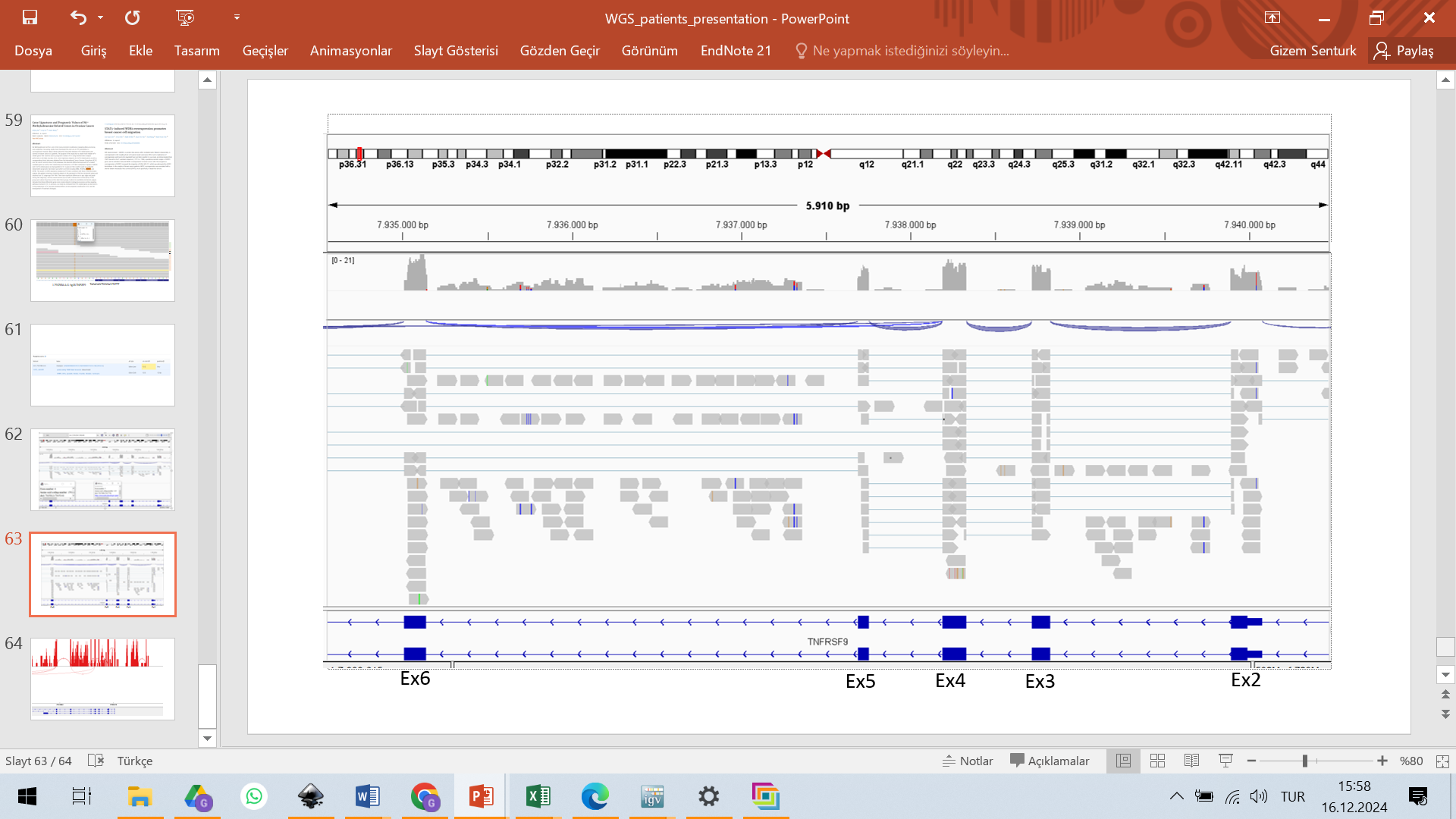
**

**
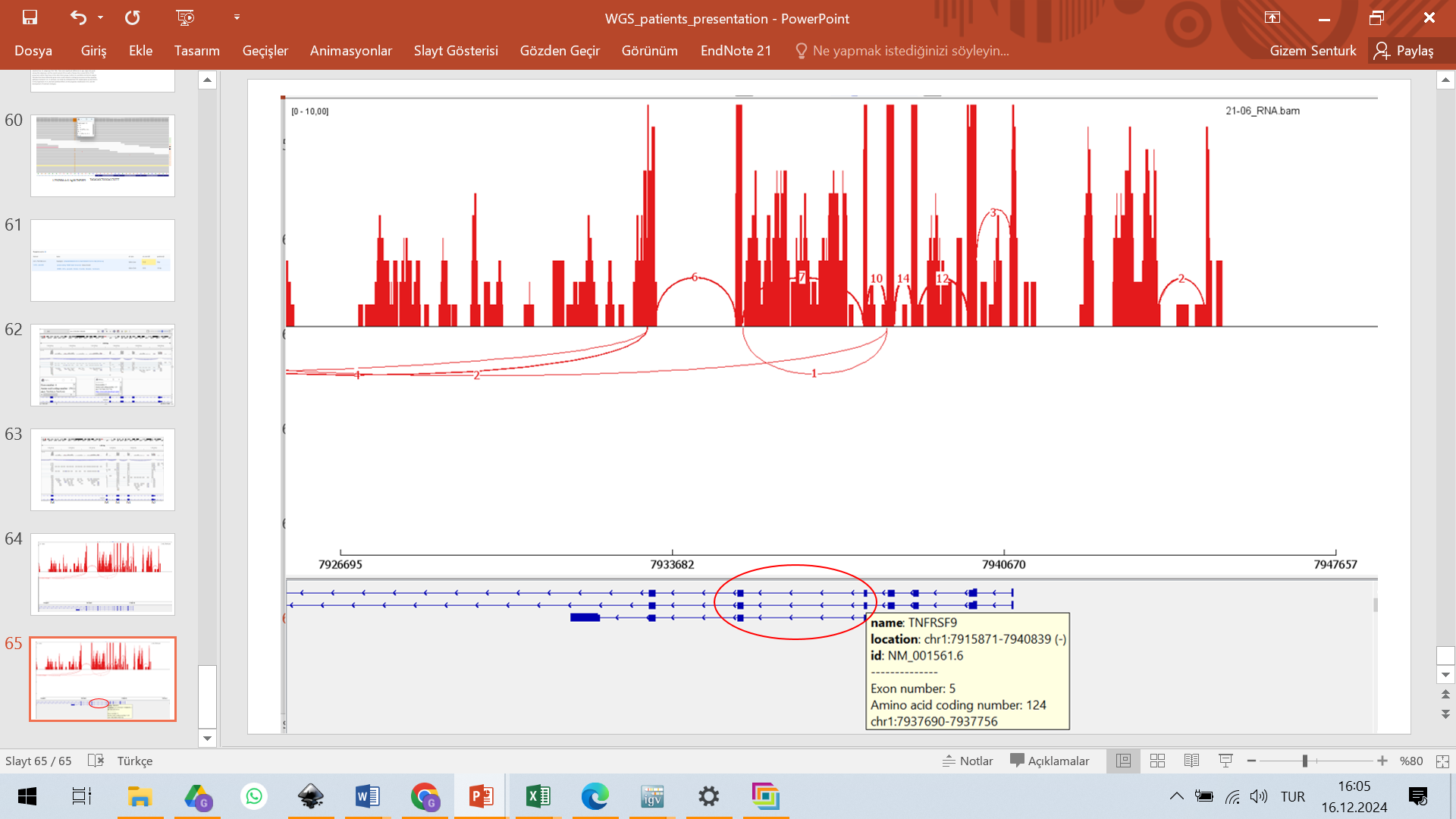
**

**Supp File 2. 15:**  IGV image from RNA seq .bam file


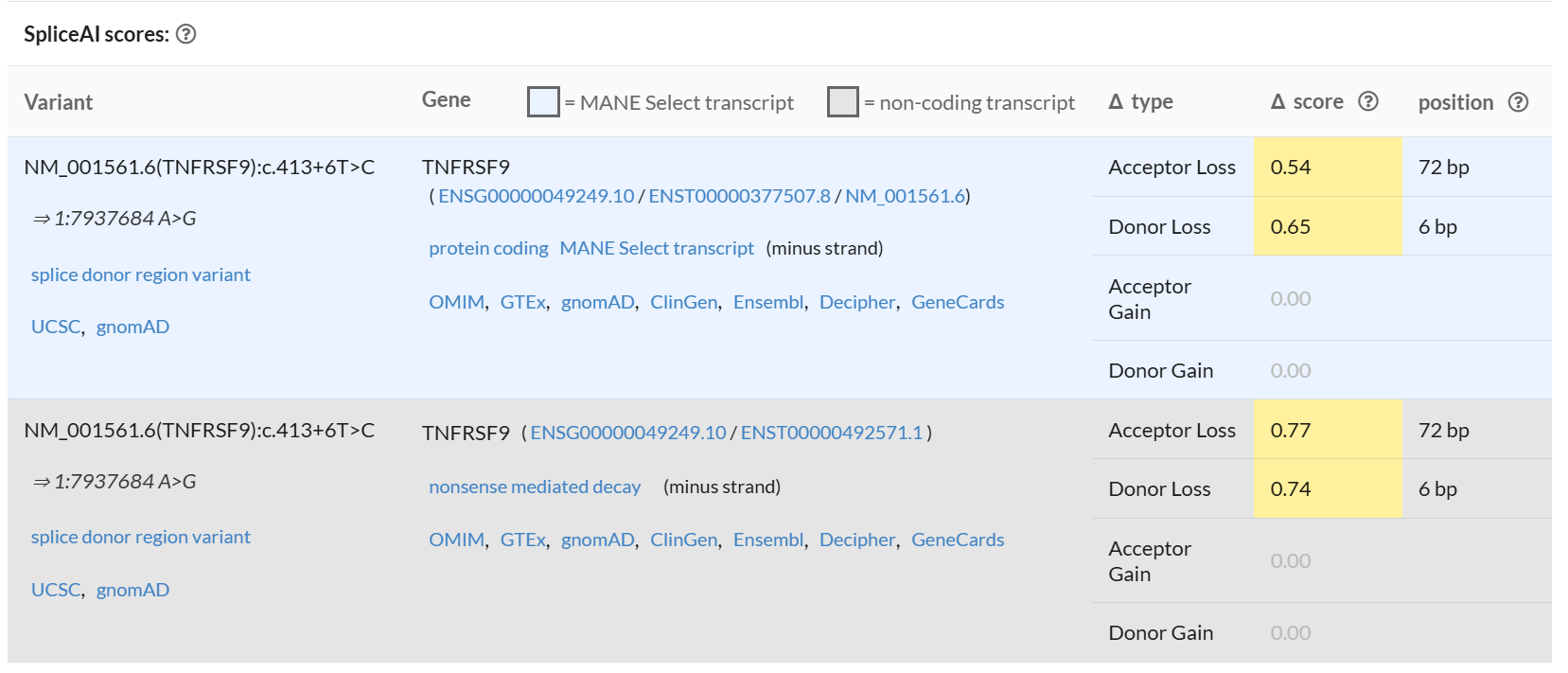


**Supp File 2. 16:** SpliceAI score of *TNFRSF9* gene variant

**Case Vignette #14 (WES + WGS)**

Case #14 was diagnosed with B-ALL at the age of 4. Soon after, he developed renal carcinoma and a brain tumor. The parents declared no consanguinity, but they were from the same town. With the findings of three primary tumors, we enrolled the index using the Jongman criteria: 1. The WES analysis detected three candidate gene variants associated with the clinical condition. These gene variants were heterozygous ***RECQL c.603G>T p.Met201Ile, TGFBRS1 c.1036A>Cp.Thr346Pro, PAPSS2 c.1753C>T p.Arg585Ter***. All three variants were heterozygous in the twin brother and the mother, whereas WT in the father and unaffected brother. Following the detection of these gene variants, the patient died at 5. With the decision of the cancer predisposition council, the samples of the mother, father, monozygotic twin, and unaffected sibling were run by WGS for joint analysis. After WGS, clinically associated heterozygous splicing variant *MLLT10* c.1700-6C>G, and heterozygous splicing variant ***BCNP1/NIBAN3* c.1554+1G>T** were identified in the index case. The variants have not been previously reported in the ClinVar database. *MLLT10* gene variant was classified as VUS (PM2). The *MLL10* variant was wild type in the father and twin brother, while it was heterozygous in the mother and the other sibling. The *BCNP1* gene variant was classified as VUS (PM2, PP1) and was heterozygous in the twin brother and the father, while it was wild type in the mother and the other sibling.


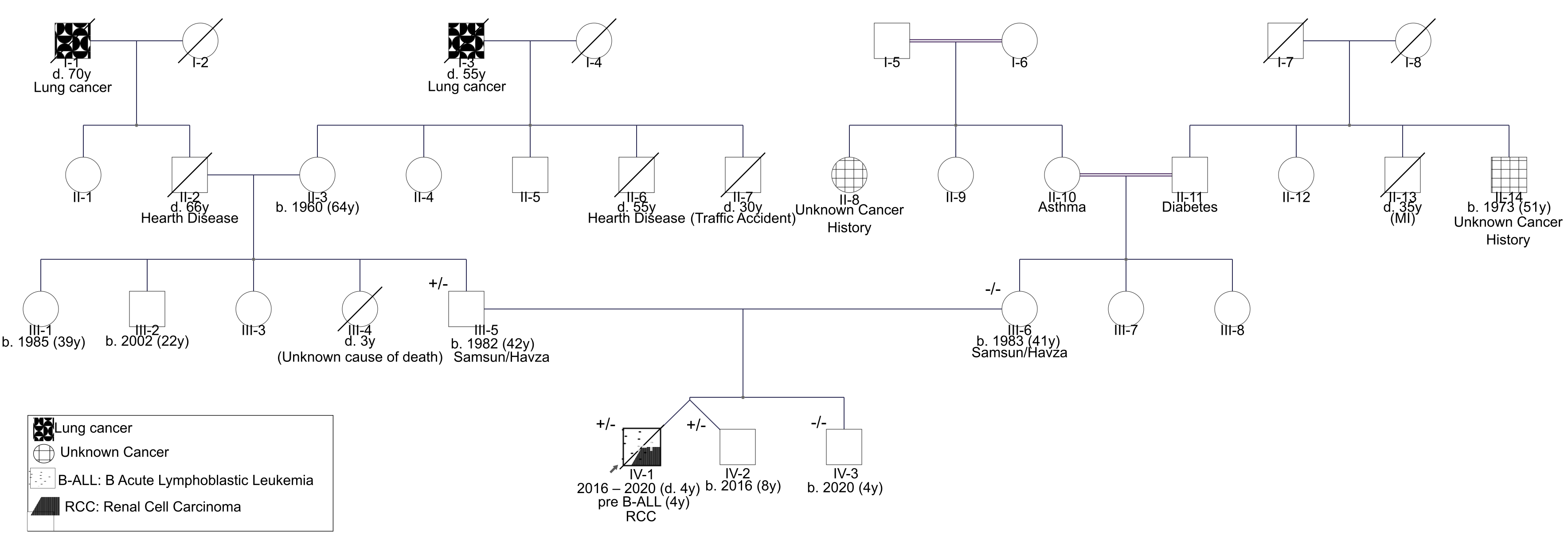


**Supp File 2. 17:** Case#14 pedigree (+/+Wild Type, +/- Heterozygous for *BCNP1* gene variant)

a)


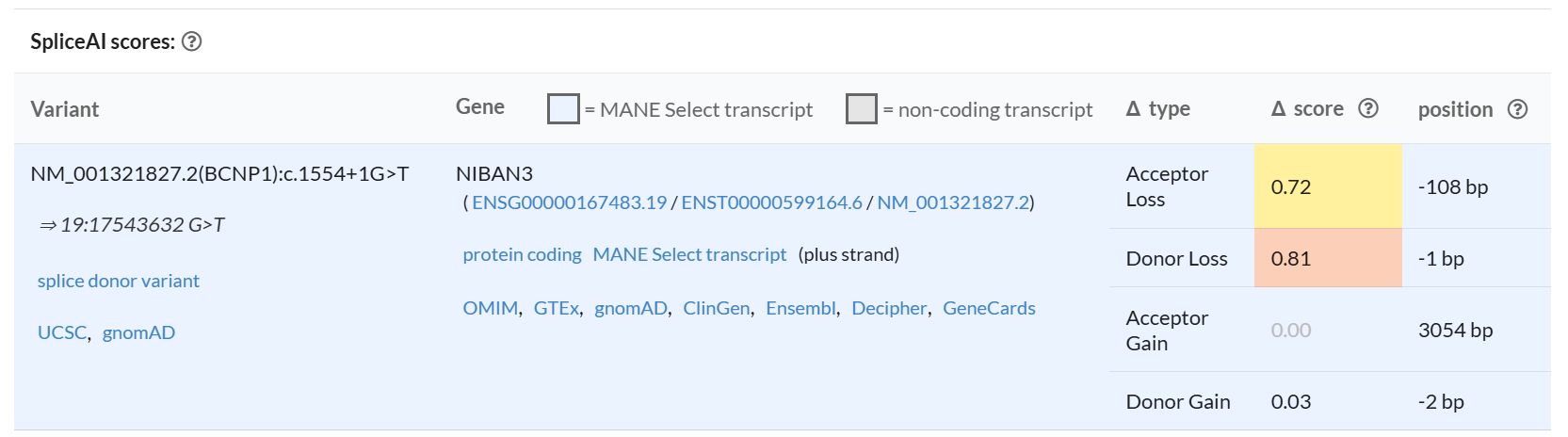


**b)
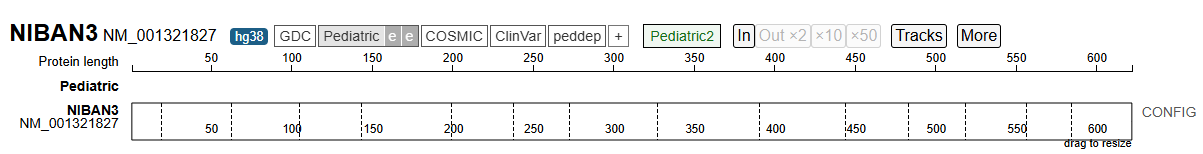
**

**
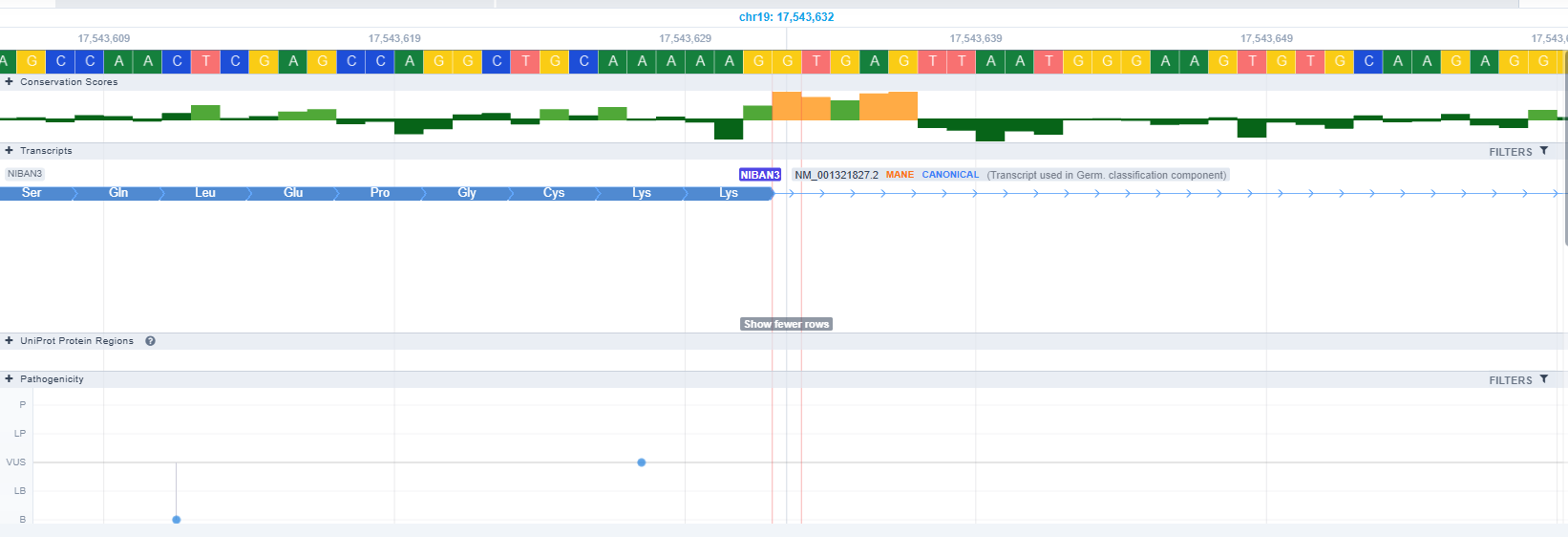
**

**Figure 18:** a) SpliceAI score of *BCNP1/NIBAN3* gene variant, **b**) This variant affects the canonical splice donor site at the beginning of intron 14 in the *BCNP1* gene. It is located in a critical splicing region, essential for proper mRNA processing. Disruption of this site may lead to aberrant splicing, potentially altering the expression or structure of the protein. (<https://pecan.stjude.cloud/variants/proteinpaint?gene=NIBAN3> ) (It is marked in red.)

**Case Vignette #15 (WES + WGS)**

Case #15 was diagnosed with B-ALL at the age of 4. The patient's maternal uncle passed away at the age of 15 due to lymphoma. Additionally, her maternal aunt was diagnosed with ovarian cancer at the age of 49, and her grandmother was diagnosed with breast cancer at the age of 45. We enrolled the patients to study with Jongman criteria 1. The initial WES analysis revealed a heterozygous frameshift deletion in the *BRAT1* c.1007delG p.G336fs. The variant was documented in the ClinVar database (ClinVar ID: 1453640) and classified as pathogenic with the evidence attributions of PVS1, PP5, PM2. Segregation analysis revealed that the mother was heterozygous and the father was WT. However, the affected maternal grandmother and maternal aunt were observed as WT. Hence, the decision was made for WGS, and in addition to the index and the parents, the maternal grandmother and the maternal aunt were also included in the joint analysis. WGS detected a heterozygous ***KSR1* c.527A>G p.Glu176Gly** variant. The variant has not been previously reported in the ClinVar database and is classified as a Likely Pathogenic (PM2, PP1) according to ACMG criteria. While the mother, affected grandmother, and affected aunt were observed as heterozygous, the father was WT.

a)


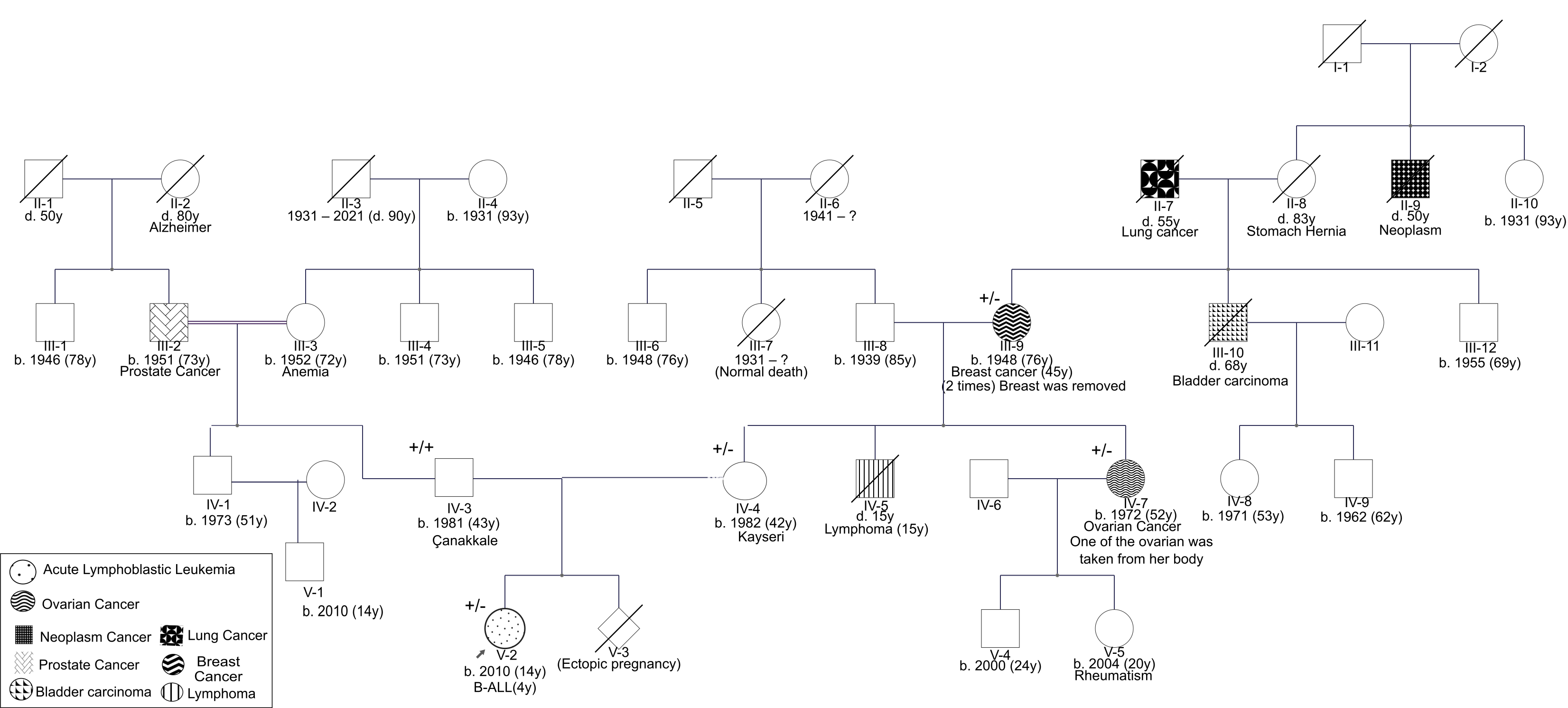


b)


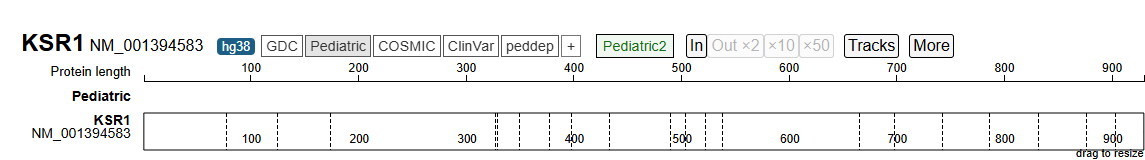


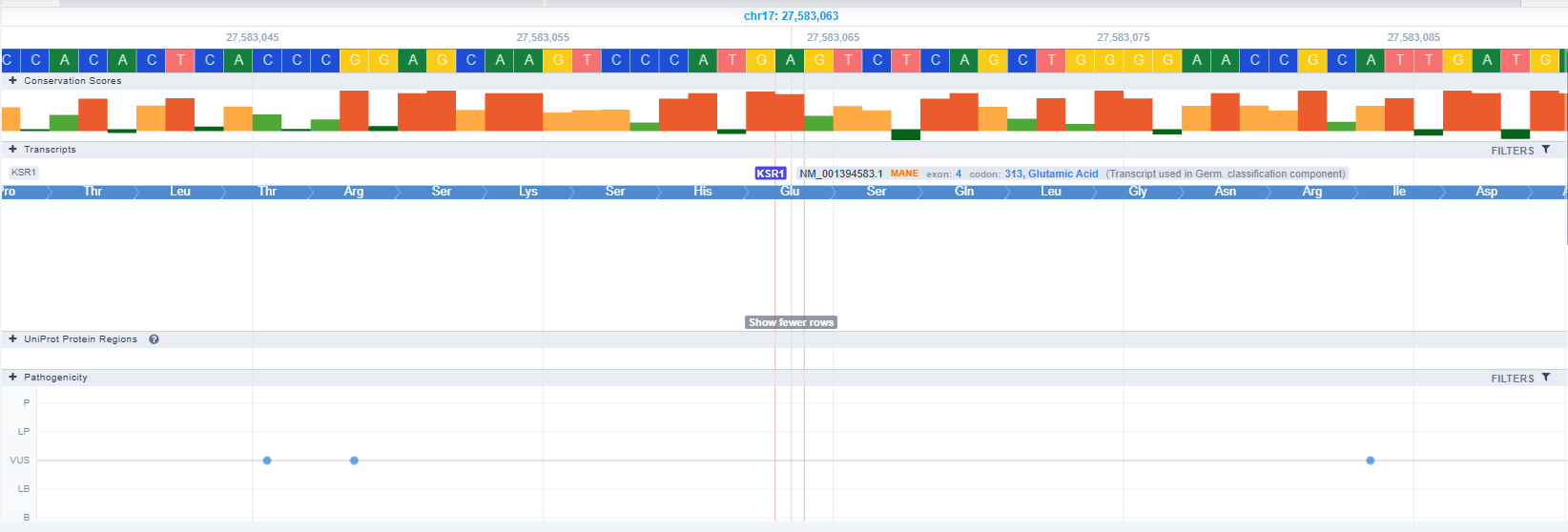
**Supp File 2. 19:** a) Case#15 pedigree (+/+Wild Type, +/- Heterozygous for *KSR1* gene variant**), b)** This missense variant is located in the *KSR1* gene and results in the amino acid change p.Glu176Gly. It falls within the N-terminal regulatory domain of the *KSR1* protein, which plays a role in scaffold-mediated regulation of the MAPK/ERK signaling pathway. This domain is essential for mediating protein-protein interactions with RAF and MEK, crucial for transmitting RAS signaling. Variants in this region may impair MAPK cascade regulation, contributing to oncogenic processes including leukemogenesis.

**Case Vignette #16 (WES + WGS)**

Case #16 was diagnosed with B-ALL at the age of 2. The parents had no consanguinity, but they were from the same town. The indexes’ maternal cousin was diagnosed with B-ALL at the age of 10, and the maternal uncle presented hepatosplenomegaly, but no confirmed diagnosis was reported. Additionally, the mother had a maternal cousin who died due to lymphoma at the age of 10. The combined WES analysis revealed a heterozygous *MUTYH* c.800C>T p.Pro267Leu (ClinVar ID: 185242) gene variant. Since there was no clinical correlation, we also performed a WGS analysis, and the same *MUTYH* variant was detected. No candidate variants suggestive of germline predisposition were detected in this patient or her affected cousin. This case remains unsolved. Additionally, family samples could not be found.


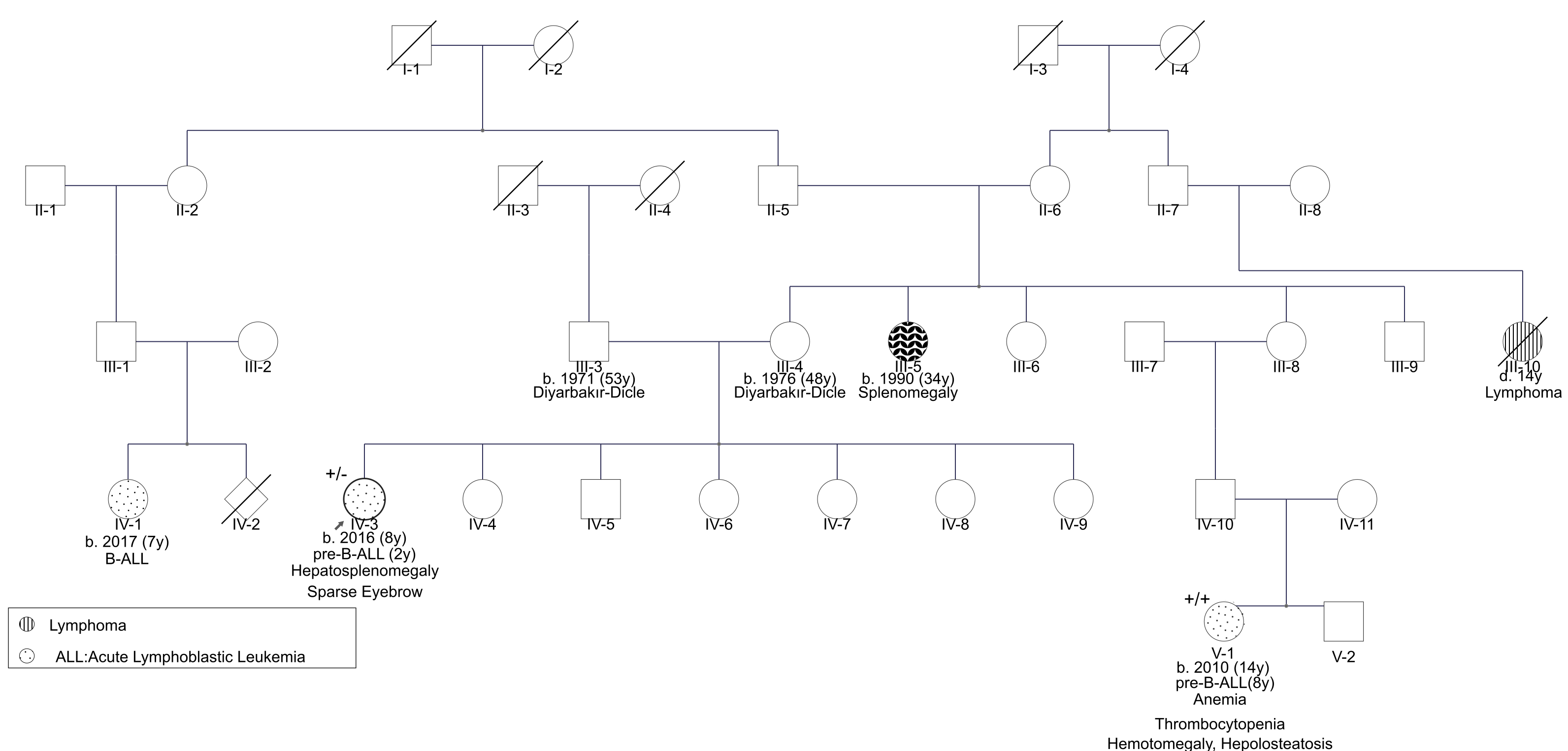


**Supp File 2. 20:** Case#16 pedigree (+/+ Wild Type, +/- Heterozygous)

**Case Vignette #17 (CES)**

Case #17 was diagnosed with non-Hodgkin Lymphoma when she was 5 years old. There is no consanguinity between the parents. The father's maternal grandmother (unknown age), the father's mother (51 years), and the father's aunt (58 years) were diagnosed with breast cancer. Additionally, the paternal grandfather’s niece and nephew were diagnosed with colon cancer and an unknown neoplasm at unknown ages. Also, the mother’s maternal grandmother was diagnosed with breast cancer but at an advanced age. Clinical exome sequencing detected a stop-gain heterozygous variant in ***the index in MLH1*** **c.1459C>T** **p.Arg487Ter**. (ClinVar ID: 89744). The variant has been classified as pathogenic according to ACMG criteria (PVS1, PP5, PM2). Based on the segregation analysis, it was detected that the variant was inherited from the father, and the mother was wild type. Since the other affected cases were deceased, no additional sample could be obtained for segregation analysis.


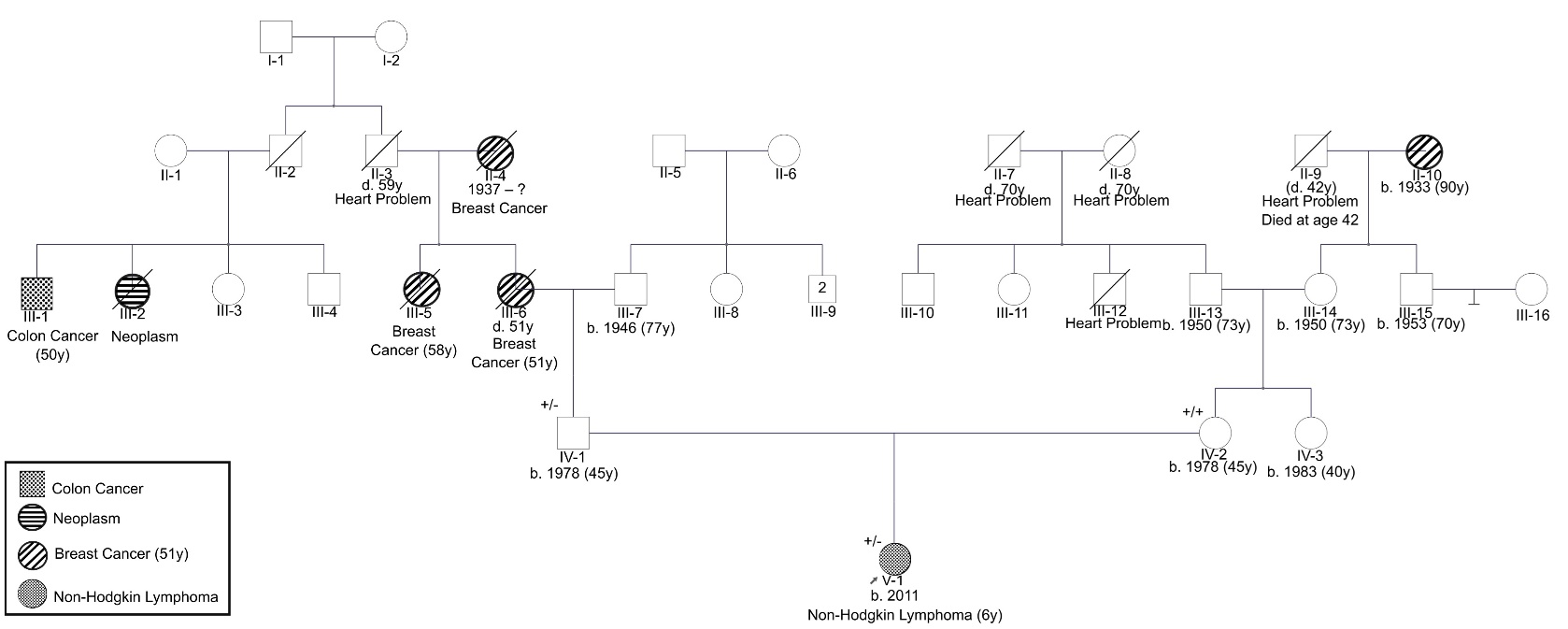


**Supp File 2. 21:** Case#17 Pedigree (+/+Wild Type, +/- Heterozygous)

**Case Vignette #18 (CES)**

Case #18 was diagnosed with B-ALL when he was 5 years old. There is no consanguinity between the parents. The maternal grandmother was diagnosed with breast cancer at the age of 45 and lung cancer at the age of 60. His maternal grandfather was also diagnosed with kidney cancer at an unknown age. Moreover, the mother’s paternal cousins were diagnosed with lung cancer, lung cancer accompanied by brain metastasis and an unknown neoplasm. We obtained samples from the index, the unaffected sibling and his parents, in addition to the affected maternal grandfather. The CES analysis revealed a heterozygous frameshift duplication variant ***BRCA1* c.5266dup: p.Gln1756ProfsTer74**. (**ClinVar ID**: 17677). The variant has been classified as pathogenic according to ACMG criteria (PVS1, PS3, PP5, PM2). Based on the segregation analysis, the variant was inherited from the mother, and the father was detected as wild type. Furthermore, we detected that the sister also inherited the variant, but the maternal grandfather, who was diagnosed with kidney cancer, was WT.


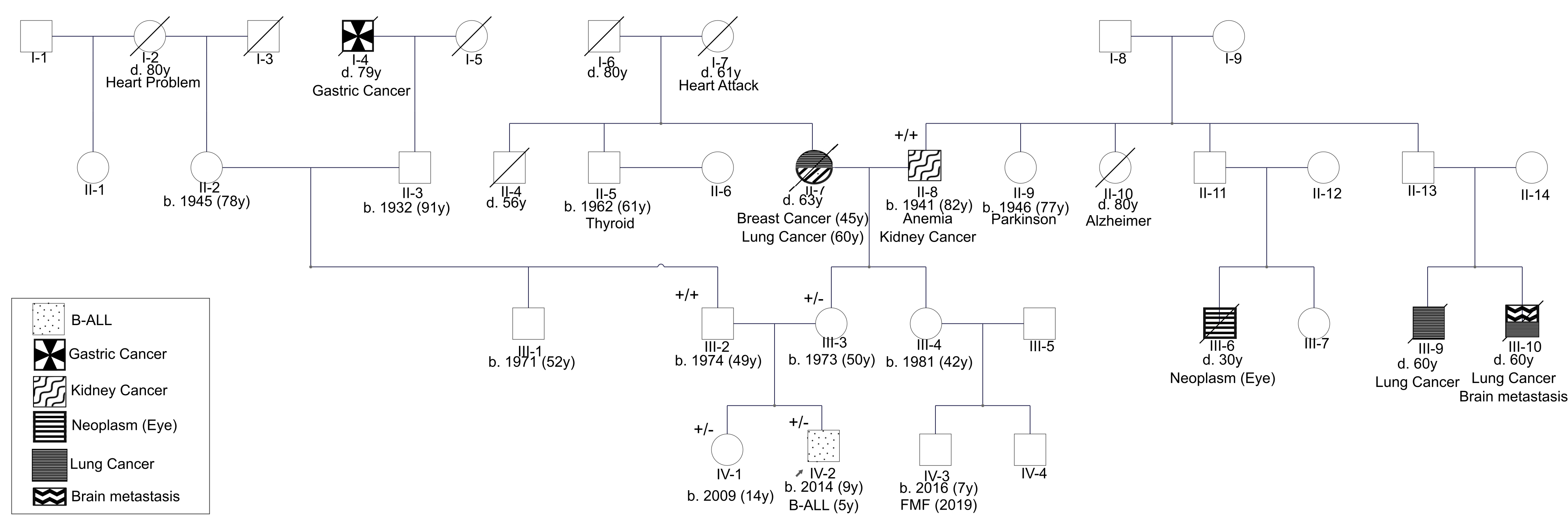


**Supp File 2. 22:** Case#18 pedigree (+/+Wild Type, +/- Heterozygous)

**Case Vignette #19 (CES)**

Case #19 was diagnosed with T-ALL at the age of 5. There was no consanguinity between the parents, and one of the paternal aunts was diagnosed with breast cancer (47 years), and another paternal aunt was diagnosed with kidney cancer at an unknown age. Moreover, the father’s maternal aunt was diagnosed with breast cancer, and the father’s paternal uncle was diagnosed with prostate cancer at an unknown age. The CES analysis detected a heterozygous ***BRCA2 c.3367A>G: p.Ser1123Gly*** (**ClinVar ID**: 51455) variant. The variant was classified as VUS according to ACMG criteria (PP3, PM2, BP1). Based on the segregation analysis, it was detected that the variant was inherited from the father, and the mother was wild type. Furthermore, the paternal aunt of the index case, who was diagnosed with kidney cancer, was also Sanger sequenced for the indicated variant; however, she was detected as wild type. No additional samples could be obtained for further segregation analysis


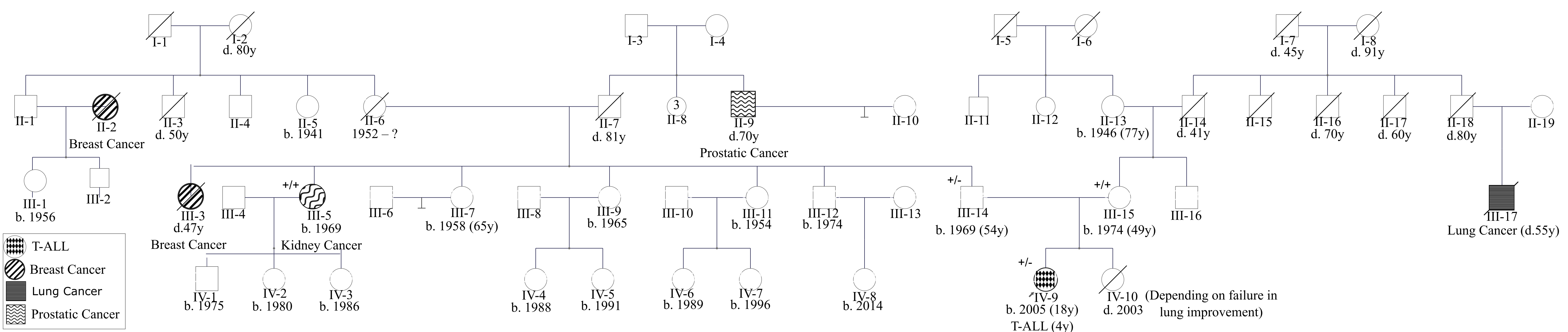


**Supp File 2. 23:** Case#19 Pedigree (+/+Wild Type, +/- Heterozygous)

**Case Vignette #20 (CES)**

Case #20 was diagnosed with B-ALL when she was 6 years old. There is no consanguinity between the parents. The father's paternal grandmother was diagnosed with breast cancer at an unknown age and died at the age of 56. The father’s paternal cousin died due to prostate cancer. The index’s paternal grandfather was diagnosed with lymphoma. Moreover, the index’s maternal aunt was diagnosed with breast cancer at the age of 48. The mother’s paternal uncle and maternal uncle died due to lung cancer at an advanced age. Mother’s maternal grandfather was diagnosed with colon cancer; he was treated, and he is alive. The CES analysis detected heterozygous *ABCC6* c.4070G>A: p.Arg1357Gln (**ClinVar ID**:1359254) variant. The variant has been classified as pathogenic according to ACMG criteria (PP3, PM5, PM2, PP5). Based on the segregation analysis, it was detected that the variant is inherited from the father, and the mother is detected as wild type. No additional samples could be obtained from the affected cases.


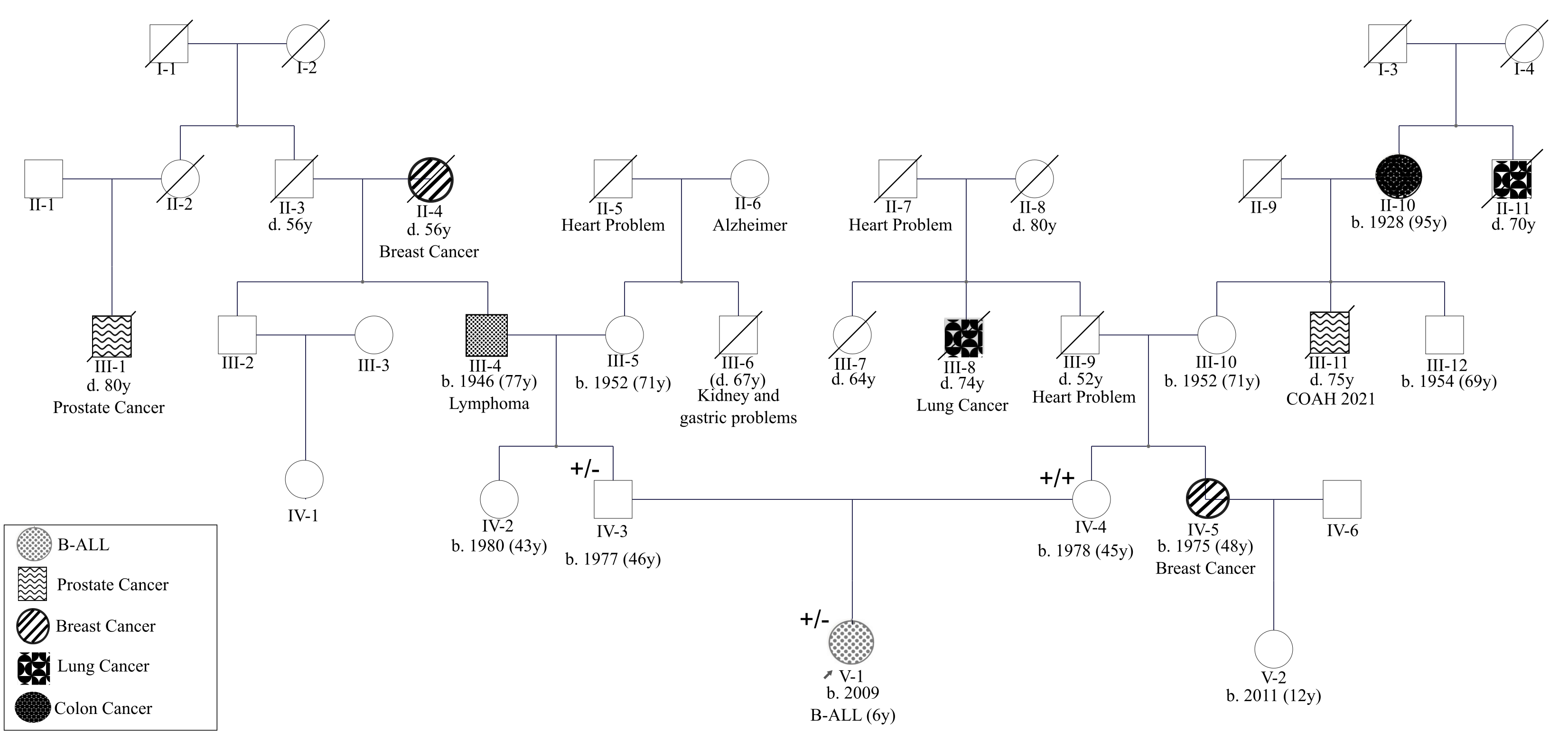


**Supp File 2. 24:** Case#20 pedigree (+/+Wild Type, +/- Heterozygous)
