# Supplementary material for: Genetic heterogeneity in childhood leukemia/lymphoma: a Turkish cohort with strong predisposition
Source: Front Genet. 2025 Sep 9;16:1624306. doi: 10.3389/fgene.2025.1624306 (PMC12454056; doi:10.3389/fgene.2025.1624306)
Supplement: Supplementary file 4 [file DataSheet3.pdf]

Paired-end Short Read Sequencing Analysis and Filtering

CES were studied with Sophia Genetics Clinical Exome Solution (CES_v2) kit that sequences 4490 clinically related genes' exons and flanking intronic regions. WES samples were sequenced on the Illumina Novaseq 6000 instrument s1 reagent kit v1.5. Fastq data obtained after CES/WES/WGS were processed at Acıbadem University Rare Diseases and Orphan Drugs Application and Research Center (ACURARE). The processed approach after CES/WES/WGS is given in Supplemental Figure 1A**.** For RNA sequencing analysis, following the quality control and quality trimming processes, data sets with sufficient quality were aligned to the human transcriptome reference sequence using STAR aligner. Following this process, read counts were determined using Deseq2, JunctionSeq and QoRT packages. RPKM (Read per kilobase million, TPM, Transcript per million (TPM) was used in the normalization of read count data, DESeq2/Junctionseq, edgeR were used in the differential expression and trimming analysis approaches. All these processes were performed within the Gennext platform we developed. RNA sequencing was performed only in case#13. (Supplemental Figure 1B)

The bioinformatics tools used in the .fastq data processing process developed by the ACURARE team are FASTQC, Burrows-Wheel Alignment (BWA) (The *hg38* (GRCh38) genome assembly was used for reference genome), Samtools, Picard, Parlement2, Genome Analysis Toolkit (GATK), Freebayes, BCFtools and VCFTools and Deepvariant. The advantage of using 4 different variant calling tools is to reduce the false positive rate. Pipeline is a tool that brings together frequently used tools for the analysis of NGS data and performs the entire NGS analysis process in an automated and fast manner. The pipeline is not only designed for WES data, it can handle WGS data as well. Availability to the GitHub link (https://github.com/AcuRARE/acugen). Annotation and filtering were done via **Gennext by Geniva** (https://gennext-edge.farplane.dev/server/nocache/login.html). The filtering approach primarily filters genes with frequent variations in hematological cancers, genes predisposed to hereditary cancers, and genes from syndromic diseases predisposed to cancer. The list of these genes consists of a combination of genes created within the Childhood Cancer Predisposition (ChiCaP) project at Karolinska and those known or suspected by us to be associated with hematological cancers. If no variant is detected in these three different progenitor gene lists, the filtering of the data is examined by generalizing. Variants present in the population database Genome Aggregation Database, with a minor allele frequency (MAF) less than or equal to 0.01 were maintained. Loss of function variants were manually examined with Integrative Genomics Viewer (IGV) looking for possible artifacts. In addition, to evaluate the potential impact of gene and variant pathogenicity, we combined the CADD (score $\geq$ 20), SIFT (score $<$0.05), POLPYHEN (score $\geq$0.7) REVEL (score $\geq$ 0.5), M-CAP (score $\geq$ 0.025), GERP++ (score $>$ 2.5) algorithms.

The WGS took place at the **Science for Life Laboratory (SciLifeLab)** in Stockholm, Sweden. Libraries were prepared by using Illumina TruSeq PCR-free kit. Paired-end reads with average coverage of 30X

were obtained on the HiSeq X instrument (Illumina, CA, USA). The data were processed using in-house pipeline. Burrow-Wheeler Aligner6 was used for mapping reads to the reference human genome (GRCh37). Genome Analysis Toolkit7 was used for duplicate marking, variant calling and joint genotyping. Variants were annotated using Variant Effect Predictor8 and then loaded into a database generated by GEMINI.9 Rare variants (MAF <0.01 in publicly available population databases ExAC, gnomAD and SweGen) in the coding sequences and splice sites that supported autosomal recessive inheritance model were prioritised. The WGS filtering occurred on the Scout (https://github.com/Clinical-Genomics/scout) and Gennext data analysis platform.
Furthermore, variants classified as Likely Benign/Benign by Clinvar were excluded from further analysis. Finally, the remaining variants were classified according to the ACMG–AMP (American College of Medical Genetics and Genomics/Association for Molecular Pathology) guidelines as pathogenic(P), likely pathogenic (LP), variant uncertain significance (VUS) (Richard et al., 2015).Varsome, Franklin and Intervar tools were used for variant interpreperation and classification.

Sequencing data of the 20 index were analyzed using a list of 2887 genes associated with childhood cancer predisposition for variant prioritization. while all indexes were analyzed independently from the gene list. Index cases and the affected family members were also analyzed individually.The filtering approach is given in Supplemental Figure 2**.** Our standard filtering approach is "One Fits All," aiming to develop broad filtering criteria that are generally applicable to a variety of genetic analyses. This approach seeks to enhance efficiency in genetic data analysis and standardize the analysis processes. Therefore, filtering approach begins with a list of genes known or thought to be associated with leukemia and lymphoma. This gene list includes genes associated with hereditary cancer syndromes, cancer predisposition genes, and genes identified by the Childhood Cancer Predisposition (ChiCaP) project of the Karolinska Institute, for a total of 2887 genes. Variants defined in ClinVar are initially filtered. The filtered SNVs/INDELs and SVs were classified according to the ACMG guidelines as P, LP, and VUS [16]. If no defined variants are found, variants classified as Pathogenic, Likely Pathogenic, and Variant of Uncertain Significance according to the American College of Medical Genetics and Genomics (ACMG) criteria are included, while Benign and Likely Benign variants are excluded. Filtering is based on the variant type (exonic, splicing), zygosity (homozygous, heterozygous, hemizygous), population frequency (Minor Allele Frequency <0.01) (GNOMAD, Iranome, Turkish Variome, GME project, 1000K), the gene's evolutionary conservation (GERP++, PhyLoP, PhastCons, Gene Ontology, etc.), and in silico prediction tools (SIFT, Polyphen-2, CADD, MutPred, SpliceAI, VEST4, REVEL, MetaLR, ClinPred, FATHMM, Mutation Assessor, etc.) to determine the pathogenicity of the protein change. Outside the "One Fits All" approach, we implement a customized filtering approach, supported by over 150 annotator options. A custom annotator is used for each specific case. Custom annotators are tailored and developed to meet the requirements of a particular study or analysis. They are used for various tasks, such as identifying the characteristics of genetic variants, making pathogenicity predictions, evaluating

their clinical significance, or associating them with specific genetic phenotypes. If candidate gene variants cannot be correlated with clinical presentation, an expanded filtering approach is applied.

Our expanded filtering approach proceeds without a predefined gene list. In these gene-list-free filtering steps, we also consider splice site variants, UTR and intronic regions with phenotype-associated variants, copy number variants (CNV), and structural variants (Structural Variant Length <5000), with a slightly higher population frequency to identify phenotype-associated variants. For cases where candidate variants cannot be identified using our filtering approach, reanalysis is performed every six months or 1 year. Filtering is performed separately for each case, both with and without a predefined gene list.

All gene variants were checked against the St.Jude Pediatric Cancer (PeCan) Cloud database (https://pecan.stjude.cloud/variants/proteinpaint/ST). The PeCan platform presents curated pediatric cancer genomics data including variants, mutational signatures, and gene expression data in addition to histological slide images from ~9000 hematological, CNS, and non-CNS solid tumor patient samples. The frequency of occurrence of the candidate variants and the frequency of occurrence in the Türkiye population was determined by the data set from the previously published study that was conducted on *3,362* (WES; *n = 2,589*, and WGS; *n = 773*) unrelated Türkiye originated individuals. This study was used as Turkish (TR) Variome data that should facilitate the discovery of disease genes in Türkiye (Kars et al., 2021). The cases participated in genetic studies of non-malignant (amyotrophic lateral sclerosis, ataxia, delayed sleep phase disorder, essential tremor, obesity, Parkinson's disease, polycystic ovarian syndrome, and various assorted neurological and immunological) disorders.

SpliceAI score

SpliceAI annotates genetic variants with their predicted effect on splicing (https://spliceailookup.broadinstitute.org/) (Jaganathan et al., 2019) The Delta score for a variant ranges from 0 to 1 and is interpreted as the probability that the variant alters the splicing. The closer to 1, the high sensitivity. The Delta position conveys information about the location where the splicing changes relative to the variant position (positive values are downstream of the variant, negative values are upstream). This score was calculated for *ETV6, TNFRSF9 and BCNP1* splice site variants (Supplementary File 2).

VAMPP (Variant Analysis with Multiple Pathogenicity Predictors)-score

We used the VAMPP-score, a new statistical framework designed to evaluate missense variants developed by our team. The VAMPP score leverages the best gene-in silico pathogenicity predictors (ISPPs) matches based on ISPPs prediction accuracies and provides a combinatorially weighted score

that improves missense variant interpretation (Ozdemir et al., 2024). On the other hand, For the variants with a cut-off score of >0.35, the PP3 moderate evidence can be applied. (https://vamppscore.com/)

Predicted Local Distance Difference Test (pLDDT) score and ΔΔG score

We have also analysed the variant effect on protein level by AlphaFold protein structure databases (https://alphafold.ebi.ac.uk/) (Jumper et al., 2021) The Predicted Local Distance Difference Test (pLDDT) score indicates the reliability (confidence) of the predicted regions of a protein structure. The score ranges from 0 to 100 and is calculated for each amino acid residue individually. pLDDT provides a confidence level for how accurate the predicted structure is. For each of the candidate variants, pLDDT scores for protein changes were calculated using AlphaFold. Regions with *very high* reliability scores indicate a correct and stable structure. Protein changes in the *confident* group suggest that the folding is largely accurate. In contrast, changes with *low* scores provide insight that these regions are likely to have flexible structures. High pLDDT scores indicate stable regions within the protein structure, while low scores correspond to flexible or disordered regions (Supplementary File 3). AlphaMissense analyses is an artificial intelligence (AI)-based bioinformatics tool used to predict whether missense variants (SNVs) are pathogenic or benign. This model analyzes protein structure, evolutionary conservation, and biochemical properties using AlphaFold and large-scale protein databases. (https://alphamissense.hegelab.org/) AlphaMissense generates a score between 0 and 1 for each variant: <0.5 means it probably does not harm the protein's function. 0.5 - 0.7 means its effect is not known for certain, additional data may be needed. 0.7 - 1.0 means it probably disrupts the protein's structure or function, leading to disease. However, the AlphaMissense score alone is not sufficient for clinical interpretation.

DynaMut2 (https://biosig.lab.uq.edu.au/dynamut2/), is a computational bioinformatics tool used to predict the impact of protein mutations on structural stability and dynamics. It integrates molecular dynamics simulations and energy calculations to assess the effects of protein mutations on thermal stability, conformational changes, and flexibility. By leveraging pre-trained models such as FoldX and mCSM, it calculates the effect of mutations on free energy change (ΔΔG). If the ΔΔG value is negative, the mutation is destabilizing, indicating a decrease in stability. In this context, the ΔΔG value was calculated for 15 missense variants.

Network analysis

The tissue specific protein-protein interaction (PPI) of the identified candidate genes was performed by Network analysis (https://www.networkanalyst.ca/NetworkAnalyst/). In string analysis, significance in PPI network is assessed by PPI enrichment p-value. Generally, an interaction is considered significant when this value is <0.05 (This p-value indicates whether the number of observed interactions is significant compared to the expected number of random interactions). Additionally, separate analyses

were conducted for the genes identified in leukemia and lymphoma cases (Supplementary Figure 3). In the network analysis, KEGG (Kyoto Encyclopedia of Genes and Genomes) and GO:BP (Gene Ontology: Biological Process) databases were used as function explorers.
