# Supplementary material for: Genetic heterogeneity in childhood leukemia/lymphoma: a Turkish cohort with strong predisposition
Source: Front Genet. 2025 Sep 9;16:1624306. doi: 10.3389/fgene.2025.1624306 (PMC12454056; doi:10.3389/fgene.2025.1624306)
Supplement: Supplementary file 5 [file DataSheet1.pdf]

A)

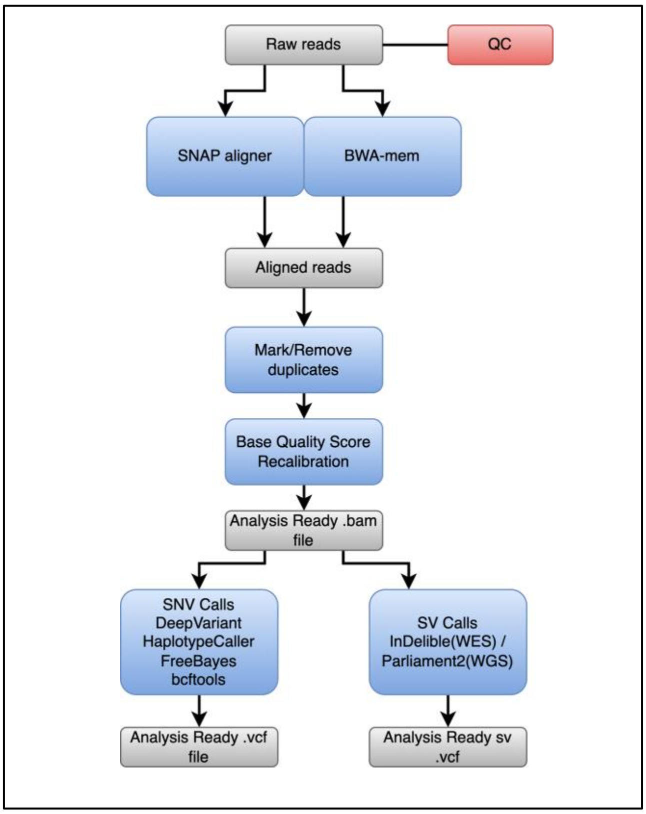

B)

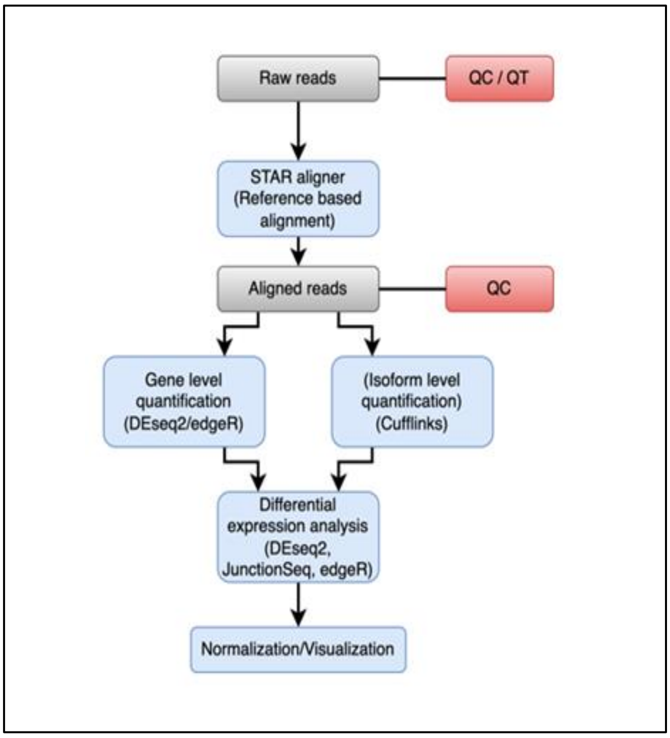

Supplemental Figure 1: A) CES (Clinical Exome Sequenicng) /WGS (whole genome sequencing) /WES (whole exome sequencing) workflow; B) RNA sequencing workflow (QC: Quality Control, QT: Quantitative Transcriptome, SNAP: Scalable Nucleotide Alignment Program, DeSeq2: An Overview of

a Popular RNA-Seq Analysis Package, edgeR: differential analysis of sequence read count data, STAR:Spliced Transcripts Alignment to a Reference, SNV: Single Nuclotide variation, CNV: Copy Number Variation, BWA-Mem: Burrows-Wheeler Aligner, vcf: variant call format)
