# Supplementary material for: Genetic heterogeneity in childhood leukemia/lymphoma: a Turkish cohort with strong predisposition
Source: Front Genet. 2025 Sep 9;16:1624306. doi: 10.3389/fgene.2025.1624306 (PMC12454056; doi:10.3389/fgene.2025.1624306)
Supplement: Supplementary file 6 [file DataSheet5.pdf]

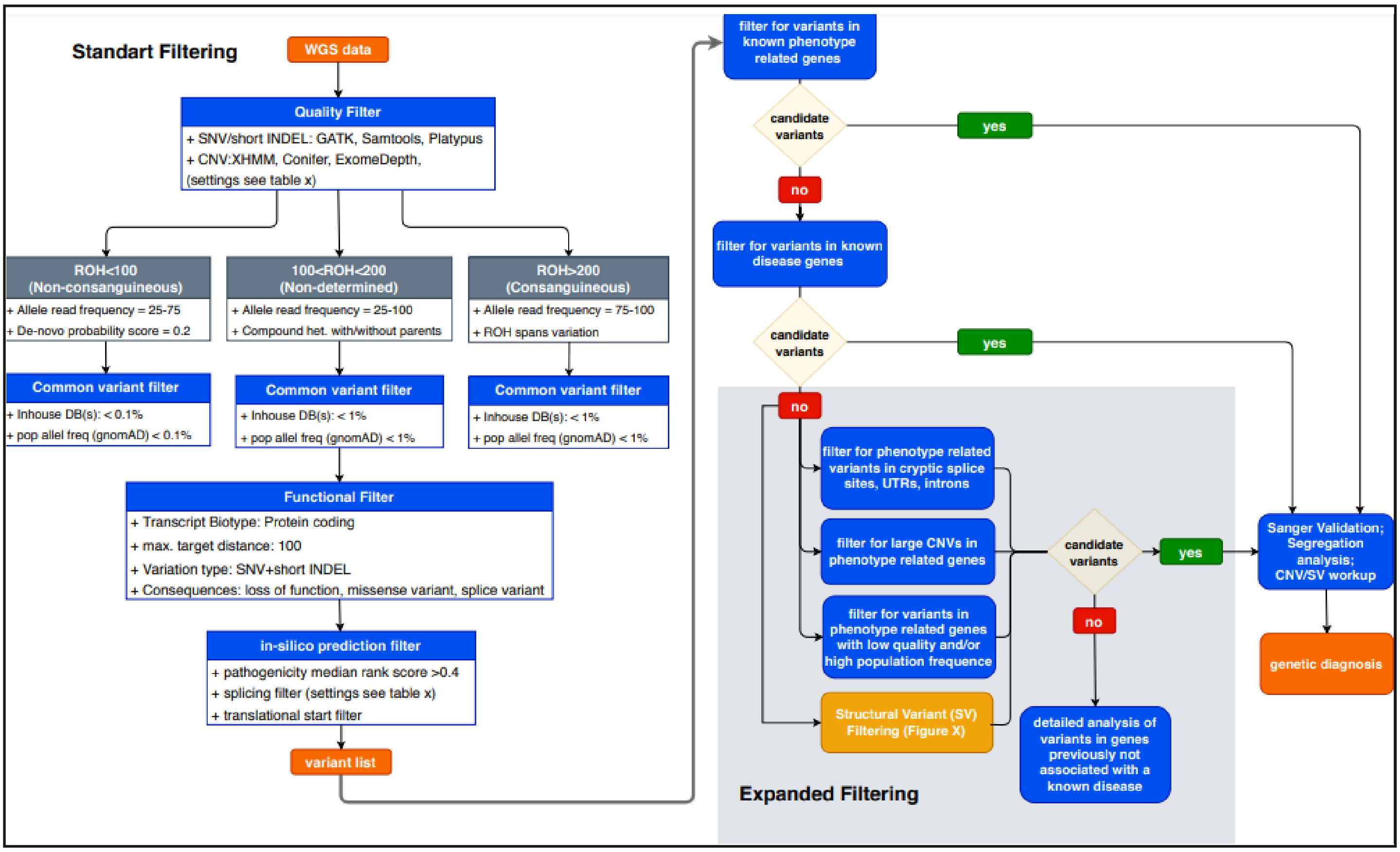

Supplemental Figure 2: Filtering approach for NGS data (SNV: Single Nücleotide Variation, INDEL: Insertion or deletion, CNV: Copy Number Variation, ROH: Runs of homozygosity, SV: structural variant, GATK:Genome Analysis Tool kit, XHMM:eXome-Hidden Markov Model, ROH: Runs of Homozygosity, DB: Databases)
